# Unnatural activities and mechanistic insights of cytochrome P450 PikC gained from site-specific mutagenesis by non-canonical amino acids

**DOI:** 10.1038/s41467-023-37288-0

**Published:** 2023-03-25

**Authors:** Yunjun Pan, Guobang Li, Ruxin Liu, Jiawei Guo, Yunjie Liu, Mingyu Liu, Xingwang Zhang, Luping Chi, Kangwei Xu, Ruibo Wu, Yuzhong Zhang, Yuezhong Li, Xiang Gao, Shengying Li

**Affiliations:** 1 State Key Laboratory of Microbial Technology, Shandong University, Qingdao, Shandong 266237 China; 2 Laboratory for Marine Biology and Biotechnology, Qingdao National Laboratory for Marine Science and Technology, Qingdao, Shandong 266237 China; 3 School of Pharmaceutical Sciences, Sun Yat-sen University, Guangzhou, 510006 China

**Keywords:** Enzymes, X-ray crystallography, Biocatalysis, Enzyme mechanisms

## Abstract

Cytochrome P450 enzymes play important roles in the biosynthesis of macrolide antibiotics by mediating a vast variety of regio- and stereoselective oxidative modifications, thus improving their chemical diversity, biological activities, and pharmaceutical properties. Tremendous efforts have been made on engineering the reactivity and selectivity of these useful biocatalysts. However, the 20 proteinogenic amino acids cannot always satisfy the requirement of site-directed/random mutagenesis and rational protein design of P450 enzymes. To address this issue, herein, we practice the semi-rational non-canonical amino acid mutagenesis for the pikromycin biosynthetic P450 enzyme PikC, which recognizes its native macrolide substrates with a 12- or 14-membered ring macrolactone linked to a deoxyamino sugar through a unique sugar-anchoring mechanism. Based on a semi-rationally designed substrate binding strategy, non-canonical amino acid mutagenesis at the His238 position enables the unnatural activities of several PikC mutants towards the macrolactone precursors without any sugar appendix. With the aglycone hydroxylating activities, the pikromycin biosynthetic pathway is rewired by the representative mutant PikC_H238*p*AcF_ carrying a *p-*acetylphenylalanine residue at the His238 position and a promiscuous glycosyltransferase. Moreover, structural analysis of substrate-free and three different enzyme-substrate complexes of PikC_H238*p*AcF_ provides significant mechanistic insights into the substrate binding and catalytic selectivity of this paradigm biosynthetic P450 enzyme.

## Abstract (web summary)

The 20 proteinogenic amino acids cannot always satisfy the requirements of protein engineering. Here, the authors practice semi-rational non-canonical amino acid mutagenesis for a cytochrome P450 enzyme, leading to unnatural activities and significant mechanistic insights.

## Introduction

The ability of microorganisms to rapid evolve resistance to antibiotics warrants endless requirements for discovering and engineering new antimicrobial agents^1^. Macrolide antibiotics with the hallmark feature of a variably sized (≥12 carbons) macrolactone ring connected to a deoxy and/or amino-sugar moiety via glycosylic bond have demonstrated remarkable therapeutic applications as effective protein synthesis inhibitors^2^. To address the central issues of side effects and antibiotic resistance, three generations of macrolides have been developed in the past century. Nowadays, macrolide antibiotics are still widely used in the treatment of infectious^2,3^, respiratory^4^, digestive^5^, and other diseases. In particular, the third generation of macrolides, namely ketolides (with the 3-glycosylated hydroxyl group replaced by a 3-keto group), have shown enhanced activities against many macrolide-resistant pathogens^6^.

**Fig. 1:**
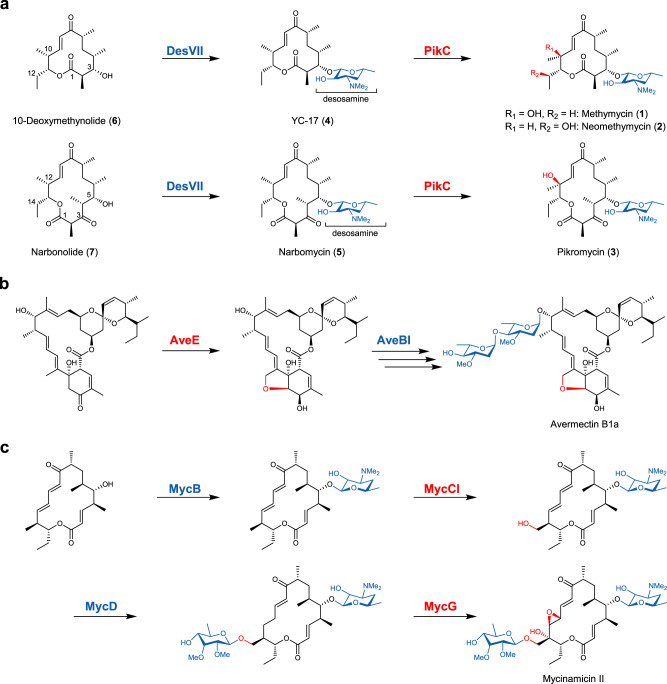
Representative macrolide biosynthetic pathways. **a** The late pikromycin/(neo)methymycin biosynthetic pathway in which the glycosyltransferase-mediated sugar attachment precedes the P450-catalyzed hydroxylation. **b** The partial avermectin biosynthetic pathway with an order of oxidation-glycosylation. **c** The post-PKS pathway of mycinamicin biosynthesis with staggered monooxygenation and glycosylation steps. The functional groups introduced by P450 are highlighted in red, and the functional groups introduced by glycosyltransferase are highlighted in blue.

During the biosynthesis of diverse macrolide natural products, after assembly of the macrolactone core structure by modular type I polyketide synthases (PKSs), glycosylation and oxidation that are mediated by various glycosyltransferases (GTs; e.g., UGTs, UDP-dependent GTs) and cytochrome P450 monooxygenases (P450s), respectively, are among the most important post-PKS tailoring modifications^7,8^. Interestingly, the order of these two types of structural decoration largely varies in different macrolide biosynthetic pathways (Fig. 1, Supplementary Fig. 1). For example, the glycosylation step must occur prior to the P450-catalyzed oxidation in the biosynthesis of pikromycin^7,9^ (Fig. 1a) and rosamicin^10^ (Supplementary Fig. 1a). By contrast, the P450-mediated transformation precedes the glycosylation in many cases such as avermectins^11^ (Fig. 1b) and disciformycins^12^ (Supplementary Fig. 1b). More intriguingly, the monooxygenation and glycosylation steps even stagger in the mycinamicin^13,14^ (Fig. 1c) and erythromycin^15^ (Supplementary Fig. 1c) biosynthetic pathways. It is noteworthy that the biosynthetic order in most of these cases cannot be reversed, indicative of the stringent substrate specificities of the involved P450s and GTs. The irreversible order likely endows the antibiotic producer organisms with evolutionary benefit by directing the metabolic flux to a single or a limited number of product(s) with high potency. However, from the standpoint of structural diversification for new drug development, the frozen biosynthetic order is a limiting factor because it would hamper more extensive pathway reprogramming and combinatorial biosynthesis^16^. To resolve this problem, the substrate specificity of GTs and P450s need to be changed simultaneously, which represents a substantial biocatalytic challenge.

Comparatively, we reason that the order of oxidation-glycosylation might be advantageous over that of glycosylation-oxidation with respect to product structural diversification because the preceding oxidation (as a small structural modification) is unlikely to impede the following glycosylation(s). Moreover, the oxidation in some cases could generate a new chemical handle for further glycosylation(s), e.g., mycinamicins (Fig. 1c) and disciformycins (Supplementary Fig. 1b). However, the sugar attachment by GTs would cause a significant increase of both the size and polarity of the resulting macrolide, thus limiting the potential oxidation by a P450 enzyme that originally favors the smaller hydrophobic macrolactone substrate.

Cytochrome P450 PikC (CYP107L1) from *Streptomyces venezuelae* ATCC 15439 is an intriguing P450 monooxygenase with unusual innate substrate flexibility^17,18^. In the biosynthetic pathway of methymycin/neomethymycin (**1**/**2**) and pikromycin (**3**, the first discovered natural ketolide), the sole P450 enzyme recognizes both 12-membered ring macrolide YC-17 (**4**) and 14-membered ring macrolide narbomycin (**5**) as substrates (Fig. 1a). Comparatively, PikC catalyzes the hydroxylation of **4** at the C10 allylic position and the C12 methylene position with almost equal efficiency, while the PikC-mediated hydroxylation of **5** preferentially occurs at the C12 allylic position^19,20^. In the past two decades, Sherman and co-workers have made tremendous efforts on the structures, mechanisms, and engineering of PikC^21–24^, making this enzyme a paradigm of microbial P450 enzyme.

Although the substrate scope and product diversity of PikC have been dramatically expanded by enzyme engineering and substrate engineering^21–24^, to the best of our knowledge, there is no report of any PikC mutant that is able to directly oxidize the two native macrolactones including 10-deoxymethonolide (**6**) and narbonolide (**7**), likely due to the very poor binding of these two unglycosylated substrate precursors to PikC. This is mechanistically owing to the dependence of the dimethylamino sugar desosamine in both **4** and **5** during substrate recognition and productive binding by PikC. To date, it has been well known that the presence of the positively charged dialkylamino group as a substrate anchor is a prerequisite for PikC’s substrate recognition and catalytic activities^17,21^.

To further diversify the structures related to the **1**/**2**/**3** biosynthetic pathway by reversing the natural glycosylation-oxidation order, in this study, we challenge the unnatural activity of PikC against the macrolactones without any appendix. Taking advantage of the non-canonical amino acids (ncAAs), we successfully reshape the sugar binding pocket of PikC in a manner that the proteinogenic amino acid mutagenesis cannot achieve. A number of mutants with the pivotal residue His238 replaced by different ncAAs show significant hydroxylation activities towards the aglycones **6** and **7**. X-ray crystal structures and molecular docking analyses of the optimal mutant PikC_H238*p*AcF_ in substrate-free and substrate-bound forms provide significant insights into the mechanisms of PikC’s substrate recognition and regioselectivity. Furthermore, the oxidation activity towards **6** and **7** enable us to change the order of late pikromycin biosynthetic pathway, giving rise to unnatural mono- and di-glucosylated macrolide derivatives.

## Results

### Semi-rational engineering of PikC into an aglycone hydroxylase by ncAA mutagenesis

**Fig. 2:**
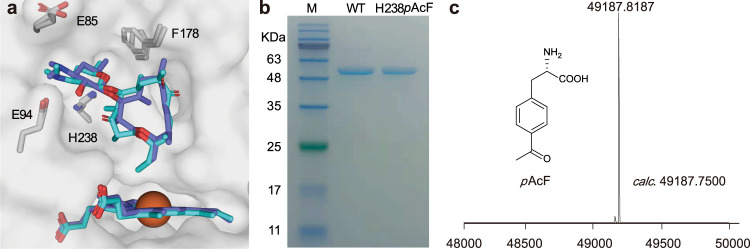
Design and preparation of PikC_H238*p*AcF_. **a** Overview of the superimposed PikC active sites with productive binding of **4** and **5** (PDB ID codes: 2VZ7 and 2VZM). The heme group and amino acid residues targeted for mutagenesis are displayed as sticks. Substrates **4** and **5** are shown in slate and cyan, respectively. The iron atom is shown as sphere. **b** SDS-PAGE analysis of purified PikC_WT_ and PikC_H238*p*AcF_. Source data are provided as a Source Data file. **c** ESI-TOF MS analysis of the purified PikC_H238*p*AcF_ protein. A peak measured at 49187.8187 Da corresponds to PikC_H238*p*AcF_ lacking the initiating methionine residue (the calculated mass of PikC_H238*p*AcF_: 49187.7500 Da).

According to the previous structural and mechanistic studies^19,20,23^, the sugar (desosamine) and aglycone (**6** and **7**) moieties of PikC’s natural substrates (**4** and **5**) adopt distinct binding pockets (Fig. 2a). To enable the productive binding of either **6** or **7** to PikC, we reasoned that the introduction of a rather bulky amino acid to occupy the accommodating space of desosamine might work. Moreover, this amino acid should endow the aglycones with some specific interactions, thereby increasing the substrate binding affinity to a productive level. Thus, we first identified the amino acids within 4.0 Å away from the desosamine of either substrate, including Glu85, Glu94, Phe178 and His238 (Fig. 2a). Next, the ncAA *p-*acetylphenylalanine (*p*AcF), which was previously reported to show beneficial effects on P450’s efficiency and regioselectivity^25^, was selected to individually replace the four residues by amber stop codon suppression and the well-established system of mutant *Methanococcus jannaschii* tyrosyl aaRS/tRNA pair in *Escherichia coli*^26^. We expected that the unnatural bulky acetylphenyl side chain with *π*-stacking and hydrogen bonding potential would not only sterically occupy the desosamine binding pocket, but also provide some favorable interactions with the macrolactone ring of **6** or **7**, thus enabling the unnatural aglycone hydroxylation activities.

**Fig. 3:**
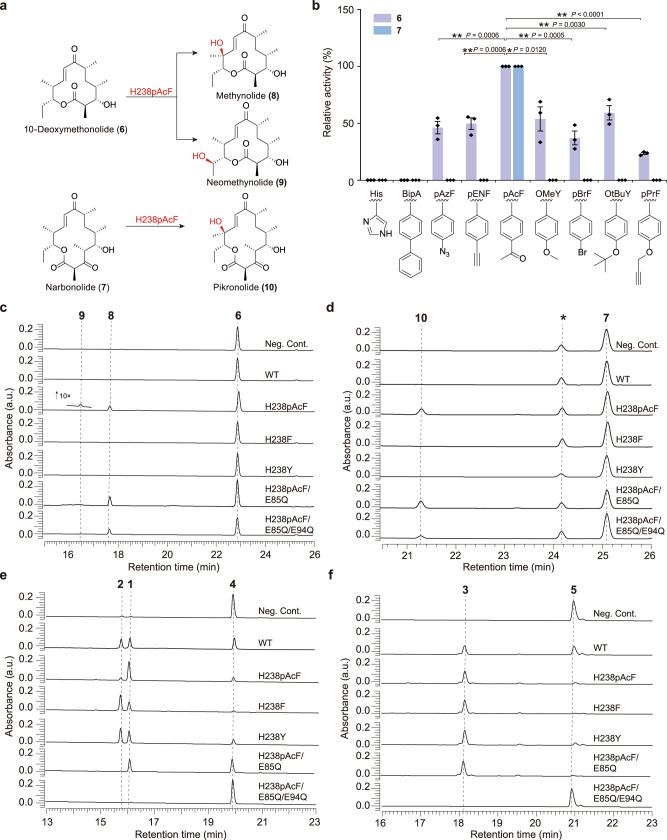
Functional analysis of PikC mutants. **a** The scheme of PikC_H238*p*AcF_ catalyzed aglycone hydroxylation reactions. **b** The relative activities of the mutants with His238 replaced by different ncAAs towards **6** and **7**. Data are presented as mean values ± SEM. Black diamond-dots represent individual data points for *n*  =  3 replicates. Statistical analysis was performed using the Student’s *t* test (two-tailed; *P < 0.05, **P < 0.01). **c**–**f**, HPLC analysis (230 nm) of the enzymatic reactions catalyzed by the wild type (WT) and indicated mutant PikC enzymes using **6** (**c**), **7** (**d**), **4** (**e**), and **5** (**f**) as substrates. Each individual in vitro enzymatic reaction containing 1 µM PikC (wild type or mutant), 1 mM NADPH, 10 µM Fdx1499, 5 µM FdR0978, and 0.5 mM substrate in 100 μL storage buffer was incubated at 30 °C for 40 min. The peaks marked by asterisk in (**d**) denote the spontaneous decomposition product of **7**.

To our delight, *p*AcF was successfully incorporated into PikC with high efficiency as evidenced by tandem mass spectrometry (Supplementary Fig. 2) and electrospray ionization-quadrupole-time of flight-mass spectrometry (ESI-Q-TOF-MS) analysis of the purified mutant P450 enzyme PikC_H238*p*AcF_ (Fig. 2b, c). The two macrolactone substrates **6** and **7** were prepared using the glycosyltransferase DesVII inactivated strain *Sv*-*ΔdesVII* (see Methods). Among the four confirmed *p*AcF mutants including PikC_E85*p*AcF_, PikC_E94*p*AcF_, PikC_F178*p*AcF_ and PikC_H238*p*AcF_ (Supplementary Figs. 3–5), PikC_H238*p*AcF_ turned out to be the only mutant to show a low but detectable activity towards both **6** and **7** with the respective substrate conversion ratios of 17.9 ± 2.4% and 24.7 ± 3.0% (Supplementary Fig. 6). The low but significant activities of this ncAA mutant might result from the challenging nature of C-H bond activation and low binding affinity towards aglycones (*see below*). Upon high-resolution mass spectrometry (HRMS) and nuclear magnetic resonance (NMR) analyses (Supplementary Figs. 9–22, 35–39), the structures of the two products from **6** were determined to be methynolide (**8**, the C10-hydroxylated product) and neomethynolide (**9**, the C12-hydroxylated product) (Fig. 3a) with a relative ratio of 19:1 (Fig. 3c). The single hydroxylation product of **7** was determined to be pikronolide (**10**, the C12-hydroxylated product) (Fig. 3a, d). Interestingly, tiny amounts of **8**–**10** were previously isolated from another *S. venezuelae* strain^27^ and a *desI* (the sugar dehydrase gene) knockout mutant^28^, perhaps due to the marginal activity of PikC_WT_ or other unknown oxidase towards **6** and **7** in vivo. However, the hypothetical activities of PikC_WT_ against **6** and **7** were not observed in vitro (Fig. 3c, d). Of note, all 20 natural amino acids at the His238 position were incapable of reproducing the aglycone hydroxylation activity caused by *p*AcF (Supplementary Fig. 7).

### Unexpected discovery of the improved activities to native substrates by aromatic substitution at His238

Strikingly, compared with the activities of PikC_WT_ towards **4** (45.0 ± 4.5% conversion) and **5** (54.4 ± 2.8%), PikC_H238*p*AcF_ exhibited 0.9- and 0.7-fold increase in the hydroxylation activity towards the natural substrates **4** and **5**, respectively (Fig. 3e, f). Unlike the wild-type enzyme that almost equally hydroxylates C10 and C12 positions of **4**, PikC_H238*p*AcF_ regioselectively hydroxylated the C10 C-H bond, producing **1** as the major product (Fig. 3e, **1** : **2** = 9 : 1). Inspired by these unexpected results, we examined the activities of twenty proteinogenic variants of His238 towards **4** and **5**. As a result, these mutants exhibited varied activities and product distributions against **4** with H238F and H238Y being the most active mutants. Specifically, the substrate conversion ratios of PikC_H238F_ and PikC_H238Y_ towards **4** were 93.2 ± 5.3% and 83.8 ± 3.9%, respectively. However, these two mutants did not significantly change the product ratio as PikC_H238*p*AcF_ did (Fig. 3e, Supplementary Fig. 51). As for **5**, PikC_H238F_ and PikC_H238Y_ were also the most efficient mutants with the substrate conversion ratios of 96.3 ± 1.7% and 88.3 ± 3.3%, respectively (Fig. 3f, Supplementary Fig. 51). Taken together, we surmised that the presence of an aromatic side chain at the His238 position might be essential for the improvement of native activities of PikC. However, as mentioned above, PikC_H238F_ and PikC_H238Y_ were unable to oxidize **6** and **7** (Fig. 3c, d), suggesting that the aglycone hydroxylation activity of PikC_H238*p*AcF_ should stem from a mechanism different from that for the enhanced activities towards **4** and **5**.

### Determination of substrate binding affinities

To rationalize the enhanced activity of PikC_H238*p*AcF_ towards **4**–**7**, we compared the substrate binding affinities of PikC_WT_ and PikC_H238*p*AcF_ by measuring the spectral changes upon different substrate titrations^19,20^. Similar to the previous report, **6** and **7** could not bind or only marginally bound to PikC_WT_ so that their *K*_*D*_ values could not be determined due to poor binding and limited substrate solubility (Supplementary Table 1). By contrast, the *K*_*D*_ values of **6** and **7** towards PikC_H238*p*AcF_ were determined as 207.5 µM and 236.9 µM, respectively, comparable to those of **4** and **5** towards PikC_WT_ (177.6 µM and 373.6 µM). Compared with PikC_WT_, the binding affinities of PikC_H238F_ and PikC_H238Y_ to **4** increased by 14.9 and 9.6 times, respectively. The affinity of PikC_H238*p*AcF_ to **4** also increased by 3.4-fold (*K*_*D*_ = 40.2 µM). Similarly, the binding affinities of PikC_H238F_ and PikC_H238Y_ to **5** increased by 4.1 and 4.9 times, respectively. The affinity of PikC_H238*p*AcF_ to **5** also increased by 3.3-fold (*K*_*D*_ = 87.1 µM). All these results are well consistent with the corresponding enzymatic activities (Fig. 3), at least partially explaining the improved activities of the His238 mutants.

### Extended ncAA mutagenesis analysis

Motivated by the above results, we tested more aromatic ncAAs with diverse *para*-substituents for His238 replacement, including *p*-ethynyl-L-phenylalanine (*p*ENF)^29^, 4-phenyl-L-phenylalanine (BipA)^30^, *p*-propargyloxyphenylalanine (*p*PrF)^30^, *p*-azido-L-phenylalanine (*p*AzF)^30^, *O*-methyltyrosine (*O*MeY)^31^, *O*-*tert*-butyltyrosine (*Ot*BuY)^31^, and *p*-bromophenylalanine (*p*BrF)^31^ (Fig. 3b). All purified mutants (Supplementary Table 4) except PikC_H238BipA_ exhibited varied hydroxylation activities towards **6**, with PikC_H238*p*AcF_ giving the highest hydroxylation activity. Unlike **6** that could be accepted by almost all ncAA mutants to different extents, the 14-membered ring macrolactone **7** was only hydroxylated by PikC_H238*p*AcF_ (Fig. 3b). We reasoned that these activity profiles might result from the complicated interplay of substrate structures and the size, shape and electronic property of the ncAA side chain. Essentially, all the active ncAA mutants have an aromatic side chain larger than tyrosine (Supplementary Fig. 8).

### Crystallographic/mechanistic analyses of PikC_H238*p*AcF_

**Fig. 4:**
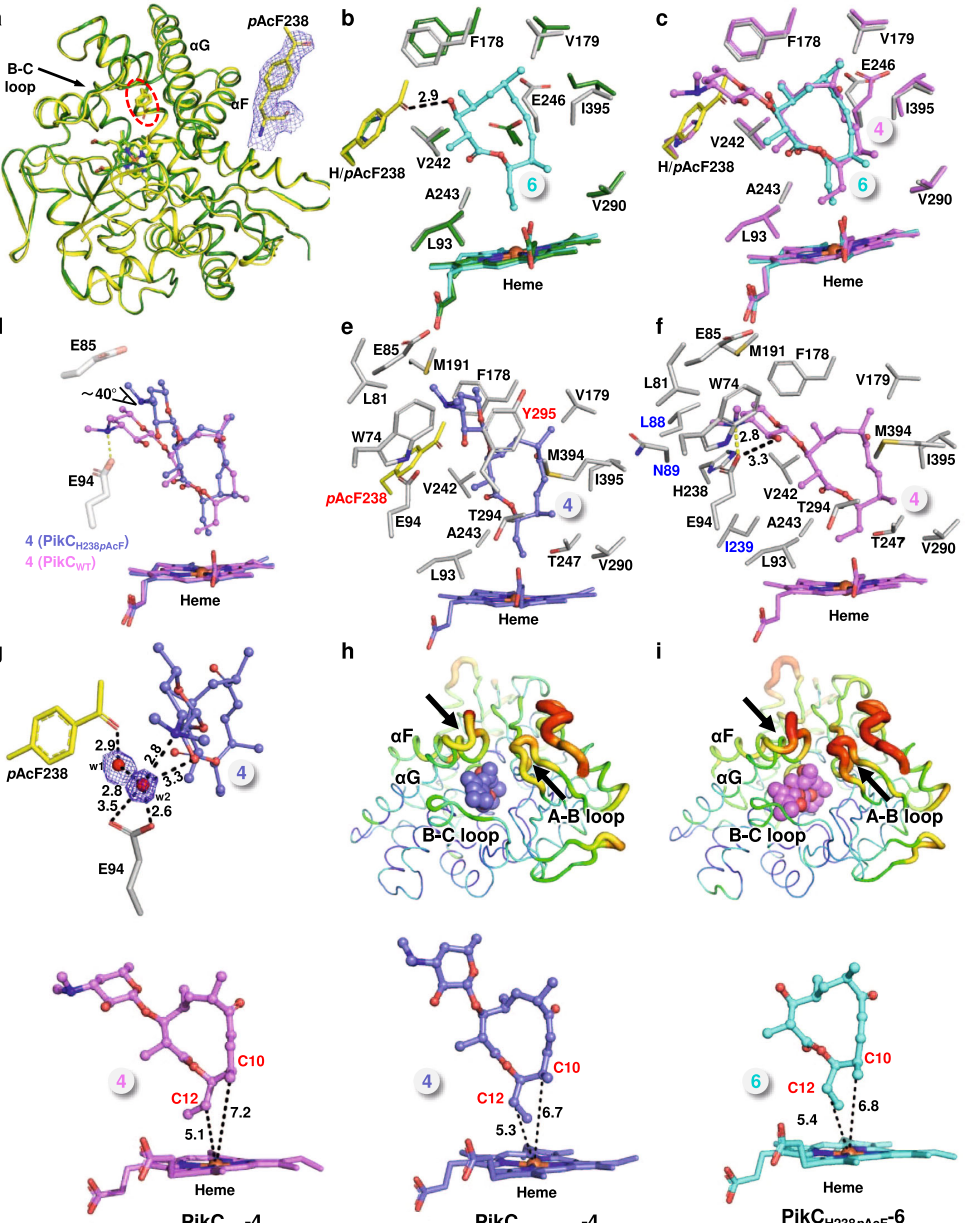
Structural analysis of substrate-free, 6-bound, and 4-bound PikC_H238*p*AcF_. **a** Superposition of substrate-free PikC_H238*p*AcF_ (yellow) with PikC_WT_ (PDB ID: 2BVJ; forest). The incorporated ncAA *p*AcF at H238 site is highlight as yellow stick, and its weighted 2*Fo*–*Fc* electron density map is contoured at the level of 1.5 σ (blue mesh). **b**, **c** Comparisons of the substrate binding site of **6**-bound PikC_H238*p*AcF_ with that of substrate free PikC_WT_ (**b**) and with that of **4**-bound PikC_WT_ (**c**). The side chains of interacting amino acids are shown as sticks and colored in grey (PikC_H238*p*AcF_), forest (substrate free PikC_WT_), and violet (**4**-bound PikC_WT_). The ncAA *p*AcF is highlighted in yellow. The substrates are shown in ball-stick and colored in cyan (**6**) and violet (**4**). The heme groups are colored consistent with the corresponding substrates. The hydrogen bond in angstrom is shown as dashed black line. **d** Schematic diagram of the positions of **4** in superimposed PikC_H238*p*AcF_ and PikC_WT_. The salt bridge formed between Glu94 and the C3’ dimethylamino group of the desosamine moiety in **4** is shown as yellow dashed line. **e**, **f** The substrate binding pockets formed by the amino acid side chains within 4.5 Å from **4** in the **4**-bound PikC_H238*p*AcF_ (**e**) and **4**-bound PikC_WT_ (**f**). The newly involved residues are labeled in red and the lost residues in blue. **g** Detailed view of the hydrogen bond network formed by *p*AcF238, E94 and **4** through two water molecules in **4**-bound PikC_H238*p*AcF_. The water molecules are shown as red spheres, and the weighted 2*Fo*–*Fc* electron density map is contoured at the level of 1.0 σ (blue mesh). The hydrogen bonds in angstrom are shown as dashed black lines. **h**, **i** Comparisons of the structural dynamics of **4**-bound PikC_H238*p*AcF_ (**h**) and **4**-bound PikC_WT_ (**i**) represented by B-factor values. Blue, green, yellow, orange, and red colors scale the structures from rigid to flexible. Areas with large variation are indicated by black arrows. **j** Orientations of **4** and **6** in PikC_WT_ and PikC_H238*p*AcF_. The distances from the C10 and C12 atoms to the heme-iron reactive center are labeled in angstrom.

To understand the molecular mechanism of the unique catalytic activity of PikC_H238*p*AcF_, we first determined the high-resolution three-dimensional structure of substrate-free PikC_H238*p*AcF_ (PDB ID code: 7XBM, 2.4 Å resolution, Supplementary Table 9). In each asymmetric unit, there are two molecules of PikC_H238*p*AcF_ (named Chain A and Chain B) adopting the almost identical conformation with a root mean square deviation (rmsd) of 0.171 Å over 334 equivalent Cα atoms (Supplementary Fig. 52). Clear electron density corresponding to the ncAA *p*AcF was unambiguously observed at the amino acid residue 238 site, indicating that the His238 of PikC_WT_ was replaced by *p*AcF with a very high incorporation efficiency (Fig. 4a), which is consistent with the results of ESI-Q-TOF-MS analysis (Fig. 2c) and tandem mass spectrometry (Supplementary Fig. 2). The overall structure of PikC_H238*p*AcF_ is virtually identical to the previously determined PikC_WT_ structure in a closed conformation (PDB ID code: 2BVJ, Chain A), with a rmsd of 0.323 Å over 345 Cα atoms, which indicates the incorporation of *p*AcF does not change the overall structure of PikC (Fig. 4a, Supplementary Fig. 52).

To further gain mechanistic insights into the unique substrate selectivity and hydroxylation pattern of PikC_H238*p*AcF_, we solved the crystal structures of **6**-bound and **4**-bound PikC_H238*p*AcF_ (Supplementary Fig. 53). Similar to the substrate free PikC_H238*p*AcF_ structure, in both structures, each crystallographic asymmetric unit has two protein chains (named Chain A and Chain B). There is basically no major conformational difference between Chains A and B (**6**-bound, rmsd = 0.169 Å; **4**-bound, rmsd = 0.155 Å), all of which are in a closed conformation (Supplementary Fig. 55, Supplementary Figs. 60a, b). Therefore, structures of one representative chain (Chain A) were selected for further analysis.

In the crystal structure of PikC_H238*p*AcF_ in complex with **6** (PDB ID code: 7XBO, 2.2 Å resolution, Supplementary Table 9), the 12-membered ring macrolactone is unambiguously positioned in the active site, and its electron density is well defined (Supplementary Fig. 53). Similar to the interactions between the macrolactone portion of **4** in PikC_WT_ (PDB ID code: 2C6H), **6** hydrophobically interacts with the side chains of Leu93, Phe178, Val179, Val242, Ala243, Val290, and Ile395 (Fig. 4b). Consistent with the observation in the **4**-bound PikC_WT_ structure, upon binding of the ligand **6**, the carboxyl group of Glu246 side chain is flipped away to better accommodate the macrolactone ring of **6** (Fig. 4b, c). Importantly, the carbonyl group of *p*AcF238 and the C3 hydroxyl group of **6** form a 2.9 Å hydrogen bond (Fig. 4b). Meanwhile, the terminal methyl group of *p*AcF238 hydrophobically contacts with the C3 carbon atom and C4 methyl group of **6**. Essentially, these extra interactions would not exist without the incorporation of *p*AcF into PikC at the position of His238 (Fig. 4b). Therefore, the extra strong hydrogen bond and additional hydrophobic interactions mediated by ncAA in PikC_H238*p*AcF_ simulate the interactions to some extent mediated by desosamine moiety of **4** in the **4**-bound PikC_WT_ structure (Fig. 4c) and likely play a vital role in the catalysis of PikC_H238*p*AcF_ towards the non-natural substrate **6**.

Compared with PikC_WT_, PikC_H238*p*AcF_ showed a higher catalytic activity for **4** (Fig. 3e). In the crystal structure of **4**-bound PikC_H238*p*AcF_ (PDB ID code: 7XBN, 2.0 Å resolution, Supplementary Table 9), clear electron density corresponding to the 12-membered ring macrolide **4** is unambiguously observed at the active site (Supplementary Fig. 53). Upon replacement of His238 by the bulkier *p*AcF, the desosamine sugar is pushed away and tilts 40 degrees, comparing to the conformation of **4** in the substrate binding pocket of PikC_WT_, in the **4**-bound PikC_H238*p*AcF_ structure. This sugar relocation leads to the loss of the key salt bridge with Glu94 as observed in the **4**-bound PikC_WT_ structure, thereby leading **4** to be more perpendicular to the heme plane (Fig. 4d). The majority of hydrophobic interactions between **4** and PikC are conserved in the **4**-bound PikC_WT_ and PikC_H238*p*AcF_ structures, such as the amino acids Trp74, Leu81, Glu85, Met191, and Phe178 to the desosamine moiety, and Leu93, Phe178, Val179, Val242, Ala243, Thr247, Val290, Thr294, Met394, and Ile395 to the macrolactone ring of **4**. Due to the relative position change, **4** loses several hydrophobic contacts with PikC_H238*p*AcF_ via Leu88, Gln89, and Ile239 (Fig. 4e, f). Although PikC_H238*p*AcF_ loses some electrostatic and hydrophobic interactions with **4**, the presence of *p*AcF238 also introduces several extra interactions for **4**. Specifically, the carbonyl group of *p*AcF and the carboxyl group of Glu94 form a hydrogen bond network with the C3’ hydroxyl group of desosamine through two water molecules (Fig. 4g), and the methyl group of *p*AcF238 forms hydrophobic interactions with the C4 and C5’ methyl groups of **4** (Fig. 4e). In addition, the specific position of **4** in PikC_H238*p*AcF_ also introduces the hydrophobic interaction between desosamine and Tyr295 (Fig. 4e). All these extra interactions likely contribute to the increased binding affinity of PikC_H238*p*AcF_ towards **4**. Further structural analysis revealed that in the **4**-bound PikC_H238*p*AcF_ structure, the loop between F and G helices and the A-B loop become less flexible than in the **4**-bound PikC_WT_ structure (Fig. 4h, i). We reason that the positive charge of the protonated C3’ dimethylamino group of desosamine in new position might better interact with the negative charges of the loop between F and G helices (Asp182-Asp183) and the A-B loop (Glu48-Gly49-Asp50-Glu51) in the **4**-bound PikC_H238*p*AcF_ structure. These structural features demonstrate a “tighter” binding mode of **4** in the productive substrate binding pocket of PikC_H238*p*AcF_, by which the binding affinity and activity of PikC_H238*p*AcF_ towards **4** are significantly increased.

**Fig. 5:**
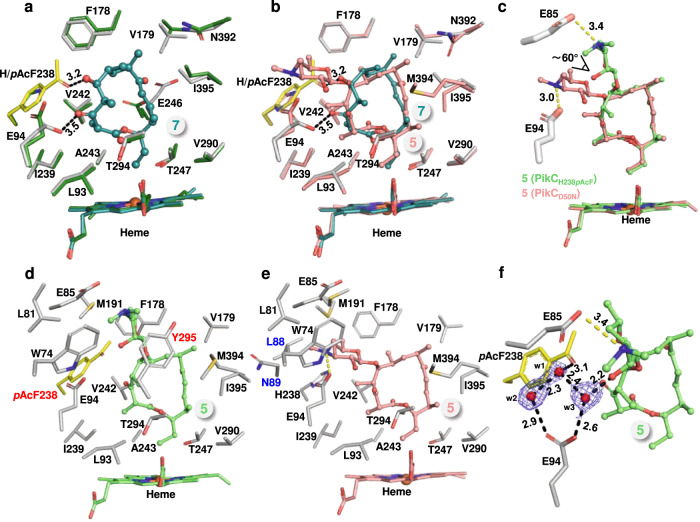
Structural analysis of narbonolide (7)-bound and narbomycin (5)-bound PikC_H238*p*AcF_. **a**, **b** Molecular modeling of **7** within the substrate binding pocket of PikC_H238*p*AcF_: comparisons of contacting amino acid side chains around the substrate binding site within 4.5 Å between **7**-bound PikC_H238*p*AcF_ and substrate-free PikC_WT_ (**a**) and **5**-bound PikC_D50N_ (**b**). The side chains are shown as sticks in grey (PikC_H238*p*AcF_), forest (substrate-free PikC_WT_), and salmon (**5**-bound PikC_D50N_). The heme groups are colored consistent with the corresponding substrates. The hydrogen bonds are shown as dashed black line. **c** Schematic diagram of the positions of **5** in superimposed PikC_H238*p*AcF_ and PikC_D50N_. The salt bridges formed between the acidic amino acid residues and the C3’ dimethylamino group of the desosamine moiety in **5** are shown as yellow dashed lines. **d**, **e** The substrate binding pockets formed by the amino acid side chains within 4.5 Å from **5** in the **5**-bound PikC_H238*p*AcF_ (**d**) and **5**-bound PikC_D50N_ (**e**). The newly involved residues are labeled in red and the missing residues in blue. **f** Detailed view of the hydrogen bond network formed by *p*AcF238, E94 and **5** through three water molecules in **5**-bound PikC_H238*p*AcF_. The water molecules are shown as red spheres, and the weighted 2*Fo*–*Fc* electron density map is contoured at the level of 1.0 σ (blue mesh). The hydrogen bonds in angstrom are shown as dashed black lines.

Due to continuous decomposition of **7** in aqueous solution and lower binding affinity than **6**, we were unable to solve the crystal structure of **7**-bound PikC_H238*p*AcF_. Thus, molecular docking with AutoDock vina was performed to explore the binding mode of **7** in PikC_H238*p*AcF_. According to the structural superimpositions of the **7**-PikC_H238*p*AcF_ docking model with the substrate free PikC_WT_ (Fig. 5a) and with the previously determined structure of a PikC mutant in complex with **5** (i.e., the more active PikC_D50N_ with a productive substrate binding conformation) (Fig. 5b), the 14-membered ring macrolactone is stabilized mainly through hydrophobic interactions between the macrolactone and the amino acids Leu93, Phe178, Val179, Ile239, Val242, Ala243, Thr247, Thr294, and Ile395. Importantly, the carbonyl group of *p*AcF238 and the C5 hydroxyl group of **7** form a 3.2 Å hydrogen bond. In addition, the C3 carbonyl group of **7** form a 3.5 Å hydrogen bond with the carboxy group of Glu94 (Fig. 5a). Taken together, we reason that the hydrophobic interactions and two extra hydrogen bonds likely enable the activity of PikC_H238*p*AcF_ towards the non-natural substrate **7**. Of note, the crystal structure of PikC_D50N_/**5** showed the productive binding pose of the 14-membered ring substrate (PDB ID code: 2VZM, Chain B) rather than the non-productive binding mode seen in the PikC_WT_/**5** complex structure (PDB ID code: 2C7X); therefore, we elected to use the PikC_D50N_/**5** structure as a reference for the following analyses. Since the structures of PikC_D50N_/**5** and PikC_WT_/**5** are virtually identical to each other except for the different 50th residues and alternative substrate binding conformations (Supplementary Fig. 54), we deemed that the comparisons using PikC_D50N_/**5** should be valid.

PikC_H238*p*AcF_ also exhibited higher hydroxylation activity towards the 14-membered ring macrolide **5** than PikC_WT_ (Fig. 3f). To elucidate the molecular basis for this observation, we further determined the crystal structure of PikC_H238*p*AcF_ in complex with **5** in a closed conformation (PDB ID code: 8GUE, 1.9 Å resolution, Supplementary Table 9, Supplementary Figs. 55 and 60c). In the complex structure, clear electron density corresponding to the structure of **5** was unambiguously observed at the active site (Supplementary Fig. 53). The H238*p*AcF mutation pushes away the desosamine for about 60 degrees in the structure of **5**-bound PikC_H238*p*AcF_. This substrate repositioning abolishes the salt bridge between desosamine and Glu94 as observed in the PikC_WT_/**5** complex, and rebuilds a salt bridge between desosamine and Glu85 (Fig. 5c). Consistent with the substrate-enzyme interactions between **5** and PikC_WT_, **5** in PikC_H238*p*AcF_ is also stabilized through a number of hydrophobic interactions with the amino acids Leu81, Leu93, Phe178, Val179, Ile239, Val242, Ala243, Thr247, Val290, Thr294, Met394, Ile395, Trp74, and Met191. Due to the relative position change, **5** loses several hydrophobic contacts with Leu88 and Gln89. Nevertheless, there in PikC_H238*p*AcF_/**5** exists an extra hydrophobic contact between the desosamine moiety and Tyr295. Importantly, in the PikC_H238*p*AcF_ structure in complex with **5**, *p*AcF238 introduces more hydrophobic interactions through the methyl group of this ncAA with the C5’ methyl group of the desosamine moiety and the C6 methyl group of the macrolactone ring (Fig. 5d, e). Moreover, three water molecules were found to mediate a hydrogen bond network between the *p*AcF carbonyl, Glu94 carboxyl and C3’ hydroxyl group of desosamine in the co-crystal structure (Fig. 5f). Therefore, the hydrophobic interactions mediated by *p*AcF238, Tyr295 and the water-mediated hydrogen bond network likely make the main contribution to the increased binding affinity of PikC_H238*p*AcF_ towards **5**.

### Probing the structural basis for the regioselectivity of PikC_H238*p*AcF_

**Fig. 6:**
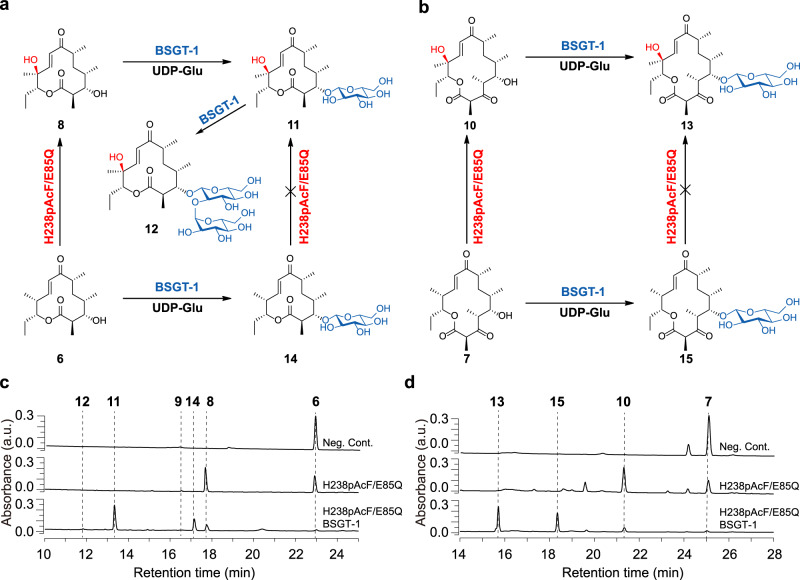
The artificial enzyme cascade with an oxidation-glycosylation order. **a**, **b** Schemes of the PikC_H238*p*AcF/E85Q_-BSGT-1 enzyme cascade with **6** (**a**) and **7** (**b**) as substrates. The functional groups introduced by P450 enzyme and glycosyltransferase are highlighted in red and blue, respectively. **c**, **d** HPLC analysis (230 nm) of the enzymatic reactions using **6** (**a**) and **7** (**b**) as substrates.

In addition to the broadened substrate scope and increased catalytic activity, PikC_H238*p*AcF_ also exhibits significantly improved regioselectivity for the C10 hydroxylation of both **4** (Fig. 3e) and **6** (Fig. 3c). As previously observed^19^, in the PikC_WT_/**4** complex, the allylic C10 and methylene C12 positions are 7.2 Å and 5.1 Å away from the heme-iron reactive center, respectively (Fig. 4j, Supplementary Fig. 61a). This observation did not directly explain the equal distribution of the two hydroxylation products **1** and **2** because the hydroxylation pattern could be determined by multiple factors, such as the substrate repositioning resulted from the catalytic oxygen bonding and/or interaction with redox partner protein, differential bond dissociation energy of the C-H bonds to be oxidized, and protein dynamics that cannot be observed in crystal structures. Nonetheless, a correlation between the C10- and C12-to-iron distances and the hydroxylation product ratio could still be established. Upon examination of the complex structures of PikC_H238*p*AcF_/**4** and PikC_H238*p*AcF_/**6**, we found that the distances between the C10 atom (of **4** and **6**) and the heme-iron reactive center are significantly shortened to 6.7 and 6.8 Å, respectively, when compared to the corresponding distance of 7.2 Å observed in the PikC_WT_/**4** complex structure; while the corresponding C12-to-iron distances are 5.3 and 5.4 Å (for **4** and **6**, respectively), longer than the corresponding distance of 5.1 Å in the PikC_WT_/**4** complex structure (Fig. 4j, Supplementary Figs. 61a–c). These distance changes might reflect the disruption of the original balance between the two hydroxylation sites of C10 and C12, thus leading to the shifted regio-preference to C10 position.

Unlike the different regioselectivity of PikC_H238*p*AcF_ and PikC_WT_ towards **4** and **6**, PikC_H238*p*AcF_ displayed similar regioselectivity to **5** and **7** as PikC_WT_ and the more active mutant of PikC_D50N_^20^. Comparatively, the distances between the C12 atom and the heme-iron reactive center are 7.4 Å in both complex structures of PikC_H238*p*AcF_/**5** and PikC_D50N_/**5** (reflecting the productive binding of **5** to PikC_WT_^20^ as described above); while the corresponding C14-to-iron distances are 5.3 Å (PikC_H238*p*AcF_/**5**) and 5.5 Å (PikC_D50N_/**5**), respectively, basically remains unchanged (Supplementary Fig. 56, Supplementary Figs. 61d, e). Biochemically, the farther (relative to heme-iron) C12 C-H bond instead of the closer C14 C-H bond is the preferred hydroxylation site perhaps due to the lower bond dissociation energy of the C12 allylic C-H bond and the unfavorable orientation of the C14 C-H bond^19,20^. The docking analysis of PikC_H238*p*AcF_/**7** demonstrates a similar C12-to-iron distance (7.3 Å) as observed in both complex structures (Supplementary Fig. 56). These observations may explain the same regioselectivity of PikC_H238*p*AcF_ and PikC_WT_ towards **5** and **7**.

### Engineering of a strict aglycone hydroxylase

Next, we attempted to engineer PikC_H238*p*AcF_ to be a strict hydroxylase of **6** and **7** by aborting the activity towards both **4** and **5**. Of note, a unique substrate anchoring mechanism of PikC has been unravelled by Sherman and co-workers^19–21^. Briefly, the acidic side chain of Glu94 is responsible for forming a salt bridge with the positively charged C3’-dimethylamino group of desosamine in **4** and **5**. Glu85, another acidic residue to sandwich the C3’-dimethylamino group together with Glu94, is also critical for both **4**- and **5**-hydroxylating activities and shows some synergistic effect with Glu94^19,20^.

Thus, we elected to mutate Glu94 and/or Glu85 to either glutamine or alanine in combination with the H238*p*AcF mutation. Consistent with the previous reports^19,20^, the substrate conversion ratios of the four single mutants including PikC_E85Q_, PikC_E85A_, PikC_E94Q_, and PikC_E94A_ towards **4**/**5** were reduced from 45.0 ± 4.5%/54.4 ± 2.8% (PikC_WT_) to 38.5 ± 3.1%/27.5 ± 4.4%, 43.3 ± 3.8%/25.8 ± 1.9%, 14.2 ± 4.7%/18.6 ± 4.6%, and 8.2 ± 2.7%/34.0 ± 4.8%, respectively; while the four double mutants PikC_E85A/E94A_, PikC_E85A/E94Q_, PikC_E85Q/E94A_, and PikC_E85Q/E94Q_ almost entirely lost their activities. Unsurprisingly, all these single/double mutants were inactive to **6** and **7** (Supplementary Fig. 57). When the PikC_H238*p*AcF_ mutation was introduced into these less active mutants, all double and triple mutants displayed the C10-hydroxylation activity towards **6** and C12-hydroxylation activity towards **7** (Fig. 3c, d, Supplementary Fig. 57). In particular, the triple mutant PikC_H238*p*AcF/E85Q/E94Q_ almost entirely lost the activity towards both **4** and **5**, but showed 12.0 ± 0.5% and 13.4 ± 2.4% conversion ratios for **6** and **7**, respectively. Interestingly, compared with PikC_H238*p*AcF_, the double mutant PikC_H238*p*AcF/E85Q_ exhibited 0.4- and 0.5-fold increase in the hydroxylation activity towards **6** and **7** (Fig. 3c, d) for unknown reasons. However, it retained significant activities towards **4** (52.2 ± 2.8% conversion) and **5** (94.3 ± 3.1% conversion) (Fig. 3e, f). Consistently, the binding affinities of PikC_H238*p*AcF/E85Q_ to **6** and **7** increased by 0.3 and 0.4 times, respectively, compared to those of PikC_H238*p*AcF_ (Supplementary Table 1).

### Establishing an artificial enzyme cascade for unnatural macrolides

With the PikC_H238*p*AcF/E85Q_ mutant, we sought to establish an artificial enzyme cascade in vitro with an oxidation-glycosylation order (Fig. 6a, b), which would reverse the natural glycosylation-oxidation order in the late pikromycin biosynthetic pathway, which is sequentially mediated by the glycosyltransferase DesVII and P450 PikC (Fig. 1a). First, the in vitro hydroxylation reactions of **6** and **7** by PikC_H238*p*AcF/E85Q_ were optimized by adding glucose-6-phosphate dehydrogenase (GDH) and glucose for NADPH regeneration and extending the reaction time from 40 min to 4 h. As a result, the conversions of **6** and **7** achieved 75.3% and 78.0%, respectively (Fig. 6c, d). Second, we tested the glycosylation activities of six UDP-dependent glycosyltransferases towards the hydroxylated macrolactones **8** and **10** by using UDP-glucose as the sugar donor, including UGT85A1^32^, RrUGT17^33^, RrUGT3^33^, UGT72B14^34^, UGT73B6FS and BSGT-1^35,36^. BSGT-1 turned out to be the only active enzyme, glucosylating **8** and **10** into methynolide-3-*O*-glucoside (**11**), O3,O2’-di-glucosyl-methynolide (**12**) and pikronolide-5-*O*-glucoside (**13**) (Fig. 6a, b; see Supplementary Figs. 23–39 for structural determination). Due to the incomplete oxidation of **6** and **7**, the unreacted **6** and **7** were also glucosylated by BSGT-1, demonstrating the extraordinary substrate promiscuity of this UGT. Unsurprisingly, the resulting products **14** and **15** (i.e., 10-deoxymethynolide-3-*O*-glucoside and narbonolide-5-*O*-glucoside, see Supplementary Figs. 40–50 for structural determination) cannot be recognized by PikC_H238*p*AcF/E85Q_ (Supplementary Fig. 58).

## Discussion

Selective C-H bond oxidation remains a central challenge in synthetic chemistry^21,37^. To address this issue, tremendous efforts on enzyme engineering of cytochrome P450 enzymes have been made in order to improve the activity, substrate scope, stability, robustness, and other catalytic properties of these selective biocatalysts for practical bio-oxidation^7,38–47^. To engineer the P450 mutants with desired properties, a variety of effective mutagenesis strategies such as CAST, CAST/ISM, and FRISM have been developed with great successes achieved^48–50^. However, the 20 proteinogenic amino acids cannot always satisfy the requirement of site-directed and/or random mutagenesis and rational protein design in terms of steric, topological and electronic properties or other inspiring reactivity. Therefore, appropriate ribosomal incorporation of ncAAs in a site-specific manner, which could be designed on demand theoretically, has shown significant possibilities to overcome the limit of conventional protein mutagenesis^51–54^.

Compared with extensive studies on ncAA-based protein engineering for protein labeling, crosslinking, photo-activation, bioorthogonal conjugation and other purposes^52^, the use of ncAA mutagenesis to tailor the catalytic activities of diverse enzymes, especially P450 enzymes, remains largely underexplored. Among few examples, NMR-active ncAAs were incorporated into P450 enzymes to illuminate the role of conformational dynamics^55^. Recently, Kolev et al. utilized four ncAAs to scan the 11 active-site positions of CYP102A1, leading to unusual changes in regioselectivity and catalytic efficiency^25^. This pioneering and inspiring exploration represents the first semi-rational engineering of P450 active site using ncAAs in a site-specific manner.

Aiming to change the substrate specificity of a biosynthetic P450 enzyme (PikC) from the sugar-appended macrolides (**4** and **5**) to the corresponding aglycones (**6** and **7**), in this study, we successfully engineered a number of ncAA-containing PikC mutants that are capable of selectively hydroxylating **6** at C10 position. PikC_H238*p*AcF_, as the most efficient C10-hydroxylase of **6**, was also the only mutant enzyme to catalyze the C12-hydroxylation of **7** giving rise to **10**. Further structural analysis provided mechanistic basis for the ability of PikC_H238*p*AcF_ to selectively catalyze the hydroxylation of **6** and **7**. We envision that our functional, crystallographic, and mechanistic study will advance the engineering of cytochrome P450 enzymes using ncAAs.

The relevant mechanisms were elucidated by comparative analysis of four crystal structures of PikC_H238*p*AcF_. As the best ncAA substituent, *p*AcF creates a specific binding strategy for **6** and **7**. In PikC_WT_, the desosamine sugar acts as a substrate anchor by forming a key salt bridge between the dimethylamino group and the acidic amino acid residue and a network of hydrogen bonds with the native substrate **4** or **5**^19,20^. In PikC_H238*p*AcF_, the benzylic carbonyl group and terminal methyl groups respectively provide the hydrogen bond and hydrophobic contacts with the macrolactone ring of **6** or **7** without the desosamine anchor (Figs. 4 and 5). In this scenario, the methyl keto moiety of *p*AcF, which cannot be provided by any proteinogenic amino acids, is essential for productive binding of the two aglycones.

In natural product biosynthetic pathways, the order of enzymatic steps is usually fixed to prevent metabolic flux leaking. However, a locked pathway would limit pathway reprogramming for structural diversification. Using the ncAA-engineered macrolactone hydroxylase PikC_H238*p*AcF_, we successfully reversed the natural glycosylation-oxidation order of late pikromycin biosynthetic pathway, by which it becomes feasible to attach different sugars to the aforehand oxidized macrolactone (Fig. 6). Otherwise, the GT-mediated attachment of a non-desosamine sugar to **6** or **7**, which was previously achieved by Borisova et al.^56^, would disable PikC since desosamine (or a dimethylamino group) is required for PikC’s activity^19,20^. Thus, we envision that the substrate flexibility of GTs could be better harnessed for generation of more macrolides through changing the macrolide-oxidizing P450s into macrolactone-tailoring P450s by similar ncAA mutagenesis approaches in the future. Since glycosylation and oxidation can improve the properties of a compound in terms of aqueous solubility, biological activity, bioavailability and hence druggability^56^, a better coordinated biosynthetic glycosylation and oxidation that is enabled by ncAA mutagenesis would likely benefit the future drug development.

In conclusion, herein we practiced the semi-rational ncAA mutagenesis for a natural product biosynthetic P450 enzyme. We conceived and realized a unique binding strategy for two unnatural substrates in PikC based on their structural characteristics, which also improved the catalytic performance towards natural substrates. With the unnatural macrolactone hydroxylating activities, PikC_H238*p*AcF_ was employed to rewire the pikromycin biosynthetic pathway in vitro. The strategy of ncAA-based P450 enzyme engineering and the mechanisms gained from detailed structural analysis of enzyme-substrate complexes could be applied in more glycosylation-oxidation coupled systems in the future. Furthermore, this kind of biosynthetic pathway reprogramming may also be performed in vivo given that the required ncAA can be supplied by feeding or in situ biosynthesis^57^. With the useful toolkit of ncAAs, we anticipate that more fine-tuning enzymatic reactions or cascades will be created and more rationally designed natural product derivatives could be produced.

## Methods

### Materials

Unless otherwise specified, all ncAAs and antibiotics used in this study were purchased from Suzhou Amatek Biotechnology and Sigma Aldrich, respectively. All P450 enzymatic reactions were supported by NADPH from Qingdao Baisai Biotechnology. *δ*-Aminolevulinic acid (5-ALA) and isopropyl-*β*-D-thiogalactopyranoside (IPTG) were bought from Shanghai Aladdin Biochemical Technology. Taq DNA Polymerase and Agarose Gel DNA Recovery kit were purchased from Beijing Dingguo Changsheng Biotechnology. All plasmids were prepared in *E. coli* DH5α using MonPure™ Plasmid Mini-Prep Kit from Qingdao Baisai Biotechnology. MultiF Seamless Assembly Mix was purchased from Abclonal. All proteins were purified using Ni-NTA Sefinose^TM^ Resin (Settled Resin) from Sangon Biotech. Iodoacetamide (IAA), dithiothreitol (DTT), and trypsin were obtained from Sigma Aldrich. C18 ZipTip was purchased from Millipore. Polypeptone and uridine 5’-diphosphoglucose (UDP-Glu, disodium salt) were bought from Beijing Solarbio Science & Technology. Primer synthesis and DNA sequencing were performed by Sangon Biotech.

### Molecular cloning

The PikC expression vector pET28a-*pikC*^19^ and plasmid pULTRA-Ambrx (gifted by Professor Xiaozhou Luo at Shenzhen Institute of Advanced Technology, Chinese Academy of Sciences) bearing the *Mj*TyrRS/tRNA_CUA_ orthogonal system (pUltra-AcrFRS/tRNA^Tyr^_CUA_)^58^ were used for preparation of the ncAA-containing mutants of PikC. Briefly, pULTRA-Ambrx contains an amber suppressor *M. jannaschii* tRNA^Tyr^_CUA_ (*Mj* tRNA^Tyr^_CUA_) and encodes a cognate tRNA synthetase mutant *Mj*TyrRS carrying seven active-site mutations including Y32V, L65Y, F108H, Q109G, D158G, L162E, and D286R. The plasmid also contains the kanamycin resistance gene and regulatory gene *lacI* to encode the repressor of lactose operon. Incorporation of the amber codon into different positions of PikC was achieved by PCR reactions using pET28a-*pikC* as a template and the specific mutagenesis primers as listed in Supplementary Table 3. The plasmids pET28a-*pikC* with the amber codon incorporated and pULTRA-Ambrx were co-transformed into *E. coli* BL21(DE3) by electroporation. All constructed plasmids were verified by DNA sequencing.

### Protein expression and purification

PikC_WT_ and mutants were overexpressed in *E. coli* BL21(DE3) cells. A colony of each individual protein expression strains was inoculated into LB broth containing kanamycin (50 µg/mL) and spectinomycin (50 µg/mL) and grown at 37 °C and 220 rpm for 12 h. This culture was inoculated into 0.5 L TB medium containing kanamycin (50 µg/mL) and spectinomycin (50 µg/mL) at a ratio of 1:100 (*v*/*v*) in a 2 L conical flask, which was grown at 37 °C and 200 rpm for 4~6 h. When OD_600_ reached 0.6~0.7, a certain ncAA was added to the final concentration of 2 mM. When OD_600_ reached 0.8~0.9, IPTG was added to the final concentration of 0.5 mM to induce the expression of both P450 enzyme and orthogonal tRNA synthetase at 18 °C and 160 rpm for 18~20 h. Cells were harvested by centrifugation (6000 × *g*, 15 min). The target proteins were purified according to the procedure established previously^17^. Briefly, cells resuspended in lysis buffer (50 mM NaH_2_PO_4_, 300 mM NaCl, 10 mM imidazole, 10% glycerol, pH 8.0) were crushed by high-pressure homogenizor (ATS, Shanghai, China) at 4 °C. Cell lysates were prepared by high-speed centrifugation (10,000 × *g*, 60 min, 4 °C), to which Ni-NTA agarose was added and followed by gently mixing at 4 °C for 1 h. Then, the Ni-NTA agarose was loaded onto a gravity column and the impurity proteins were washed out with washing buffer (50 mM NaH_2_PO_4_, 300 mM NaCl, 20 mM imidazole, 10% glycerol, pH 8.0). The target proteins were eluted with elution buffer (50 mM NaH_2_PO_4_, 300 mM NaCl, 250 mM imidazole, 10% glycerol, pH 8.0). Finally, a suitable Amicon size-exclusion column was used for protein concentration, and a PD-10 desalting column was employed for buffer exchange to storage buffer (0.2 mM dithioerythritol, 1 mM EDTA, 50 mM NaH_2_PO_4_, 10% glycerol, pH 7.3). The purified proteins were flash frozen by liquid nitrogen and stored at −80 °C for later use. P450 concentrations were determined from the CO-reduced difference spectra using the extinction coefficient of 91,000 M^−1^ cm^−1^ (ref. ^20^).

### Construction of the gene knockout plasmids

All primers used to construct the knockout plasmids are listed in Supplementary Table 2. The knockout plasmids were derived from the modification of the CRISPR/Cas9 gene editing plasmid pKCcas9dO^59^. To generate the *pikC* (5576176–5577426 bp) and *desVII* (5587287–5588567 bp) deletion plasmids, the sgRNA expressing cassettes were amplified with the primer pairs of PikCsgRNA-F/PikCsgRNA-R and DesVIIsgRNA-F/DesVIIsgRNA-R. The two homologous arms flanking *pikC* and *desVII* were amplified from *S. venezuelae* genomic DNA separately with the primer pairs of PikC-up-F/PikC-up-R and PikC-dn-F/PikC-dn-R, and DesVII-up-F/DesVII-up-R and DesVII-dn-F/DesVII-dn-R. These DNA fragments were individually ligated into the *Spe*I/*Hind*III linearized pKCcas9dO vector using MultiF Seamless Assembly Mix. Correct constructs were confirmed by DNA sequencing, and subsequently used for transforming *S. venezuelae* strains.

### Preparation of substrates

PikC’s natural substrates **4** and **5** were prepared using the *pikC* knockout strain of *S. venezuelae* ATCC 15439 (*Sv*-*ΔpikC*). Aglycones **6** and **7** were obtained from the fermentation extract of the *desVII* knockout strain of *S. venezuelae* ATCC 15439 (*Sv*-*ΔdesVII*). As described above, the two gene deletion strains were generated using CRISPR-Cas9 gene editing technology^60^. Briefly, the *E. coli* ET12567/pUZ8002 strain harboring the knockout plasmid was mixed with *S. venezuelae* ATCC15439 in equal volume. The mixed bacterial solution was spread on the surface of MS agar containing 50 mM MgCl_2_ and 50 mM CaCl_2_. After incubation at 30 °C for 14–16 h, the MS plates were covered with 25 µg/mL nalidixic acid and 25 µg/mL apramycin, and further incubated until exconjugants appeared. To prepare the substrates, each strain was cultured at 30 °C and 220 rpm for 3 days. Then the seed culture was inoculated to 3 L of fermentation medium at a ratio of 1:10 (*v*/*v*). The fermentation was carried out at 30 °C and 220 rpm for 7 days (*note*: the acquisition of **7** only took two days of fermentation). Afterwards, the fermentation broth (with mycelia removed by centrifugation) was extracted twice with 2× volumes of ethyl acetate. The organic extracts were dried with a rotary evaporator and re-dissolved in methanol. All substrates were isolated and purified using semi-preparative HPLC. The composition of the fermentation medium is as follow: 10.0 g glucose, 10.0 g glycerol, 10.0 g polypeptone, 5.0 g meat extracts, 5.0 g NaCl, and 2.0 g CaCl_2_•H_2_O in 1 L tap water; pH was adjusted to 7.3 before autoclaving. The addition of 0.06 M sodium acetate led to significant increases in the macrolide antibiotics production^61^.

### Substrate binding assay

Purified P450 enzymes were diluted to 1 µM with the storage buffer, which was titrated with the substrate dissolved in DMSO (20 mM) in 1 µL aliquots. The difference spectra were recorded at room temperature on a Molecular Devices Spectra Max M2 spectrometer in the wavelength range of 350–500 nm. The difference absorbance *∆A* (*A*_peak 390 nm_ − *A*_trough 420 nm_) calculated from at least duplicated data was plotted *versus* the substrate concentrations. Then, the data points were fit with the hyperbolic function *∆A* = *A*_max_ [*S*] / (*K*_*D*_ + [*S*]), where *A*_max_ is the maximum absorbance shift at saturation, [*S*] is the total concentration of substrate, and *K*_*D*_ is the apparent dissociation constant for the enzyme-substrate complex^20^.

### P450 enzymatic assays

The analytical scale P450 reactions were carried out with a volume of 100 μL in 1.5 mL centrifugation tubes, while the preparative scale reactions were conducted with a volume of 10 mL in 50 mL conical flasks. The analytical scale reaction containing 1 µM PikC (wild type or mutant), 1 mM NADPH, 10 µM Fdx1499, 5 µM FdR0978^14,62,63^, and 0.5 mM substrate in 100 μL storage buffer was incubated at 30 °C for 40 min. To quench the reaction, 150 μL methanol was added into the reaction mixture. After thorough vortex mixing and high-speed centrifugation (12,000 × *g*, 15 min) to remove cell debris and precipitated proteins, the supernatant was subjected to HPLC analysis. The reaction samples were analyzed on an Agilent 1220 HPLC system under a linear gradient mobile phase at 230 nm. The mobile phase system consisted of solvent A (water with 0.1% trifluoroacetic acid, TFA) and solvent B (acetonitrile, ACN). The following gradient program was applied: 0–3 min, 10% solvent B; 3–30 min, 10~90% solvent B; and 30–31 min, 90~100% solvent B, at a flow rate of 1.0 mL/min. Boiled enzymes were used in all negative control experiments. The preparative scale reaction system containing 5 µM PikC_H238*p*AcF/E85Q_, 1 U glucose-6-phosphate dehydrogenase (GDH), 1 mM glucose, 1 mM NADPH, 10 µM Fdx1499, 5 µM FdR 0978, and 0.5 mM substrate in 10 mL storage buffer was incubated at 30 °C for 4 h. Three times extraction with an equal volume of ethyl acetate was performed to extract enzymatic products. The organic extracts were dried *in vacuo* and re-dissolved in methanol for products purification by semi-preparative HPLC. To determine the structures of substrates and products by NMR, 2.0 mg **6**, 2.0 mg **7**, 1.2 mg **8**, 1.0 mg **9**, 2.3 mg **11**, 1.5 mg **12**, 2.2 mg **13**, 2.4 mg **14**, and 1.8 mg **15** were prepared in >95% purity.

### In vitro glycosylation reactions by BSGT-1

Expression and purification of glycosyltransferases (*e.g*., BSGT-1) basically followed the same protocol used for P450 enzymes with some slight modifications. In the end of the P450 reaction mediated by PikC_H238*p*AcF/E85Q_, 10 mM MgCl_2_, 3 mM UDP-Glu, and 9 µM BSGT-1 were added. The glycosylation reactions were performed at 37 °C for 2 h.

### In gel digestion and LC-MS/MS analysis

The target proteins were analysed on SDS-PAGE gels, and the gel spots (2 × 2 mm) containing the interested protein band were cut from the gel. The gel spots were cleaned with 0.5 mL Buffer I (25 mM NH_4_HCO_3_, 50% ACN) until becoming colorless, and vacuum-centrifuged to dryness. Then, 0.1 mL of 10 mM DTT was added to break disulfide bonds at 56 °C for 1 h before colloidal particles were cleaned by ACN. To alkylate histidine and cysteine, 100 µL of 55 mM IAA was added at room temperature in the dark, and excess liquid was removed after 30 min. Then, the colloidal particle was washed twice by 25 mM NH_4_HCO_3_ and ACN respectively before drying. The dehydrated colloidal particles were digested by trypsin at a ratio of 1:20 (*w*/*w*, trypsin:protein) at 37 °C for 12 h. For analysis by Q Exactive mass, 20 µL of 0.1% TFA was added to terminate the reaction, and peptides were dissolved after desalination with C18 ZipTip. Samples were eluted on an Ultimate 3000 HPLC (Dionex). The mobile phase system of solvent A (water with 0.1% formic acid) and solvent B (ACN with 0.1% formic acid) was applied at a flow rate of 0.4 µL/min. The gradient program was as follow: 0–6 min, 5~8% solvent B; 6–40 min, 8~30% solvent B; 40–45 min, 30~60% solvent B; 45–48 min, 60~80% solvent B; and 48–60 min, 80% solvent B.

### Database search

Proteome Discoverer (Thermo, version 1.4) was used for database search. The establishment of a reference database was achieved using the PikC amino acid sequence. The search parameters for PikC_H238*p*AcF_ were set as follows: (1) The molecular weight of His238 (155.07 Da) was replaced by *p*AcF (207.09 Da); (2) Trypsin was selected as the digestive enzyme; and (3) The alkylation of cysteine was set as a fixed modification and the oxidation of methionine as a variable modification.

### Crystallization and structure determination

The crystals of substrate-free PikC_H238*p*AcF_ were grown by hanging-drop vapor diffusion at 16 °C, by mixing 1 µL of 6 mg/mL of protein and 1 µL of reservoir solution (0.2 M Li_2_SO_4_, 0.1 M sodium cacodylate pH 6.1, 16% PEG8000) according to the previous report^21^. The **6**- and **4**-bound PikC_H238*p*AcF_ complexes were prepared by mixing 6 mg/mL PikC_H238*p*AcF_ with 5 mM **6** or **4** at 25 °C for 30 min, respectively. Initial crystallization conditions of the **6**- and **4**-bound PikC_H238*p*AcF_ complexes were screened by the sitting-drop vapor-diffusion method using commercial crystal screening kits at 16 °C. Optimization of conditions was carried out manually in 24-well plates by the hanging-drop vapor diffusion method. Crystals of **6**-bound PikC_H238*p*AcF_ complex were obtained from 0.2 M (NH_4_)_2_SO_4_, 0.1 M Bis-TRIS, pH 5.8, 19% PEG3350. Crystals of **4**-bound PikC_H238*p*AcF_ complex were obtained from 0.2 M Li_2_SO_4_, 0.1 M Bis-TRIS, pH 6.0, 18% PEG4000. The **5**-bound PikC_H238*p*AcF_ crystals were grown under the same crystalization conditions as **4**-bound PikC_H238*p*AcF_. Before data collection, the crystals were cryo-protected by plunging them into a drop of reservoir solution supplemented with 25% glycerol. The data were collected at beamline BL19U (SSRF, China) under cryogenic conditions at 100 K. Data indexing, integration, and scaling were conducted using HKL3000 software suite^64^. Crystal structures were determined by molecular replacement using the atomic coordinates of the PDB ID code 2BVJ as a search model. The models were built into the electron density using COOT^65^ and refined in PHENIX^66^. The molecules of **4**–**6** were modeled at the final stage of refinement, and the model quality was verified using the program MolProbity in PHENIX. Structural models were drawn using the program PyMOL version 2.3.2 (https://pymol.org/2/).

### AutoDock analysis

The initial structures of **7** were generated by ChemDraw 17.0, followed by DFT optimization at the B3LYP-D3/def2SVP^67^ theoretical level with the Gaussian16 (Revision A.03) package^68^. Automated molecular docking was performed using the DFT optimized structure by running the AutoDock vina (version 1.5.7) program equipped with ADT. All side chains were set as rigid body and grid spacing was set to 0.5 Å. Other parameters remained as their default values. The top 9 lowest energy docking poses of **7** in PikC_H238*p*AcF_ from 2,500,000 searching results were output, among which the ideal catalytic conformation was used for analysis as shown in Fig. 5. The docking models were analyzed and represented using ADT and PyMOL version 2.3.2 (https://pymol.org/2/).

### Statistics and reproducibility

All experiments in this study were carried out in duplicate or triplicate, and statistical analyses were performed using the Student’s *t* test, two-sided. Basic statistical analysis was performed using Microsoft Excel 2016 (version 16.0.4266.1001).

### Figures preparation

Figures and schemes were prepared using Adobe Illustrator CC 2021 (version 25.2.1), ChemDraw (version 17.0), and PowerPoint 2016 (version 16.0.4266.1001).

### Reporting summary

Further information on research design is available in the Nature Portfolio Reporting Summary linked to this article.

## Supplementary information

Supplementary Information

Reporting Summary

## Data Availability

Atomic coordinates and structure factors for substrate-free PikC_H238*p*AcF_, **4**-bound PikC_H238*p*AcF_, **5**-bound PikC_H238*p*AcF_ and **6**-bound PikC_H238*p*AcF_ have been deposited in the Protein Data Bank under the accession numbers 7XBM, 7XBN, 8GUE and 7XBO, respectively. Previously published structures used in this study include PDB IDs: 2BVJ (the crystal structure of ligand-free PikC_WT_), 2C6H (the crystal structure of **4**-bound PikC_WT_), 2C7X (the crystal structure of **5**-bound PikC_WT_), 2VZ7 (the crystal structure of **4**-bound PikC_D50N_), and 2VZM (the crystal structure of **5**-bound PikC_D50N_). All other relevant data supporting the findings of this study are available in Supplementary Information. Source data are provided with this paper.

## Source data

Source Data
