# Supplementary material for: Unnatural activities and mechanistic insights of cytochrome P450 PikC gained from site-specific mutagenesis by non-canonical amino acids
Source: Nat Commun. 2023 Mar 25;14:1669. doi: 10.1038/s41467-023-37288-0 (PMC10039885; doi:10.1038/s41467-023-37288-0)
Supplement: Supplementary file 1 — Supplementary Information [file 41467_2023_37288_MOESM1_ESM.pdf]

Supplementary Information

Unnatural activities and mechanistic insights of cytochrome P450 PikC gained from site-specific mutagenesis by non-canonical amino acids

Yunjun Pan[1,#], Guobang Li[1,#], Ruxin Liu[1], Jiawei Guo[1], Yunjie Liu[1], Mingyu Liu[1], Xingwang Zhang[1,2], Luping Chi[1], Kangwei Xu[3], Ruibo Wu[3], Yuezhong Li[1], Yuzhong Zhang[1,2], Xiang Gao[1*] and Shengying Li[1,2*]

[1]State Key Laboratory of Microbial Technology, Shandong University, Qingdao, Shan-dong 266237, China

[2]Laboratory for Marine Biology and Biotechnology, Qingdao National Laboratory for Marine Science and Technology, Qingdao, Shandong 266237, China

[3]School of Pharmaceutical Sciences, Sun Yat-sen University, Guangzhou 510006, P.R. China

[#]These authors contributed equally to this work.

[*]To whom correspondence should be addressed: lishengying@sdu.edu.cn (S.L.), xgao@email.sdu.edu.cn (X.G.)

Table of Contents

Supplementary Tables 3

Supplementary Table 1. The values of dissociation constants (K_D) for natural PikC substrates and their corresponding aglycones. 3

Supplementary Table 2. The oligonucleotides used for *desVII* and *pikC* gene knockout. 3

Supplementary Table 3. The oligonucleotides used in site-directed mutagenesis. 4

Supplementary Table 4. The yields of PikC proteins. 4

Supplementary Table 5. NMR data for 10-DML (**6**) and NBL (**7**). 5

Supplementary Table 6. NMR data for methynolide (**8**) and neomethynolide (**9**). 6

Supplementary Table 7. NMR data for compound **11**, **12** and **14**. 7

Supplementary Table 8. NMR data for compound **13** and **15**. 9

Supplementary Table 9. Data collection and refinement statistics. 10

Supplementary Figures 12

Supplementary Tables

Supplementary Table 1. The values of dissociation constants (K_D) for natural PikC substrates and their corresponding aglycones.

	K_D (μM)			
	YC-17 (**4**)	Narbomycin (**5**)	10-Deoxymethynolide (**6**)	Narbononolide (**7**)
$PikC_{WT}$	177.6 ±16.1	373.6±59.7	ND	ND
$PikC_{H238\textit{p}AcF}$	40.2 ±0.6	87.1 ±28.9	207.5 ±43.2	236.9 ±59.2
$PikC_{H238\textit{p}AcF/E85Q}$	118.6 ±10.4	15.2 ±0.6	162.5 ±26.3	166.1 ±34.1
$PikC_{H238F}$	11.2 ±0.6	72.7 ±4.4	ND	ND
$PikC_{H238Y}$	16.7 ±0.9	63.4 ±7.6	ND	ND

ND: Not determined since the binding curves could not be fitted.

Supplementary Table 2. The oligonucleotides used for *desVII* and *pikC* gene knockout.

Gene	Primer	Sequence (5'-3')
sgRNA-DesVII	DesVIIsgRNA-F	tataatactagtGTGCGTGTGATGTGCGAACGgttttagagctagaaatagcaag
	DesVIIsgRNA-R	ctctaaaacCGTTCGCACATCACACGCACactagtattatacctaggactgagctagc
DesVII-up	DesVII-up-F	ggtgctttttttgagatctgaattccGGCGTCGTGTCCTTCCTTGCC
	DesVII-up-R	CTTCTACCTCAACGACCAGTGGC
DesVII-down	DesVII-dn-F	GCCACTGGTCGTTGAGGTAGAAGcgttcacggagaagcggttgttc
	DesVII-dn-R	cgacggccagtgccaagcttTCACCCCTGTATCTGCGCCG
sgRNA-PikC	PikCsgRNA-F	tcagtcctaggtataatactagtCGCCAGTCCTTGCTGAACCGgttttagagctagaaatag
	PikCgRNA-R	ctatttctagctctaaaacCGGTTCAGCAAGGACTGGCGactagtattatacctaggactga
PikC-up	PikC-up-F	caccgagtcggtgctttttttgagatctgaattccCGGGTGGCTGGGCGGCAGTT
	PikC-up-R	TGAACCCGCACGTCACCCATggagaactccagaccgggcc
PikC-down	PikC-dn-F	gtgacaaggagtcgtaatgggtgacgtgcgggttcaGGAGAACTCCAGACCGGGCC
	PikC-dn-R	gtaaaacgacggccagtgccaagcttACGCAGCAGGTCGGCGACCC

Note: Lowercase letters denote homology arms

Supplementary Table 3. The oligonucleotides used in site-directed mutagenesis.

Gene	Primer	Sequence (5’-3’)
PikC$_{\text{WT}}$	PikC-F	ctggtgccgcgcggcagccatatgCGCCGTACCCAGCAGGGAAC
	PikC-R	ctcagtggtggtggtggtggtgctcgagACCGGTACGGCGGCCCGCC
PikC$_{\text{H238TAG}}$	H238TAG-F	ccgaggagctgctcggtatggccTAGatcctgctcgtcgcggggcacgag
	H238TAG-R	ctcgtgccccgcgacgagcaggatCTAggccataccgagcagctcctcgg
PikC$_{\text{E85TAG}}$	E85TAG-F	ccacgactcccctgaccgaggccTAGgccgcgctcaaccacaacatgctg
	E85TAG-R	cagcatgttgtggttgagcgcggcCTAggcctcggtcaggggagtcgtgg
PikC$_{\text{E94TAG}}$	E94TAG-F	ccgcgctcaaccacaacatgctgTAGtccgacccgccgcggcacacccgg
	E94TAG-R	ccgggtgtgccgcggcgggtcggaCTAcagcatgttgtggttgagcgcgg
PikC$_{\text{F178TAG}}$	F178TAG-F	gccttccgcgtctggaccgacgccTAGgtcttcccggacgatcccgccca
	F178TAG-R	tgggcgggatcgtccgggaagacCTAggcgtcggtccagacgcggaaggc
PikC$_{\text{E85Q}}$<br>PikC$_{\text{H238TAG/E85Q}}$<br>PikC$_{\text{H238TAG/E85Q/E94Q}}$<br>PikC$_{\text{H238TAG/E85Q/E94A}}$	E85Q-F	ccacgactcccctgaccgaggccCAGgccgcgctcaaccacaacatgctg
	E85Q-R	cagcatgttgtggttgagcgcggcCTGggcctcggtcaggggagtcgtgg
PikC$_{\text{E94Q}}$<br>PikC$_{\text{H238TAG/E94Q}}$<br>PikC$_{\text{H238TAG/E85Q/E94Q}}$<br>PikC$_{\text{H238TAG/E85A/E94Q}}$	E94Q-F	ccgcgctcaaccacaacatgctgCAGtccgacccgccgcggcacacccgg
	E94Q-R	ccgggtgtgccgcggcgggtcggaCTGcagcatgttgtggttgagcgcgg
PikC$_{\text{E85A}}$<br>PikC$_{\text{H238TAG/E85A}}$<br>PikC$_{\text{H238TAG/E85A/E94A}}$<br>PikC$_{\text{H238TAG/E85A/E94Q}}$	E85A-F	ccacgactcccctgaccgaggccGCGgccgcgctcaaccacaacatgctg
	E85A-R	cagcatgttgtggttgagcgcggcCGCggcctcggtcaggggagtcgtgg
PikC$_{\text{E94A}}$<br>PikC$_{\text{H238TAG/E94A}}$<br>PikC$_{\text{H238TAG/E85A/E94A}}$<br>PikC$_{\text{H238TAG/E85Q/E94A}}$	E94A-F	ccgcgctcaaccacaacatgctgGCGtccgacccgccgcggcacacccgg
	E94A-R	ccgggtgtgccgcggcgggtcggaCGCcagcatgttgtggttgagcgcgg

Supplementary Table 4. The yields of PikC proteins.

Enzyme	Protein yield (mg/L)	Enzyme	Protein yield (mg/L)
PikC$_{\text{WT}}$	9.40	PikC$_{\text{H238OMeY}}$	3.55
PikC$_{\text{H238BipA}}$	2.12	PikC$_{\text{H238}p\text{BrF}}$	6.90
PikC$_{\text{H238}p\text{AzF}}$	4.26	PikC$_{\text{H238O}t\text{BuY}}$	7.25
PikC$_{\text{H238}p\text{ENF}}$	7.28	PikC$_{\text{H238}p\text{PrF}}$	2.33
PikC$_{\text{H238}p\text{AcF}}$	6.67		

Supplementary Table 5. NMR data for 10-DML (**6**) and NBL (**7**).

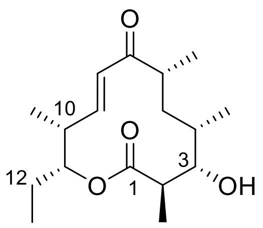

10-Deoxymethynolide (**6**)

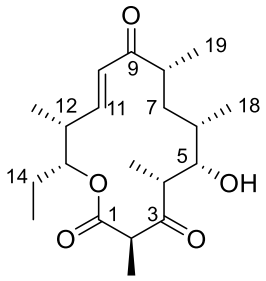

Narbonolide (**7**)

Position	10-Deoxymethynolide (**6**)		Narbonolide (**7**)	
	^{13}C (ppm)	^{1}H [mult., J (Hz)]	^{13}C (ppm)	^{1}H [mult., J (Hz)]
1	175.1		170.9	
2	43.0	2.47 (1H, dd, 8.6, 5.2)	50.3	3.71 (1H, m)
3	76.4	3.18 (1H, ddd, 10.3, 6.5, 1.1)	207.7	
4	33.0	0.95 (1H, m)	50.2	2.69 (1H, dd, 9.5, 4.4)
5	33.1	1.15 (1H, m), 1.57 (1H, m)	72.6	3.87 (1H, m)
6	45.1	2.27 (1H, m)	35.1	1.69 (1H, m)
7	204.1		36.3	1.48 (1H, m), 1.68 (1H, m)
8	125.5	6.43 (1H, dd, 15.8, 5.4)	39.8	3.01 (1H, m)
9	147.1	6.54 (1H, dd, 15.8, 1.1)	204.7	
10	37.6	2.61 (1H, m)	148.5	6.89 (1H, m
11	73.4	4.82 (1H, ddd, 8.9, 5.1, 2.2)	129.0	6.1 (1H, m)
12	24.8	1.53 (2H, m)	38.8	2.70 (1H, d, 6.9)
13	10.4	0.81 (3H, d, 7.4)	78.1	5.12 (1H, d, 28.1)
14	16.6	1.14 (3H, m)	24.2	1.67 (2H, m)
15	17.1	1.13 (3H, m)	10.4	0.90 (3H, t, 5.6)
16	17.6	0.83 (3H, d, 5.2)	14.3	1.35 (3H, d, 7.0)
17	9.6	1.04 (3H, d, 6.9)	18.1	1.12 (3H, d, 5.2)
18			18.6	0.93 (3H, d, 4.1)
19			14.3	1.25 (3H, m)
20			10.9	1.15 (3H, d, 6.6)

Supplementary Table 6. NMR data for methynolide (**8**) and neomethynolide (**9**).

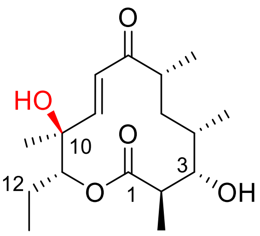

Methynolide (**8**)

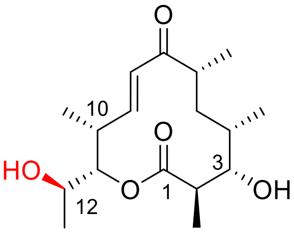

Neomethynolide (**9**)

Position	Methynolide (**8**)		Neomethynolide (**9**)	
	DEPTQ (ppm)	^{1}H [mult., J (Hz)]	^{13}C (ppm)	^{1}H [mult., J (Hz)]
1	175.2		174.0	
2	43.1	2.52 (1H, m)	42.9	2.51 (1H, m)
3	76.0	3.23 (1H, m)	76.1	3.22 (1H, m)
4	32.8	0.98 (1H, m)	33.0	1.17 (1H, m)
5	33.3	1.15 (1H, m) 1.65 (1H, m)	33.6	1.16 (1H, m), 1.51 (1H, m)
6	45.3	2.32 (1H, m)	45.2	2.31 (1H, m)
7	204.1		204.0	
8	124.6	6.40 (1H, d, 16.0)	125.8	6.48 (1H, m)
9	150.2	6.39 (1H, d, 16.0)	147.5	6.55 (1H, m)
10	73.2		35.2	2.95 (1H, m)
11	76.2	4.54 (1H, dd, 10.8, 2.0)	75.5	4.60 (1H, m)
12	20.6	1.86 (1H, m)<br>1.39 (1H, m)	64.5	3.63 (1H, m)
13	10.9	0.78 (3H, t, 7.4)	20.9	0.98 (3H, d, 6.1)
14	16.8	1.16 (3H, t, 13.4)	17.1	1.14 (3H, m)
15	17.1	0.83 (3H, d, 6.6)	17.7	0.84 (3H, d, 6.5)
16	17.7	1.12 (3H, d, 6.9)	16.4	1.14 (3H, m)
17	18.9	1.22 (3H, s)	9.60	1.06(3H, d, 6.8)

Supplementary Table 7. NMR data for compound **11**, **12** and **14**.

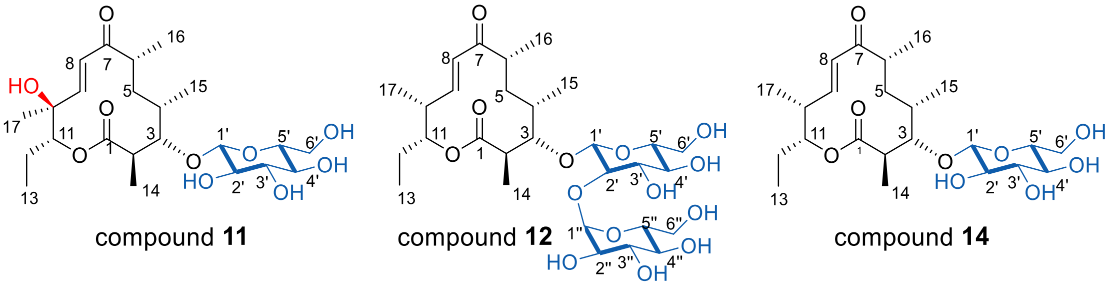

Position	11		12		14	
	DEPTQ	^{1}H [mult., J (Hz)]	^{13}C	^{1}H [mult., J (Hz)]	DEPTQ	^{1}H [mult., J (Hz)]
1	175.1		175.3		174.9	
2	43.5	2.80 (1H, m)	43.1	2.97 (1H, m)	43.5	2.74 (1H, dd, 10.5, 7.0)
3	85.2	3.42 (1H, m)	84.8	3.41 (1H, m)	85.1	3.41 (1H, m)
4	33.1	1.02 (1H, m)	33.2	1.02 (1H, m)	33.0	1.04 (1H, m)
5	33.4	1.18 (1H, m)<br>1.71 (1H, m)	32.6	1.76 (1H, m)<br>1.19 (1H, m)	33.7	1.66 (1H, m)<br>1.71 (1H, m)
6	45.3	2.35 (1H, m)	45.1	2.31 (1H, m)	44.9	2.33 (1H, m)
7	203.5		203.7		203.7	
8	124.6	6.48 (1H, d, 16.0)	124.8	6.37 (1H, d, 16.1)	124.6	6.61 (1H, dd, 15.8, 5.4)
9	150.3	6.55 (1H, d, 16.0)	150.4	6.40 (1H, d, 16.1)	150.1	6.54 (1H, dd, 15.8, 1.0)
10	73.1		73.2		37.6	2.66 (1H, s)
11	76.1	4.52 (1H, dd, 10.8, 2.0)	75.7	4.5 (1H, dd, 10.8, 1.9)	73.5	4.84 (1H, ddd, 8.8, 5.2, 2.1)
12	20.7	1.39 (1H, m)<br>1.86 (1H, m)	20.6	1.83 (1H, t, 13.4)<br>1.36 (1H, ddd, 14.1, 10.8, 7.2)	24.8	1.55 (1H, m)<br>1.58 (1H, m)
13	10.9	0.78 (3H, t, 10.4	10.9	0.79 (3H, t, 7.4)	10.4	0.81 (3H, t, 7.4)
14	16.1	1.33 (3H, d, 6.9)	16.7	1.33 (3H, d, 6.9)	16.0	1.30 (3H, d, 6.9)
15	17.5	0.92 (3H, d, 6.7)	17.4	0.90 (3H, d, 6.7)	17.5	0.91 (3H, d, 6.7)
16	16.1	1.12 (3H, d, 7.0)	17.0	1.12 (3H, d, 7.0)	17.1	1.12 (3H, d, 7.0)
17	18.9	1.22 (3H, s)	18.9	1.22 (3H, s)	9.6	1.04 (3H, d, 2.2)
1'	104.1	4.14 (1H, d, 7.8)	102.7	4.29 (1H, m)	104.1	4.13 (1H, d, 7.8)

2'	74.2	2.93 (1H, dd, 8.8, 8.0)	82.6	3.20 (1H, m)	74.2	2.93 (1H, t, 8.4)
3'	77.2	3.12 (1H, t, 8.8)	77.8	3.11 (1H, m)	77.2	3.12 (1H, t, 8.8)
4'	70.4	3.03 (1H, d, 8.7)	70.4	3.07 (1H, m)	70.4	3.03 (1H, d, 8.8)
5'	76.7	3.06 (1H, dd, 5.7, 2.1)	76.6	3.12 (1H, m)	76.7	3.06 (1H, dd, 5.7, 2.1)
6'	61.4	3.64 (1H, dd, 11.4, 2.0)<br>3.42 (1H, dd, 7.7, 4.3)	61.1	3.65 (1H, dd, 10.3, 4.6)<br>3.76 (1H, m)	61.4	3.64 (1H, dd, 11.4, 1.9)<br>3.4 (1H, m)
1"			104.9	4.37 (1H, d, 7.8)		
2"			75.1	3.00 (1H, m)		
3"			76.8	3.11 (1H, m)		
4"			72.7	3.42 (1H, m)		
5"			76.0	3.37 (1H, m)		
6"			61.6	3.49 (1H, dt, 11.4, 5.7), 3.40 (1H, m)		[illegible]

Supplementary Table 8. NMR data for compound **13** and **15**.

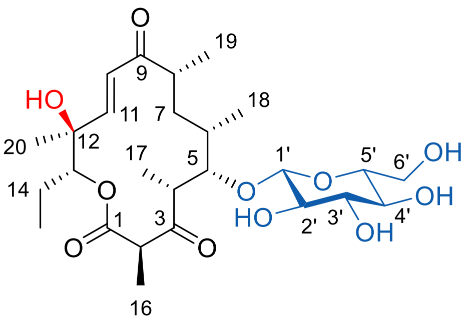

compound **13**

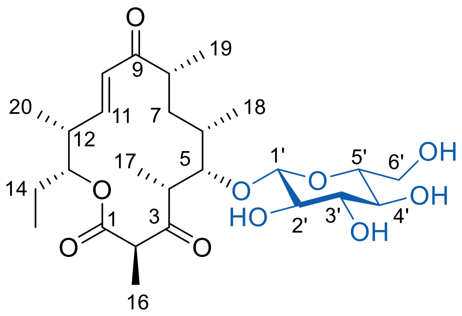

compound **15**

Position	**13**		**15**	
	DEPTQ (ppm)	^{1}H [mult., J (Hz)]	^{13}C (ppm)	^{1}H [mult., J (Hz)]
1	169.9		170.4	
2	50.2	4.01 (1H, q, 6.9)	50.6	4.00 (1H, q, 6.9)
3	208.2		207.9	
4	50.1	2.90 (1H, m)	50.5	2.86 (1H, p, 7.4)
5	79.7	3.97 (1H, m)	79.9	3.94 (1H, m)
6	36.9	1.36 (1H, m)	33.5	1.82 (1H, m)
7	34.8	1.15 (1H, m)<br>1.63 (1H, m)	35.4	1.15 (1H, m)<br>1.55 (1H, m)
8	43.0	2.57 (1H, dd, 3.7, 1.8)	43.3	2.53 (1H, dd, 3.7, 1.9)
9	203.0		202.5	
10	150.6	6.6 (1H, d, 15.9)	148.8	6.72 (1H, m)
11	123.0	6.25 (1H, d, 16.6)	126.9	6.22 (1H, dd, 14.9, 8.9)
12	74.4		38.8	2.67 (1H, m)
13	79.7	4.66 (1H, dd, 10.6, 2.1)	77.4	4.87 (1H, m)
14	20.8	1.36 (1H, m)<br>1.89 (1H, m)	23.4	1.57 (2H, m)
15	11.1	0.81 (3H, t, 7,4)	10.6	0.85 (3H, t, 7.4)
16	16.3	1.18 (3H, t, 5.6)	15.4	1.21 (3H, d, 6.9)
17	13.8	1.25 (3H, m)	14.0	1.25 (3H, 7.4)
18	17.0	0.93 (3H, t, 7.2)	17.6	0.93 (3H, t, 6.4)
19	16.8	1.04 (3H, t, 6.3)	16.7	1.06 (3H, d, 6.9)
20	20.1	1.24 (3H, s)	11.4	1.01 (3H, d, 6.7)
1’	103.7	4.14 (1H, d, 7.8)	103.6	4.11 (1H, d, 7.8)
2'	74.1	2.94 (1H, 8.8)	75.2	2.93 (1H, dt, 8.3, 4.3)
3'	77.4	3.12 (1H, m)	77.8	3.11 (1H, m)
4'	70.5	3.06 (1H, m)	70.3	3.06 (1H, m)
5'	76.7	3.09 (1H, m)	77.1	3.05 (1H, m)
6'	61.6	3.45 (1H, m)<br>3.67 (1H, d, 11.1)	61.4	3.44 (1H, t, 13.4)<br>3.66 (1H, d, 11.0)

Supplementary Table 9. Data collection and refinement statistics.

	Substrate free	YC-17-bound	10-DML bound	Narbomycin-bound
PDB ID	7XBM	7XBN	7XBO	8GUE
**Data collection**				
Wavelength	0.9785	0.9785	0.9785	0.9792
Resolution range	50.00 – 2.40 (2.50 – 2.40)	50.00 – 2.00 (2.07 – 2.00)	50.00 – 2.20 (2.28 – 2.20)	50.00 – 1.90 (1.97 – 1.90)
Space group	P21 21 21	P21 21 21	P21 21 21	P21 21 21
a b c	60.040 108.331 153.300	59.823 108.903 152.630	60.119 108.480 153.147	59.465 97.818 141.075
α β γ	90	90	90	90
Unique reflections	39557 (3737)	67865 (6600)	50823 (4739)	65500 (6460)
Completeness (%)	99.5 (95.9)	99.7 (98.5)	98.9 (93.9)	100 (99.9)
Mean I/sigma(I)	24.5 (2.3)	26.8 (3.3)	14.5 (2.0)	15.8(1.5)
Wilson B-factor	34.7	25.2	29.6	33.2
R-meas	0.113(0.687)	0.100 (0.761)	0.173 (0.713)	0.116(1.164)
CC1/2	1.000 (0.882)	0.998 (0.877)	0.995 (0.783)	0.994 (0.710)
Data redundancy	12.4 (9.6)	12.6 (10.5)	11.8 (8.5)	12.6 (10.8)
Refinement				
Resolution range	23.51 - 2.40 (2.49 - 2.40)	27.23 - 2.00 (2.07 - 2.00)	32.7 - 2.20 (2.28 - 2.20)	36.89- 1.90 (1.97 - 1.90)
Reflections used in refinement	36758 (2502)	64178 (4975)	44808 (3069)	65377 (6267)
Reflections used for R-free	2000 (136)	1998 (155)	1999 (137)	1999 (191)
R-work	0.1837 (0.2259)	0.1745 (0.1960)	0.1790 (0.1934)	0.2009 (0.2957)
R-free	0.2299 (0.3178)	0.2095 (0.2330)	0.2122 (0.2610)	0.2506 (0.3189)
Number of non-hydrogen atoms	6697	7208	6811	6672
macromolecules	6150	6136	6141	6128
ligands	162	195	190	243
solvent	385	877	480	301
Protein residues	788	786	787	786
RMS (bonds)	0.005	0.004	0.005	0.006
RMS (angles)	0.65	0.62	0.71	0.82
Ramachandran favored (%)	97.94	98.2	97.94	97.29
Ramachandran allowed (%)	1.93	1.8	2.06	2.71
Ramachandran outliers (%)	0.13	0	0	0
Rotamer outliers (%)	1.09	0.47	0.78	0.16

Clashscore	5.42	2.22	4.86	5.22
Average B-factor	40.8	29.12	36.15	37.65
macromolecules	40.86	28.06	35.94	37.16
ligands	42.06	27.51	34.17	41.83
solvent	39.25	36.91	39.61	38.96

Supplementary Figures

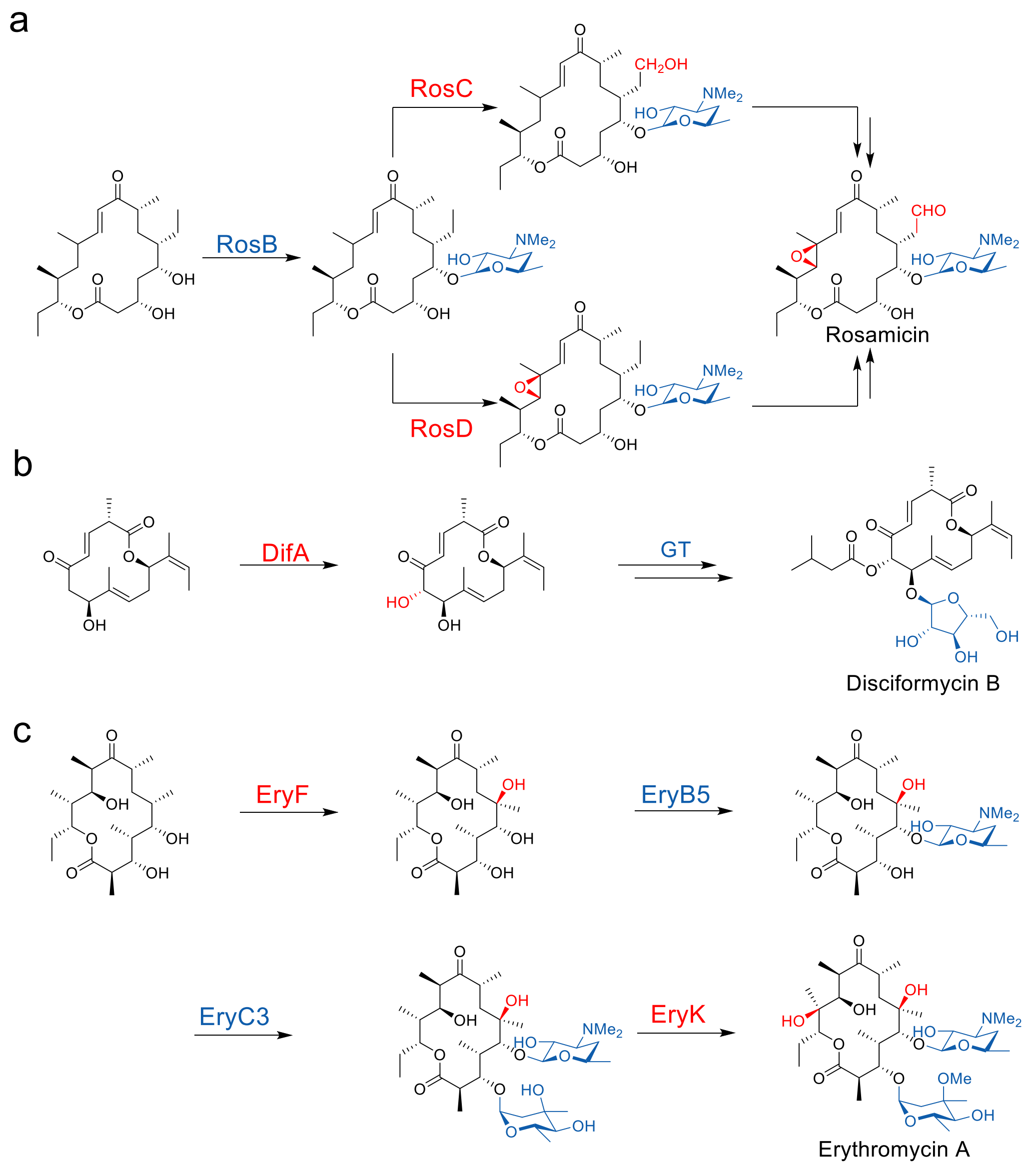

Supplementary Figure 1. Additional representative macrolide biosynthetic pathways. **a**, The rosamicin biosynthetic pathway in which the glycosyltransferase-mediated sugar attachment precedes the P450-catalyzed oxidation. **b**, The partial disciformycin biosynthetic pathway with an order of oxidation-glycosylation. **c**, The erythromycin biosynthesis with staggered monooxygenation and glycosylation steps. The functional groups introduced by P450 enzymes and glycosyltransferases are highlighted in red and blue, respectively.

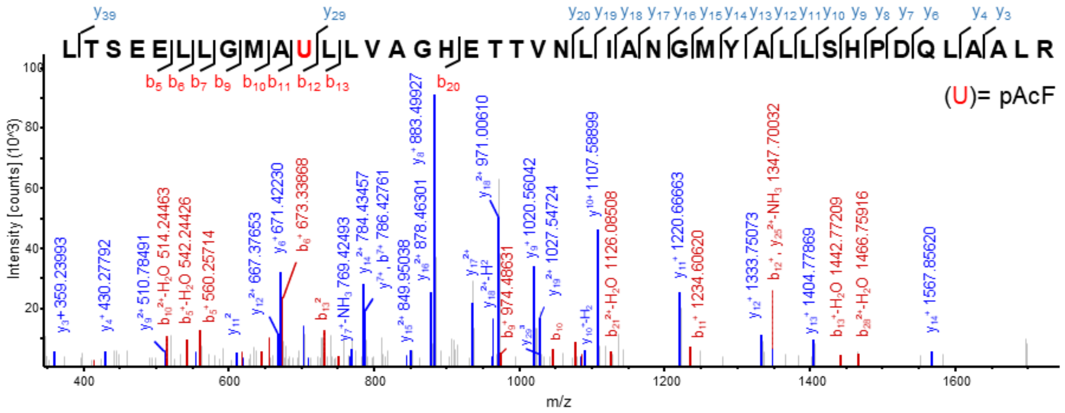

Supplementary Figure 2. Tandem MS analysis of the purified PikC$_{H238pAcF}$.

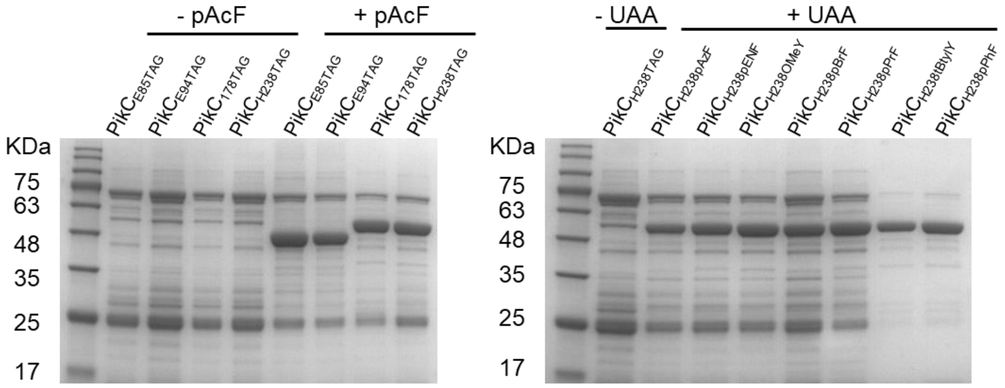

Supplementary Figure 3. SDS-PAGE analysis of proteins with incorporated ncAAs. The uncropped scans of gels are provided in the Source Data file.

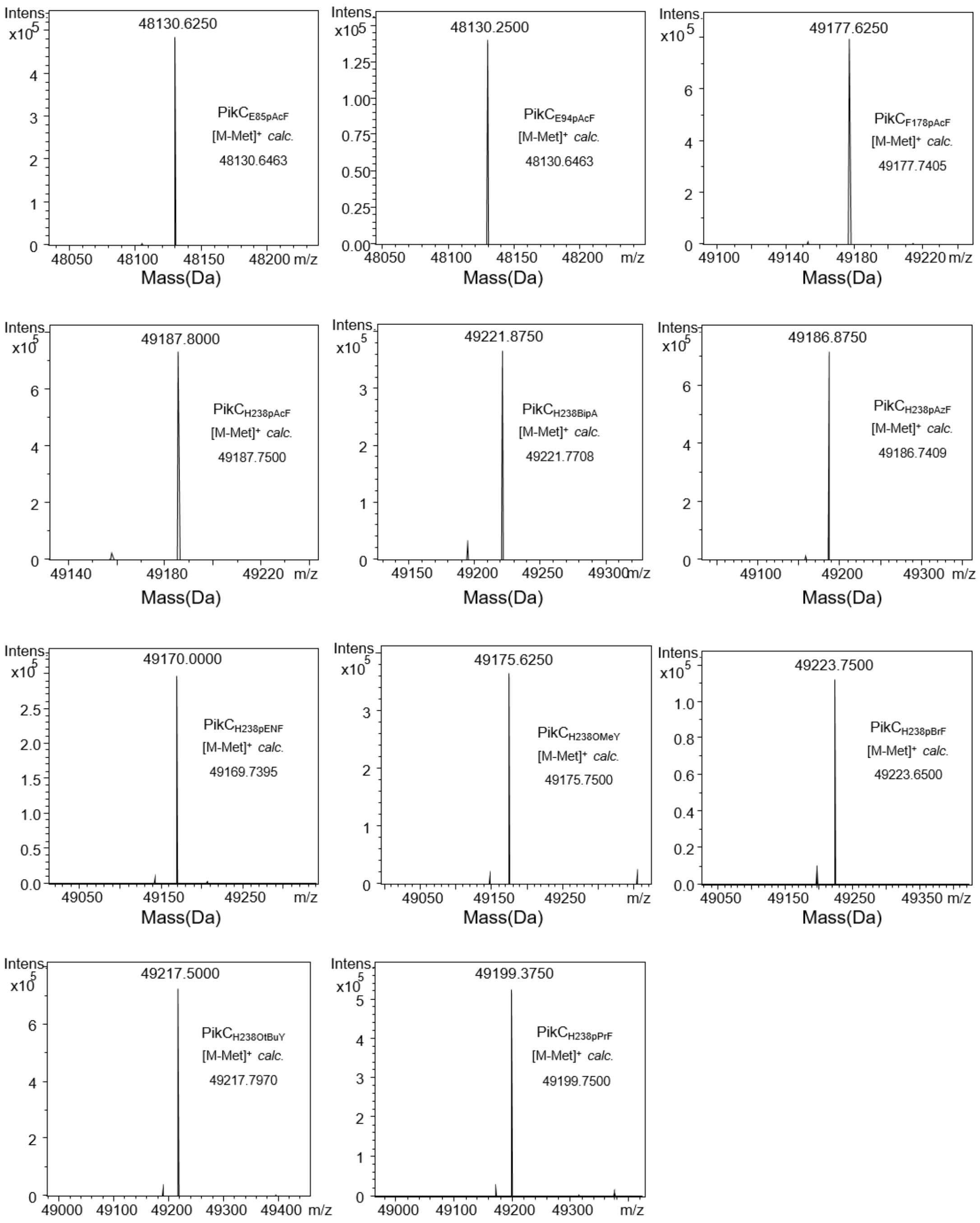

Supplementary Figure 4. Deconvoluted mass spectra of PikC ncAA mutants.

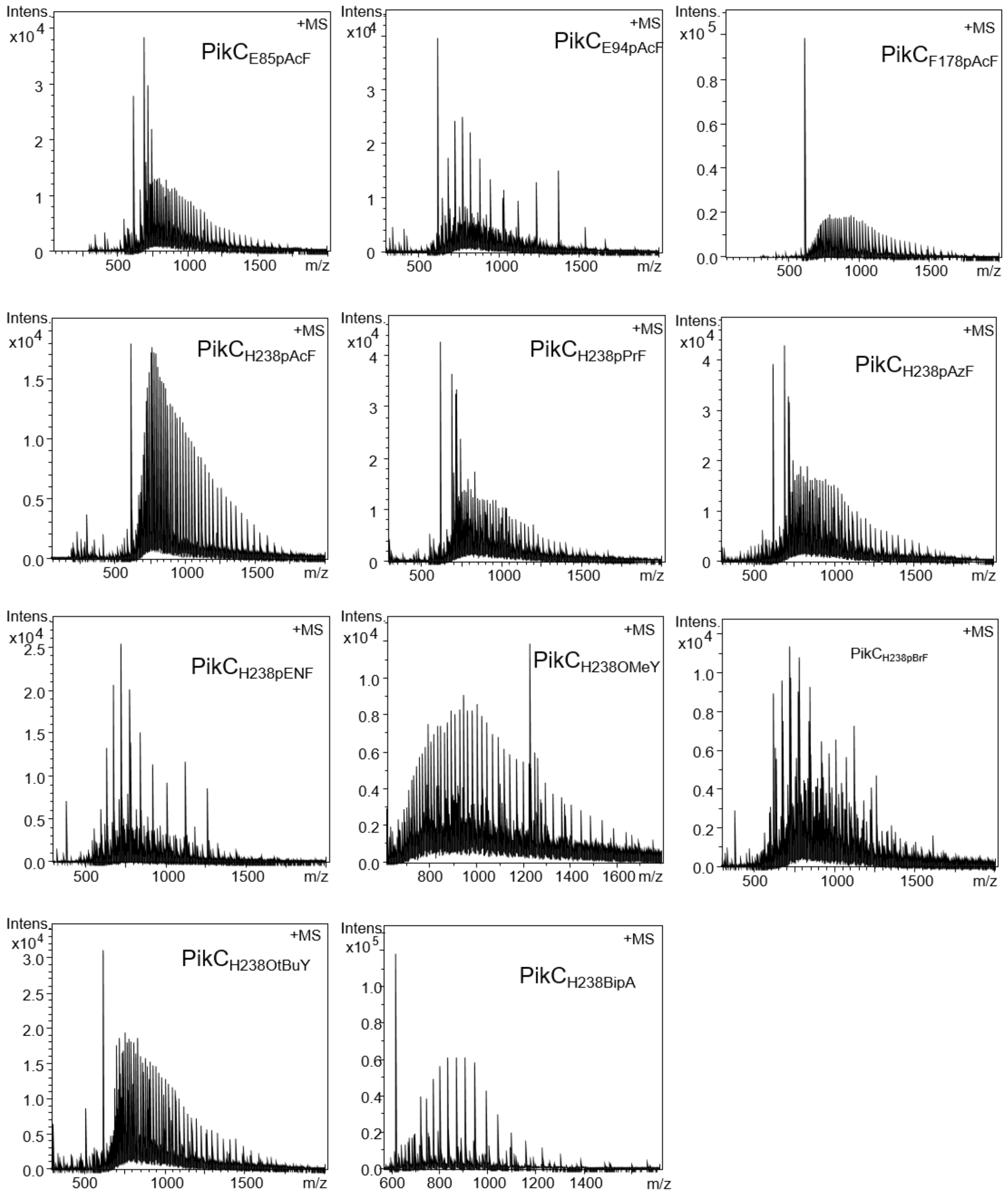

Supplementary Figure 5. Original mass spectra of PikC ncAA mutants.

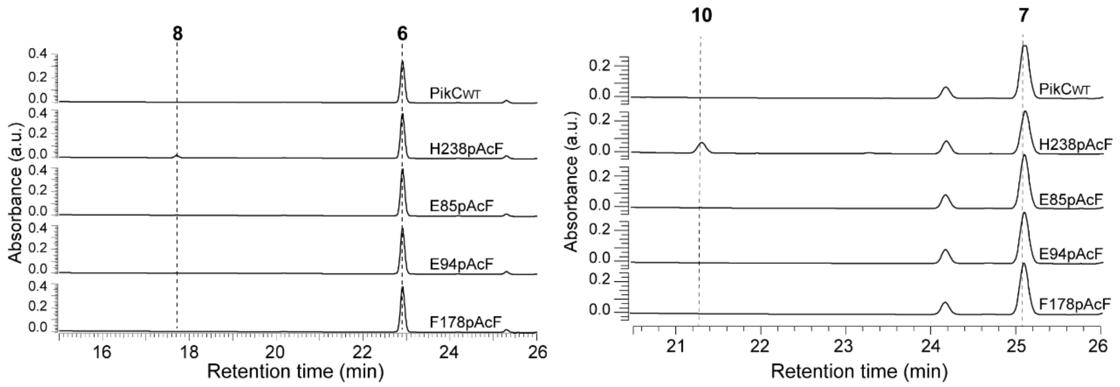

Supplementary Figure 6. HPLC analysis (230 nm) of the *in vitro* assays of PikC ncAA mutants. Different residues in the desosamine binding pocket were individually replaced by *p*AcF.

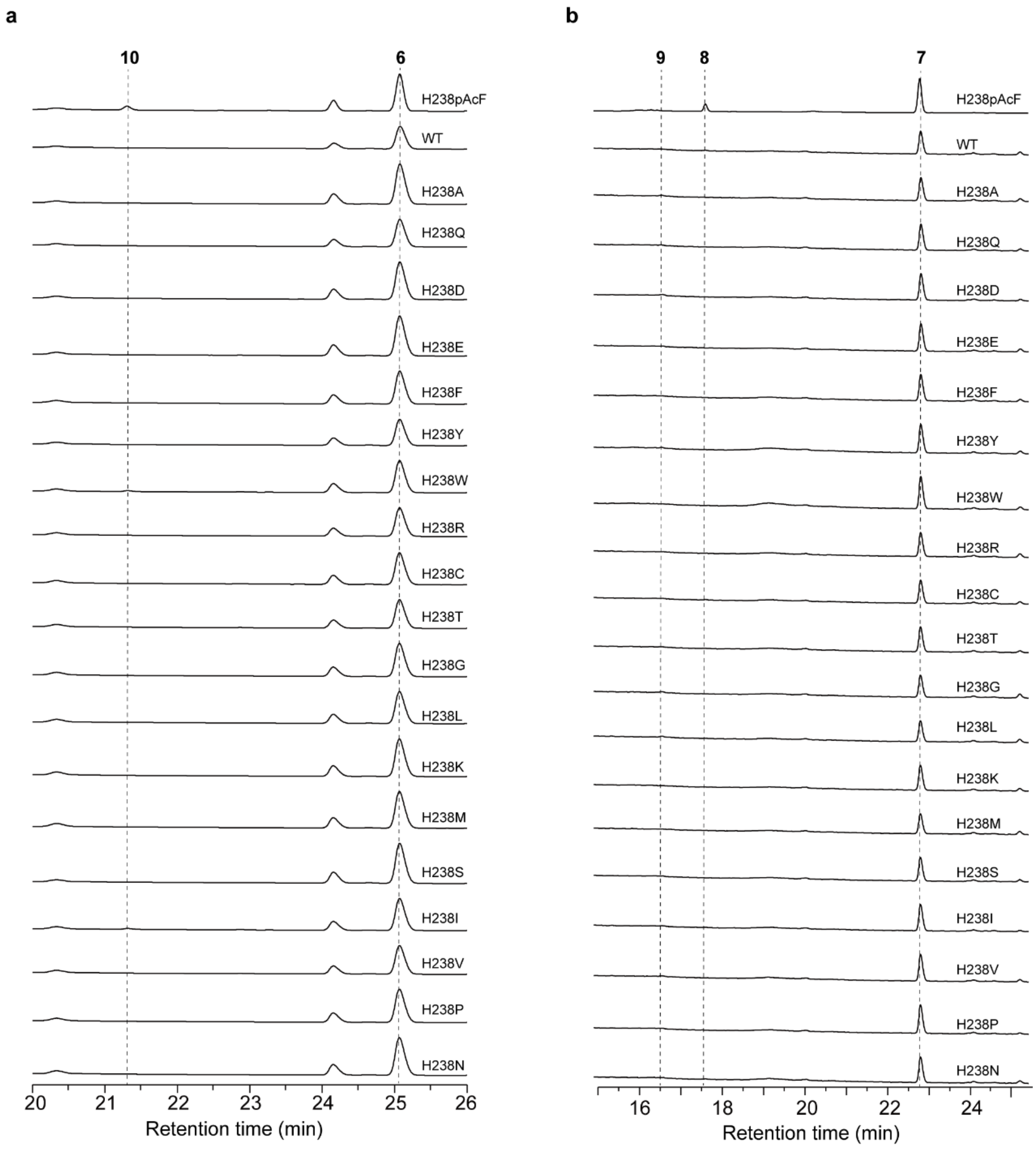

Supplementary Figure 7. HPLC analysis (230 nm) of the *in vitro* activities of PikC variants toward **6** (**a**) and **7** (**b**), of which His238 was individually replaced by each of twenty proteinogenic amino acids. Each individual *in vitro* enzymatic reaction containing 1 μM PikC (wild type or mutant), 1 mM NADPH, 10 μM Fdx1499, 5 μM FdR0978, and 0.5 mM substrate in 100 μL storage buffer was incubated at 30 °C for 40 min.

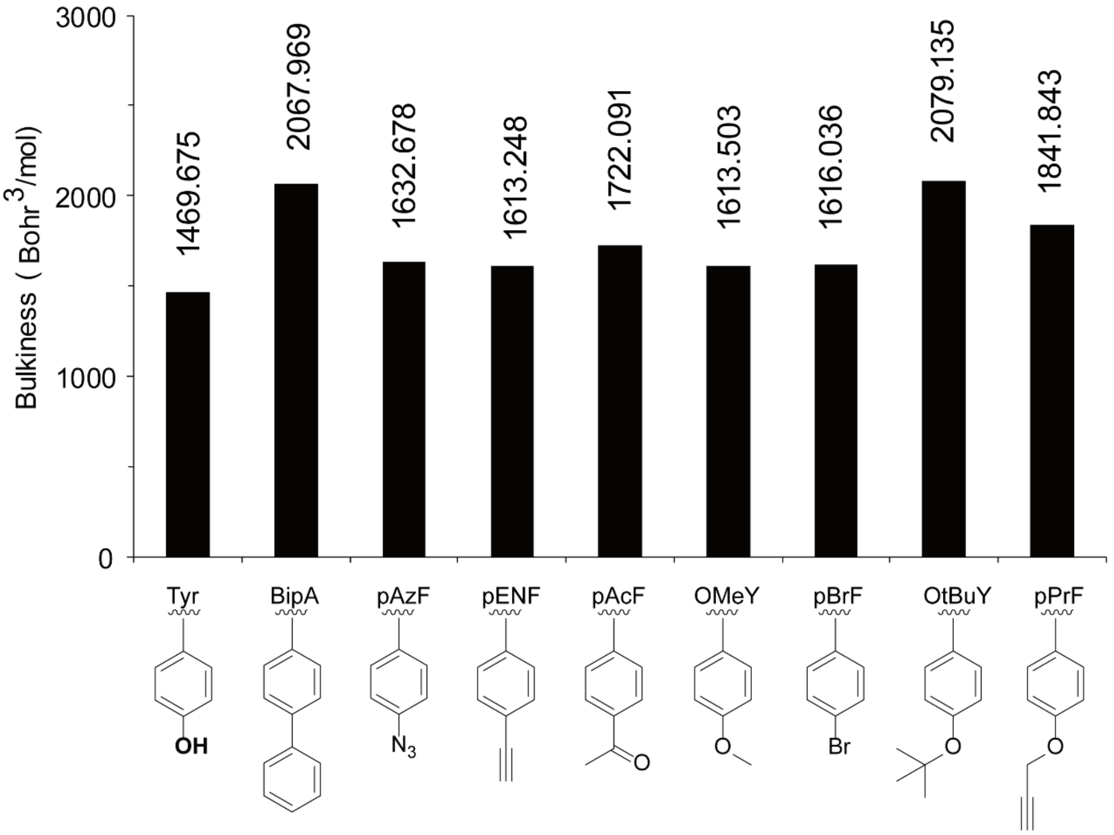

Supplementary Figure 8. Calculation of van der Waals volumes of the ncAAs used in this study. The molecules are first optimized at the B3LYP/6-31G* theoretical level. The volumes are calculated on the basis of the van der Waals surfaces defined as an electron density equal to 0.001 au.

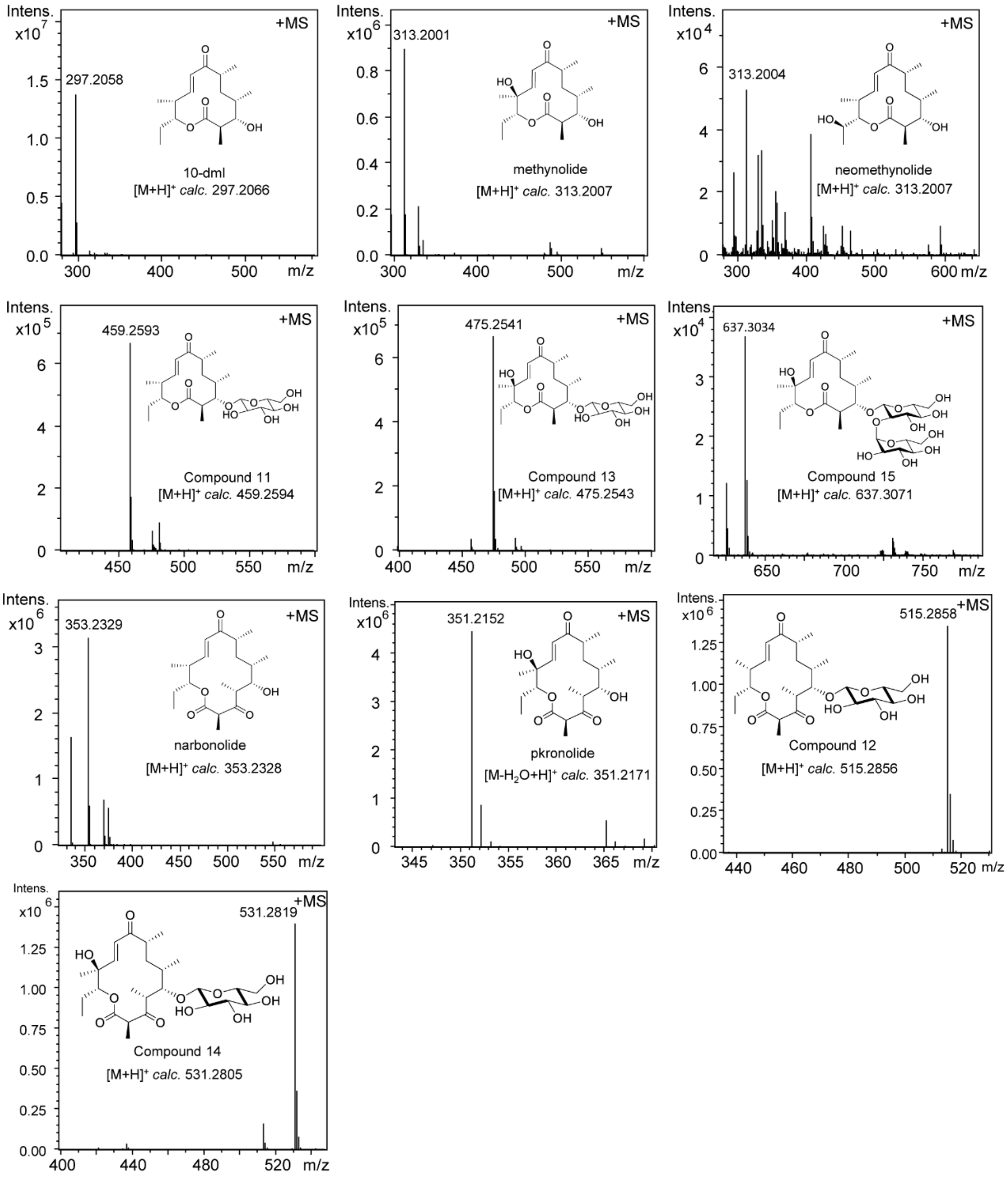

Supplementary Figure 9. HRMS spectra of main compounds in this study.

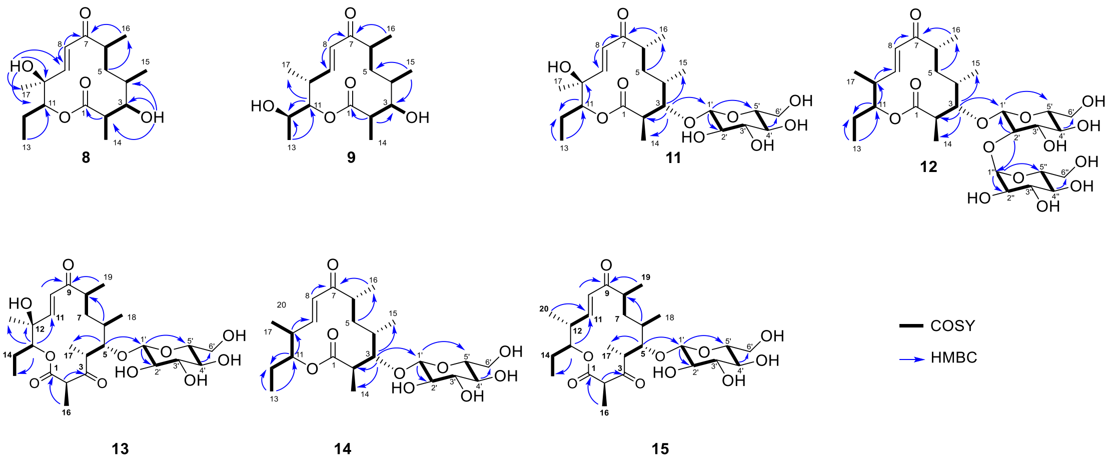

Supplementary Figure 10. COSY and HMBC correlations.

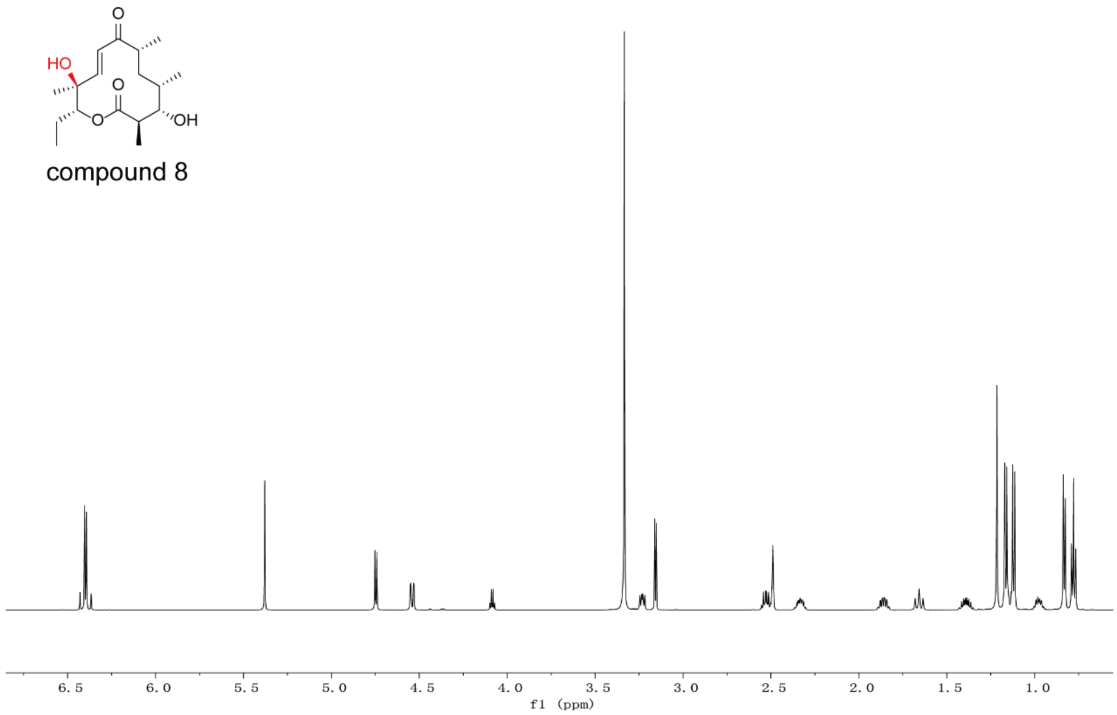

Supplementary Figure 11. ^{1}H NMR spectrum of compound **8** in DMSO-d6.

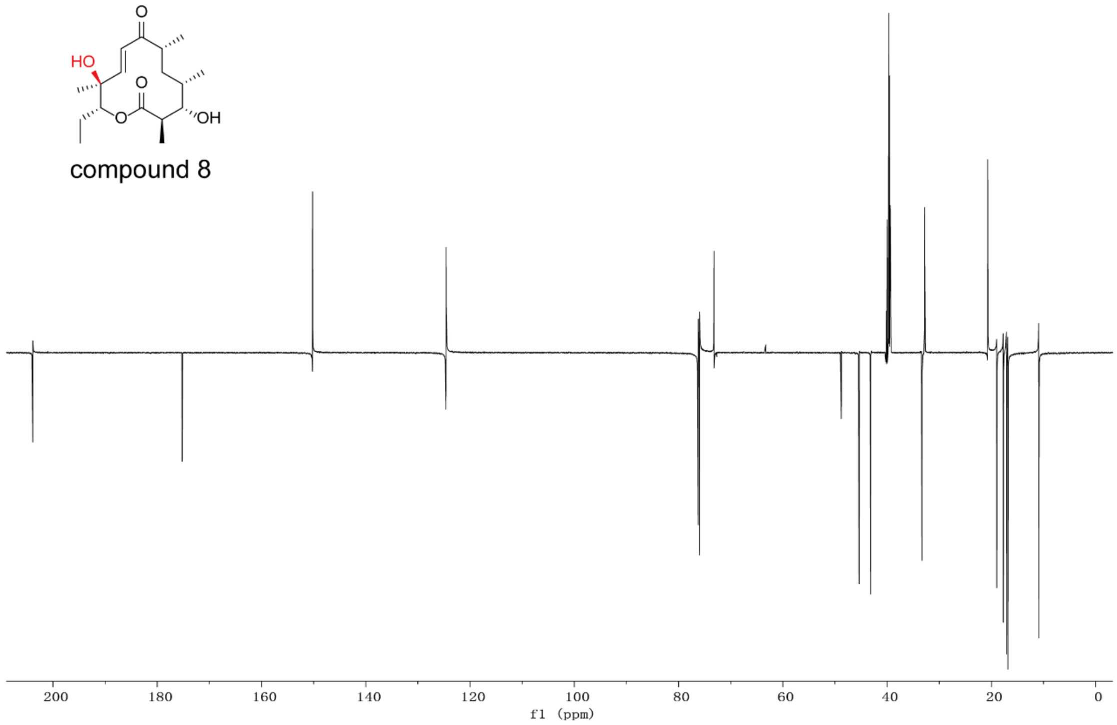

Supplementary Figure12. DEPT ^{13}C NMR spectrum of compound **8** in DMSO-d6.

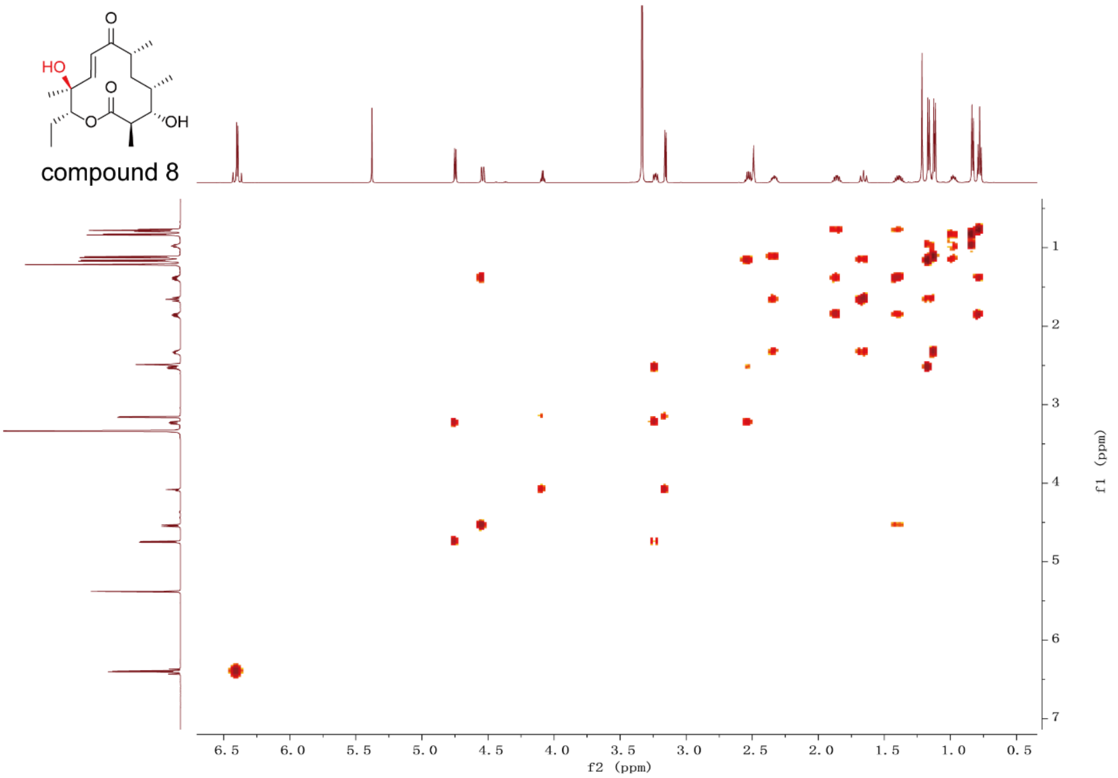

Supplementary Figure 13. ^{1}H-^{1}H COSY spectrum of compound **8** in DMSO-d6.

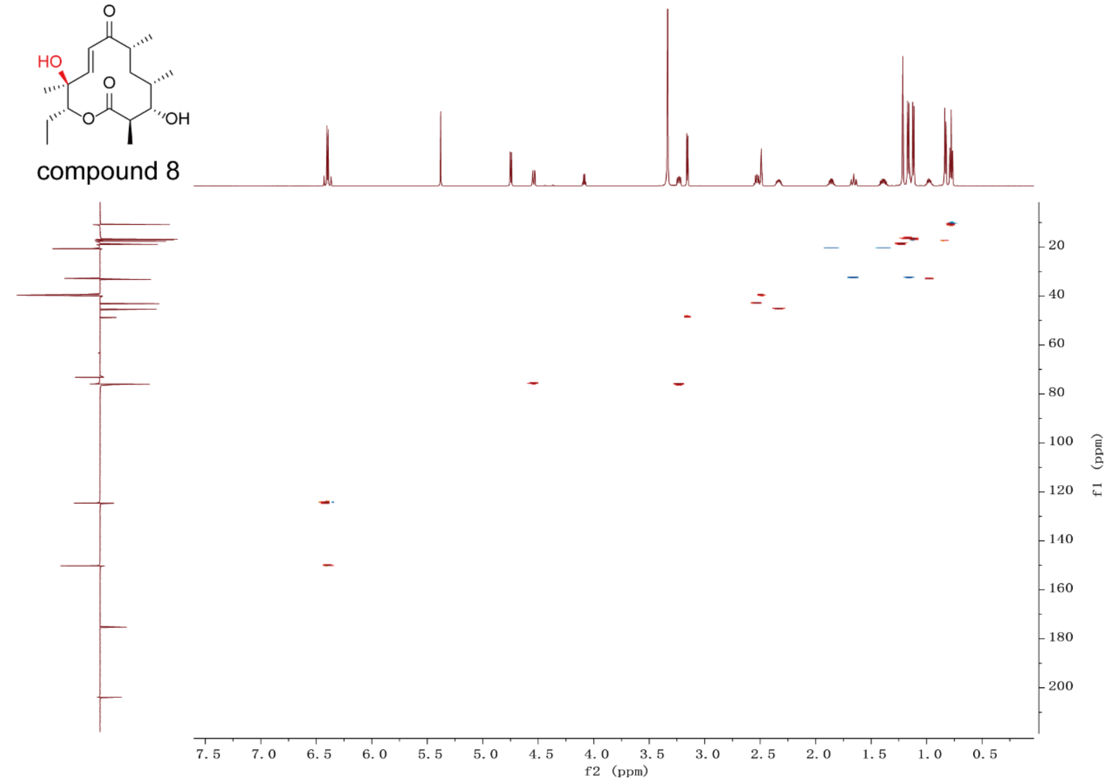

Supplementary Figure 14. HSQC spectrum of compound **8** in DMSO-d6.

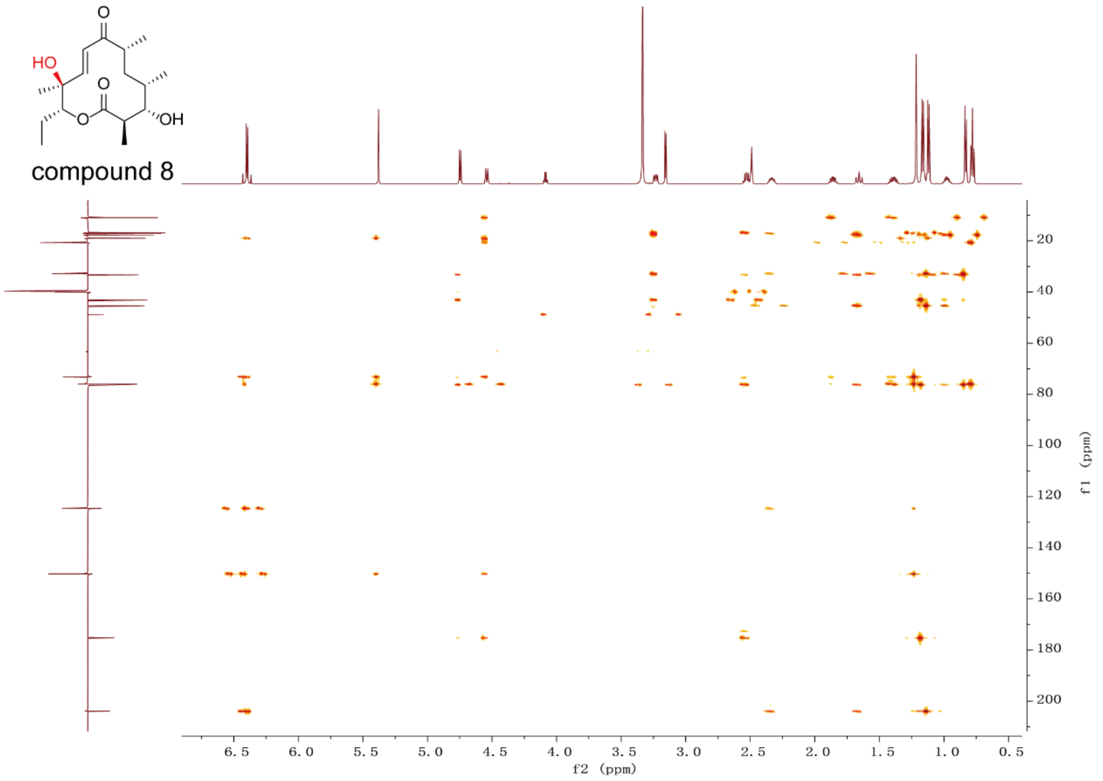

Supplementary Figure 15. HMBC spectrum of compound **8** in DMSO-d6.

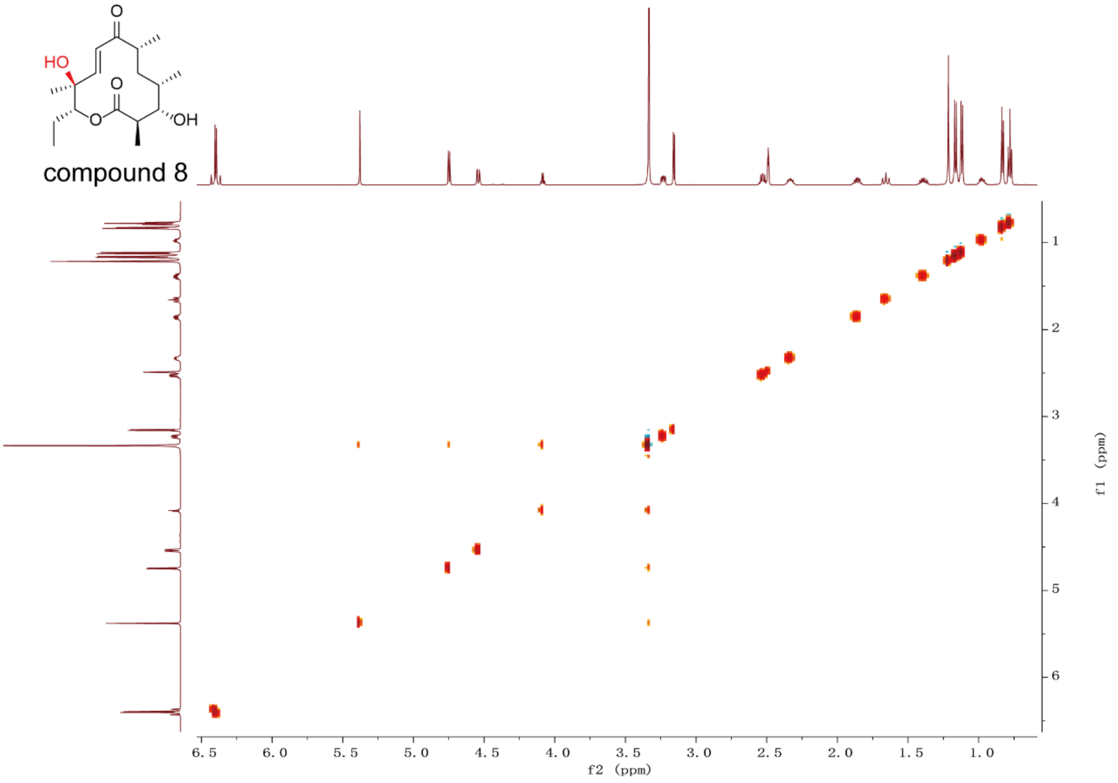

Supplementary Figure 16. NOESY spectrum of compound **8** in DMSO-d6.

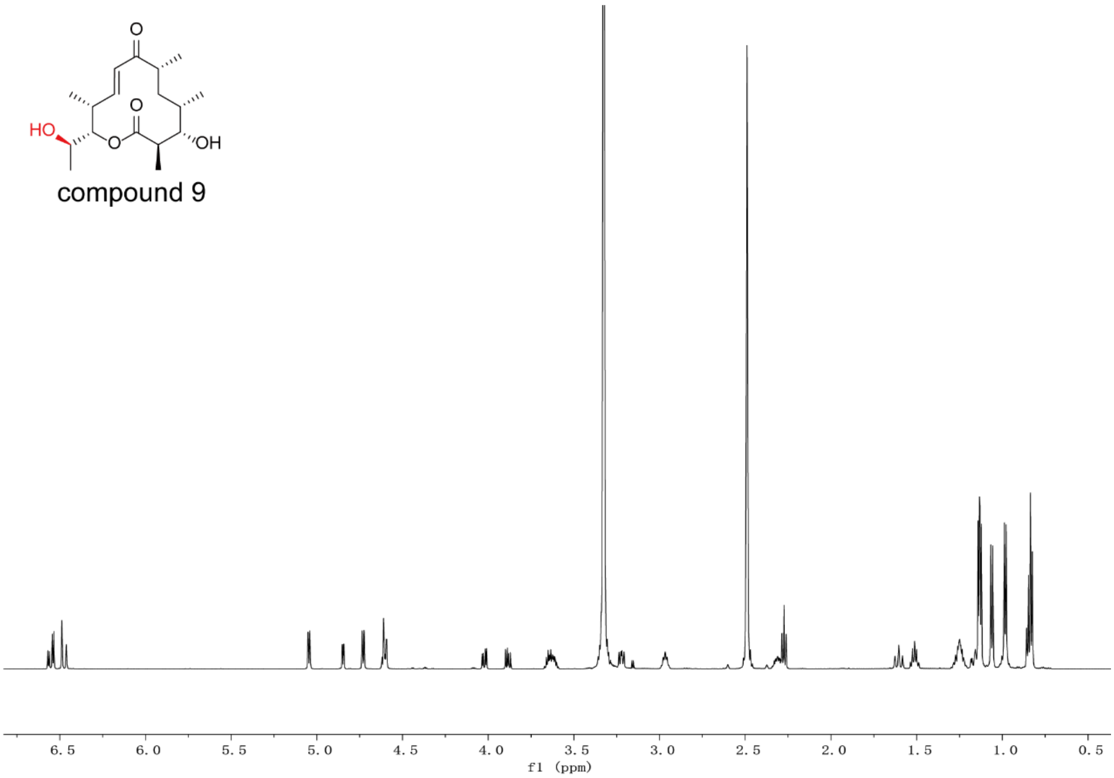

Supplementary Figure 17. ^{1}H NMR spectrum of compound **9** in DMSO-d6.

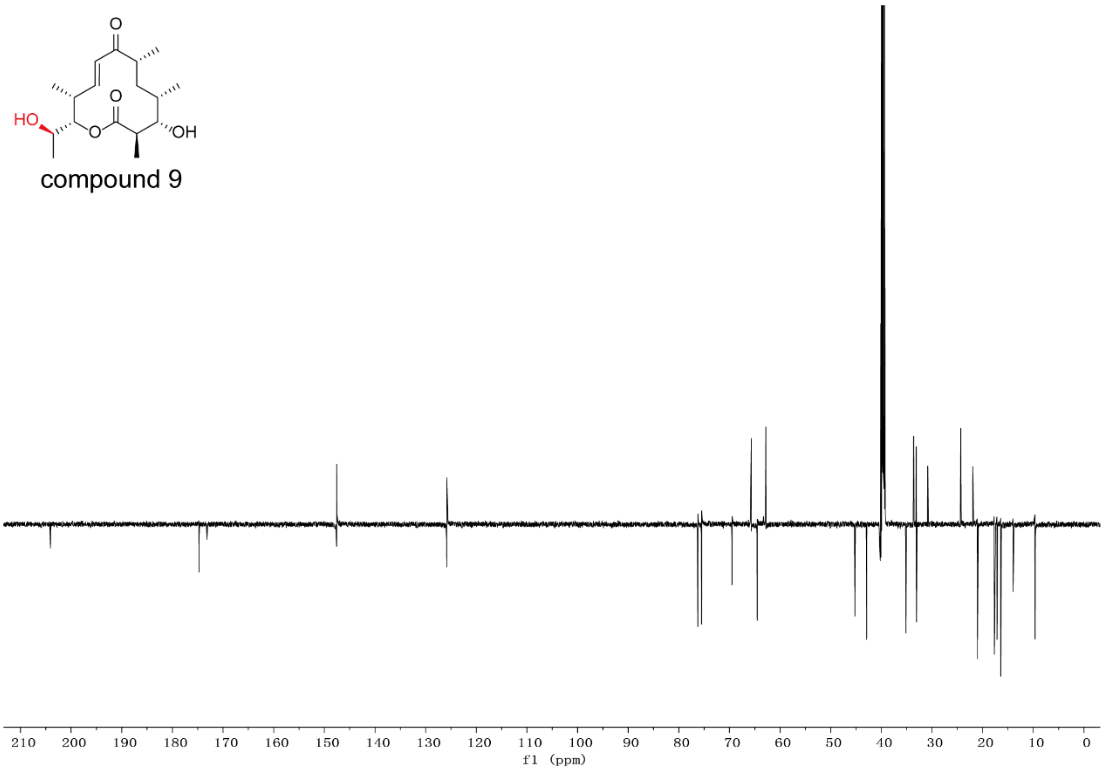

Supplementary Figure 18. DEPT ^{13}C NMR spectrum of compound **9** in DMSO-d6.

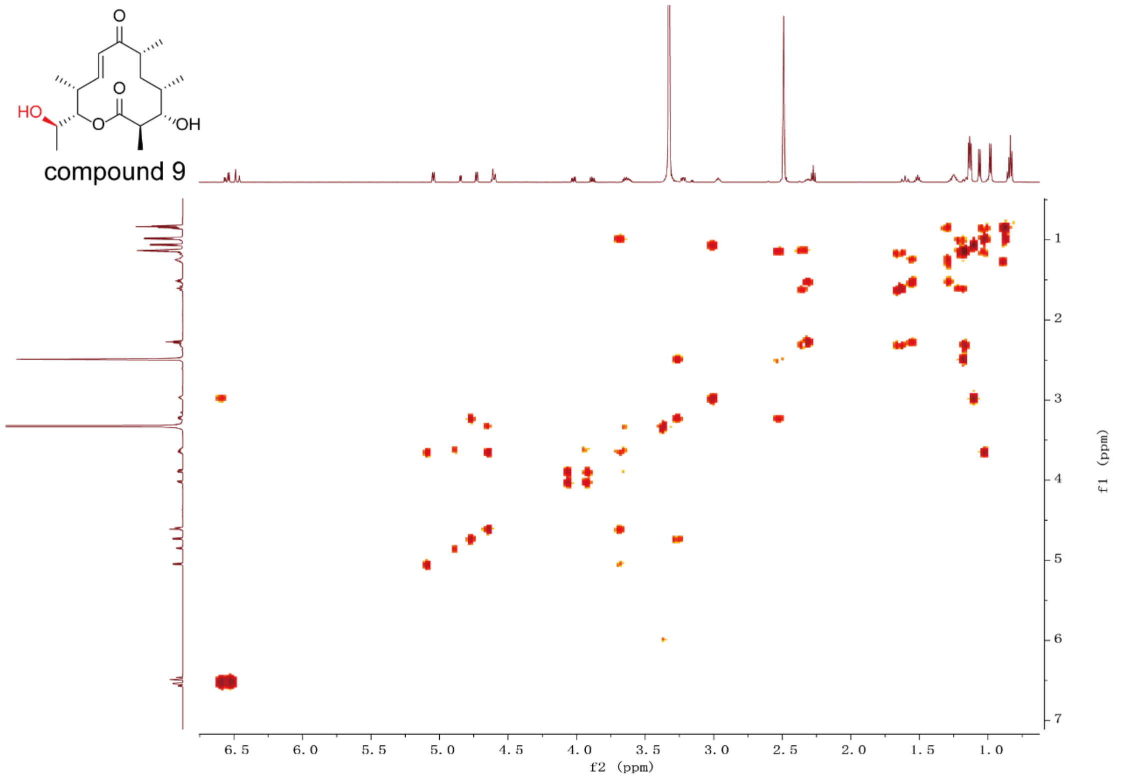

Supplementary Figure 19. ^{1}H-^{1}H COSY spectrum of compound **9** in DMSO-d6.

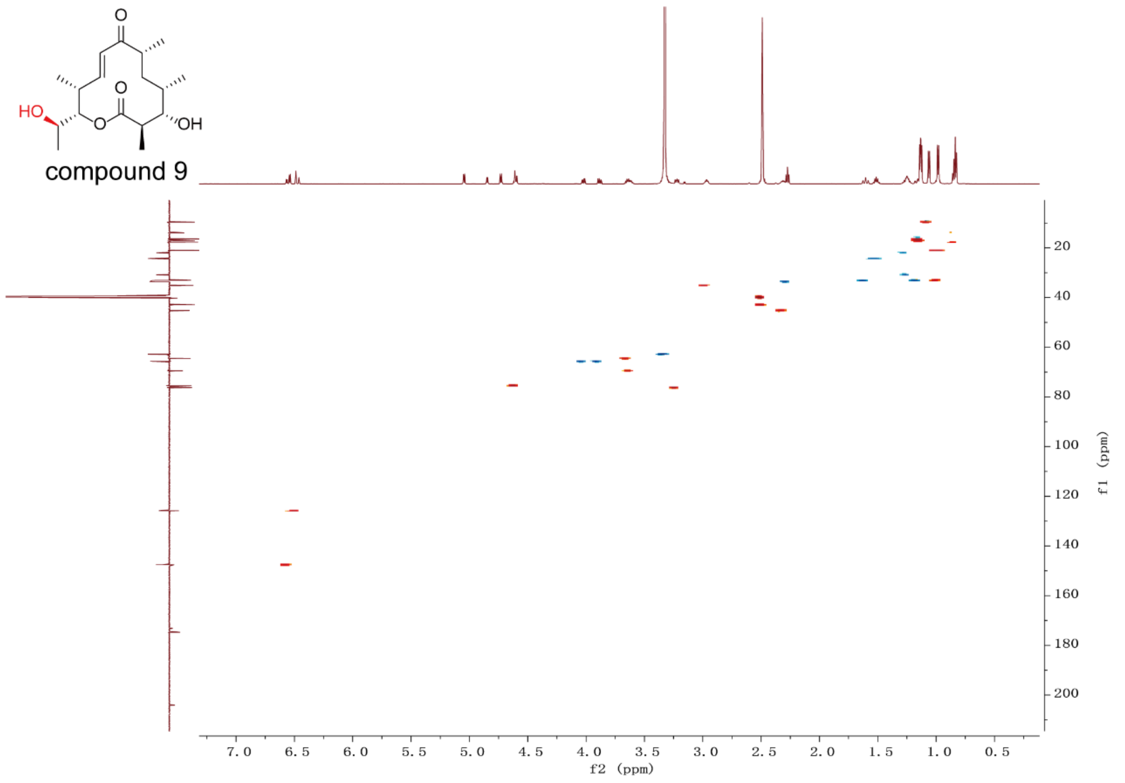

Supplementary Figure 20. HSQC spectrum of compound **9** in DMSO-d6.

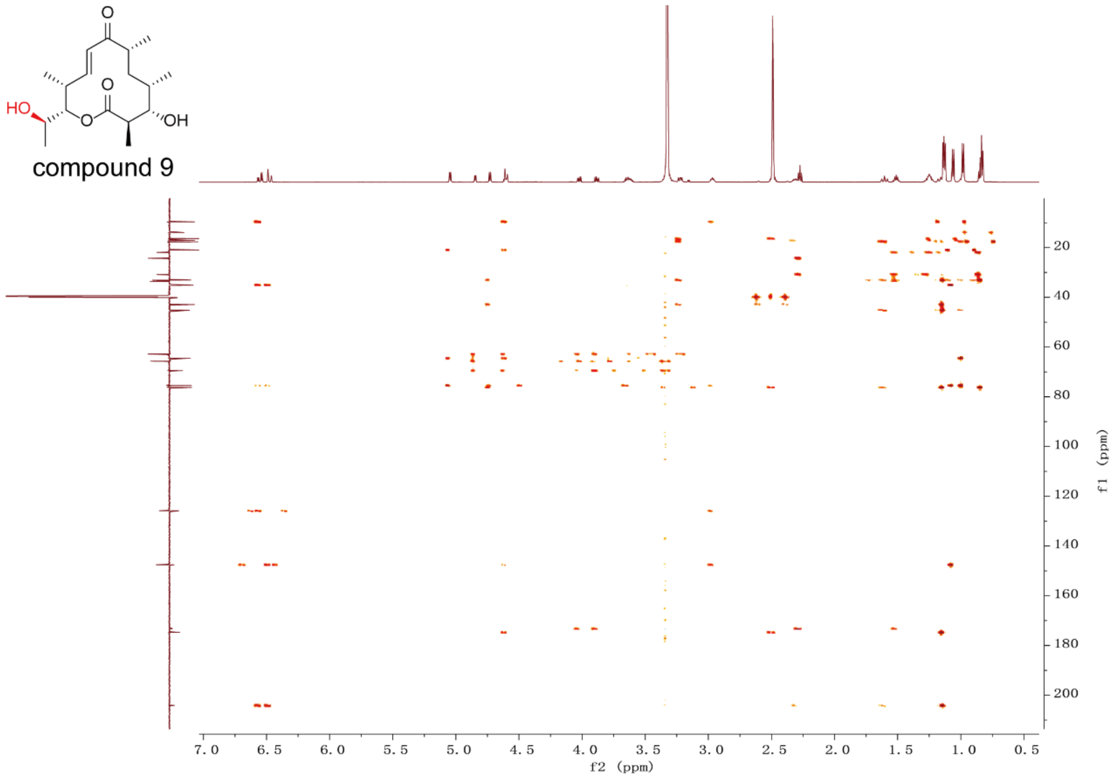

Supplementary Figure 21. HMBC spectrum of compound **9** in DMSO-d6.

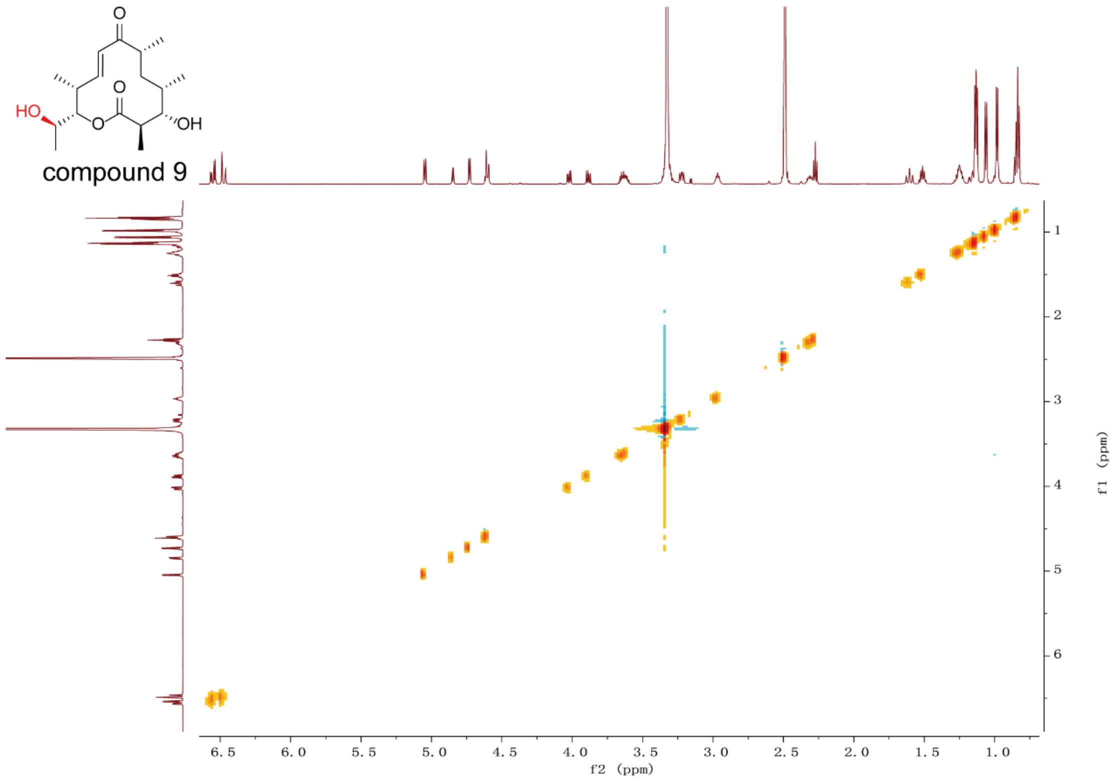

Supplementary Figure 22. NOSY spectrum of compound **9** in DMSO-d6.

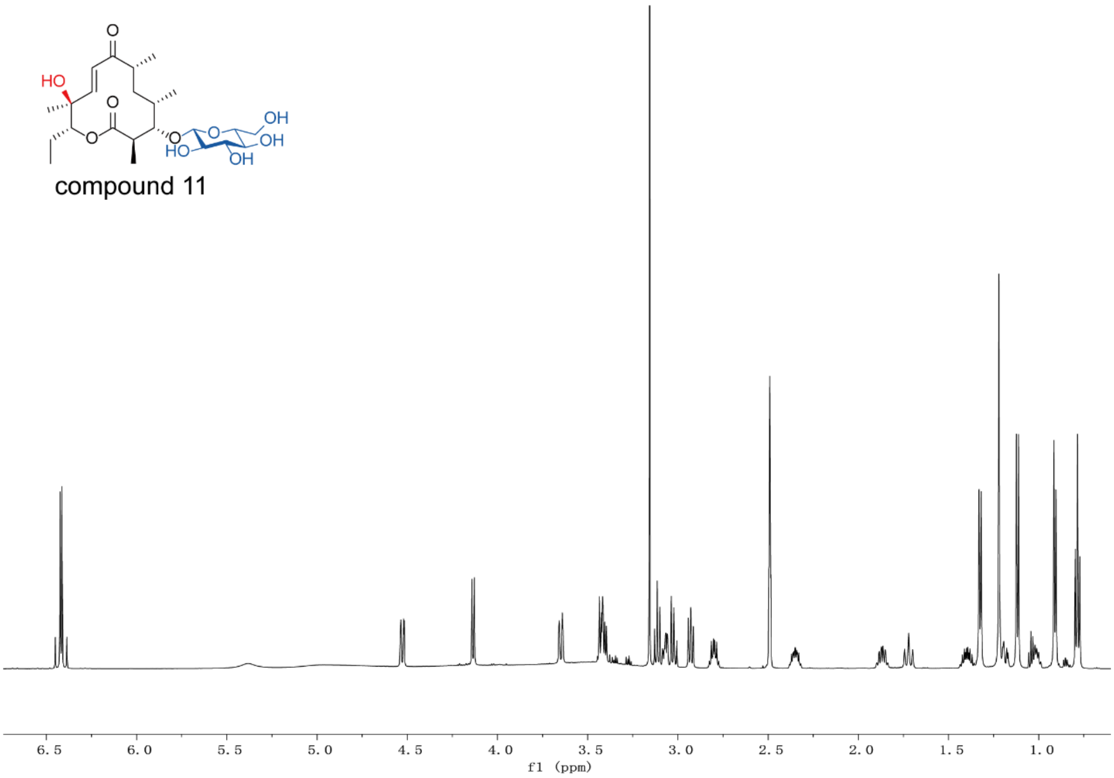

Supplementary Figure 23. ^{1}H NMR spectrum of compound **11** in DMSO-d6.

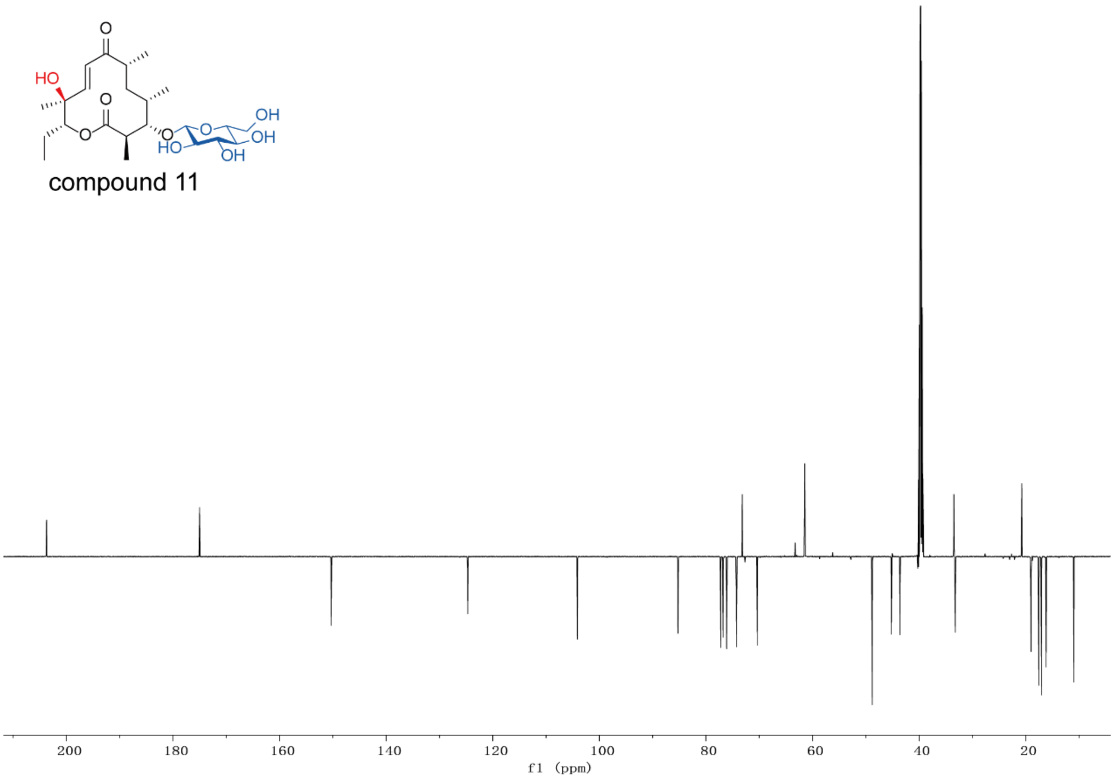

Supplementary Figure24. DEPT ^{13}C NMR spectrum of compound **11** in DMSO-d6.

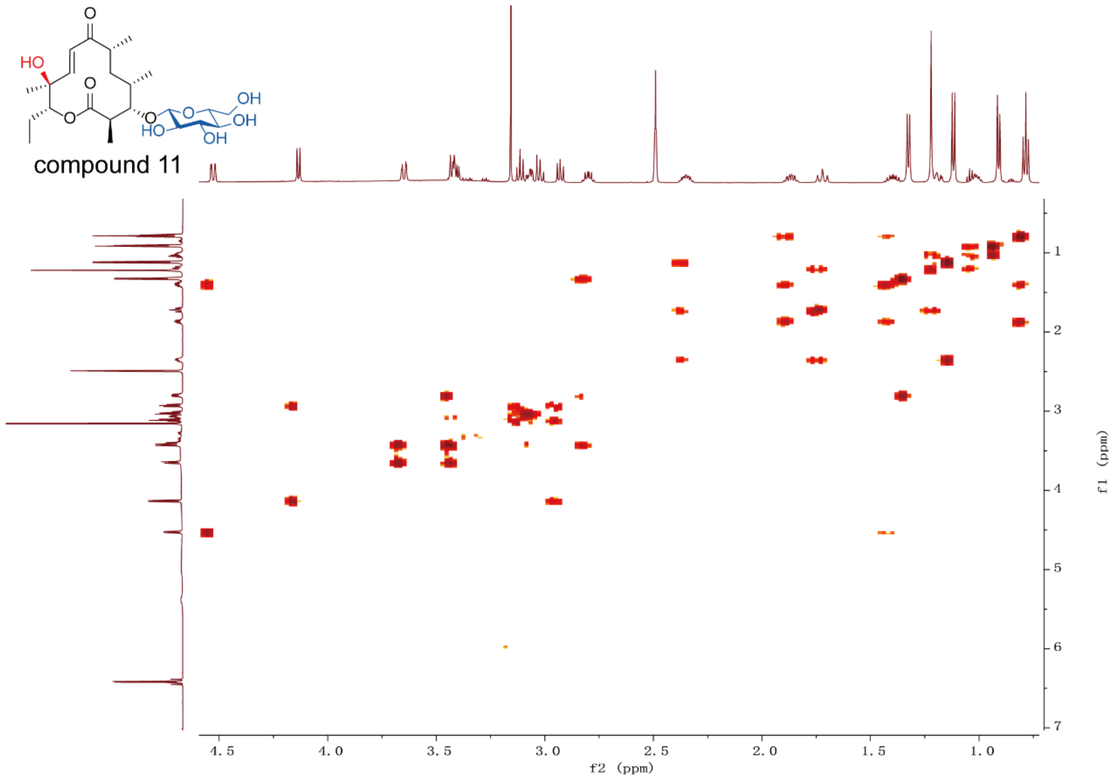

Supplementary Figure 25. ^{1}H-^{1}H COSY spectrum of compound **11** in DMSO-d6.

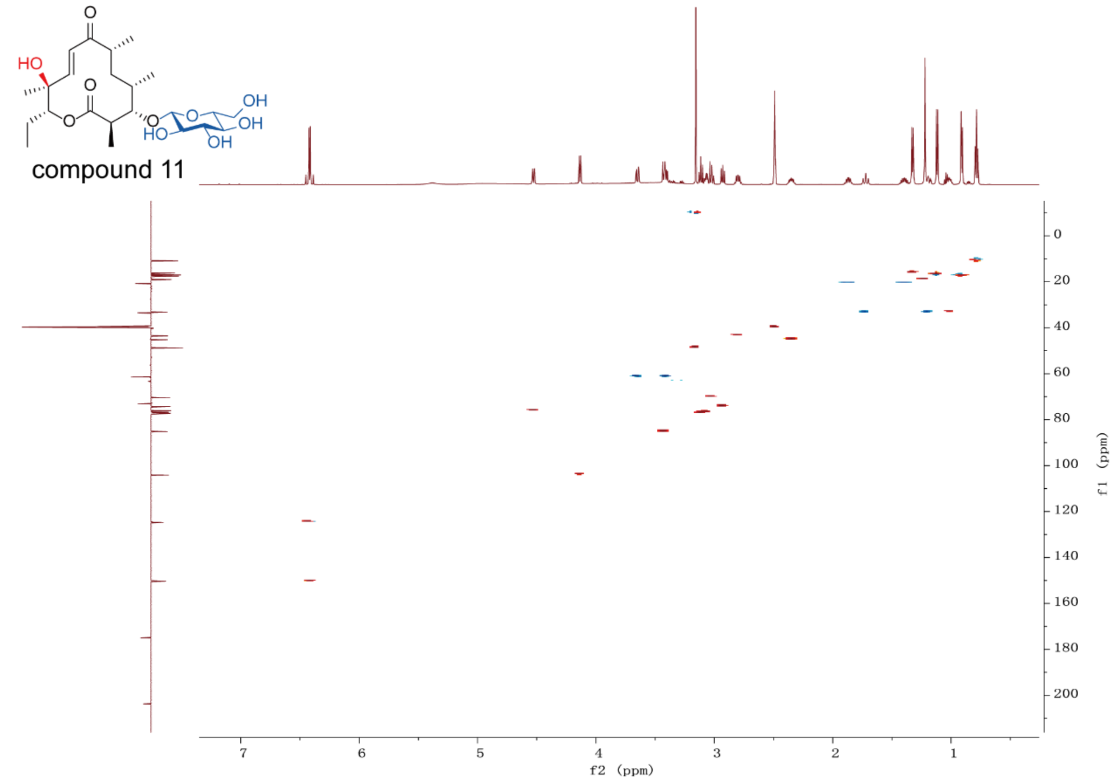

Supplementary Figure 26. HSQC spectrum of compound **11** in DMSO-d6.

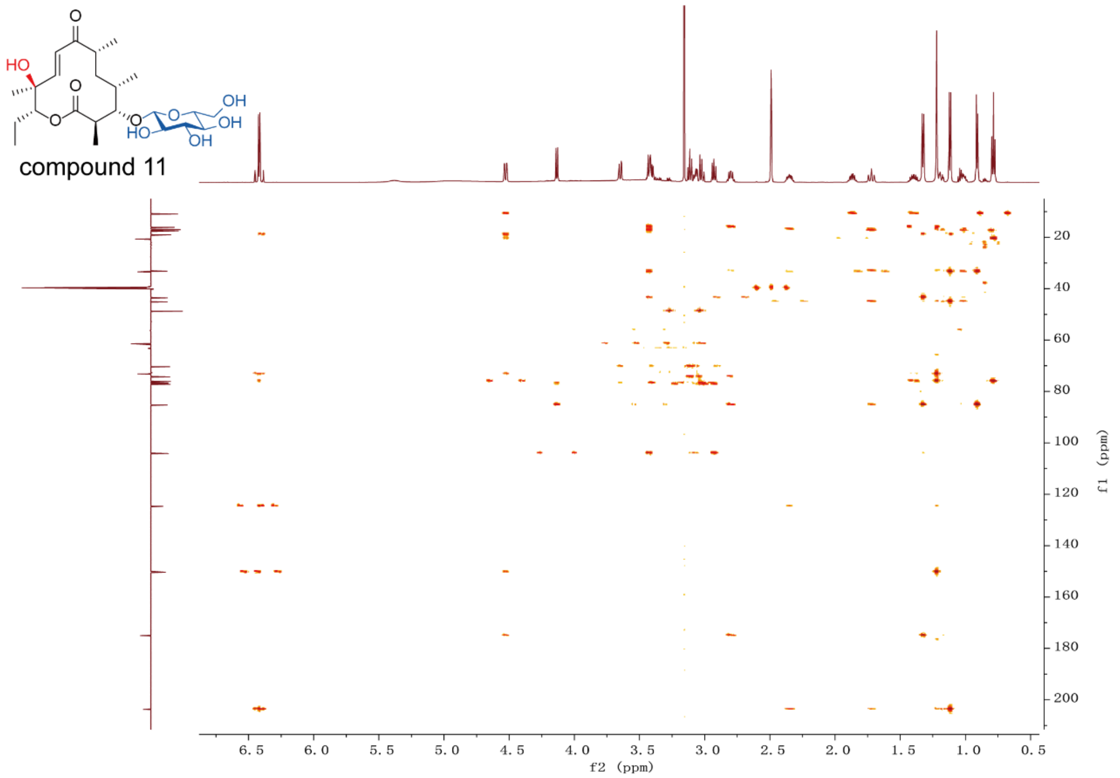

Supplementary Figure 27. HMBC spectrum of compound **11** in DMSO-d6.

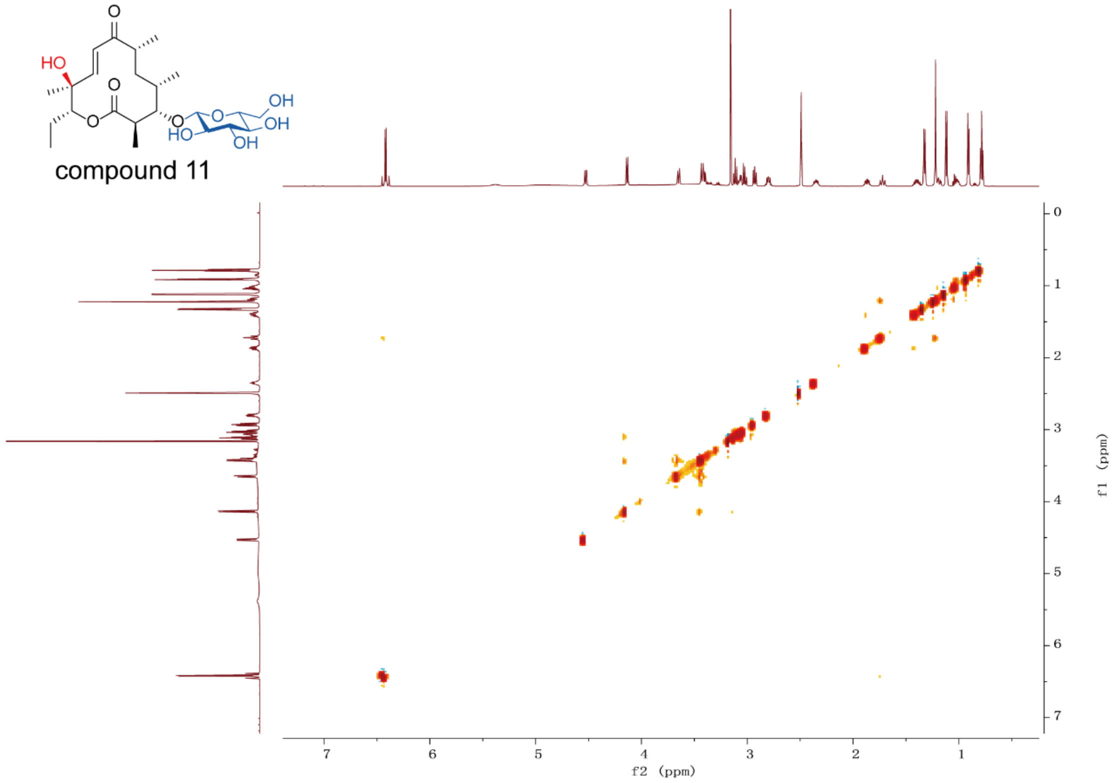

Supplementary Figure 28. NOESY spectrum of compound **11** in DMSO-d6.

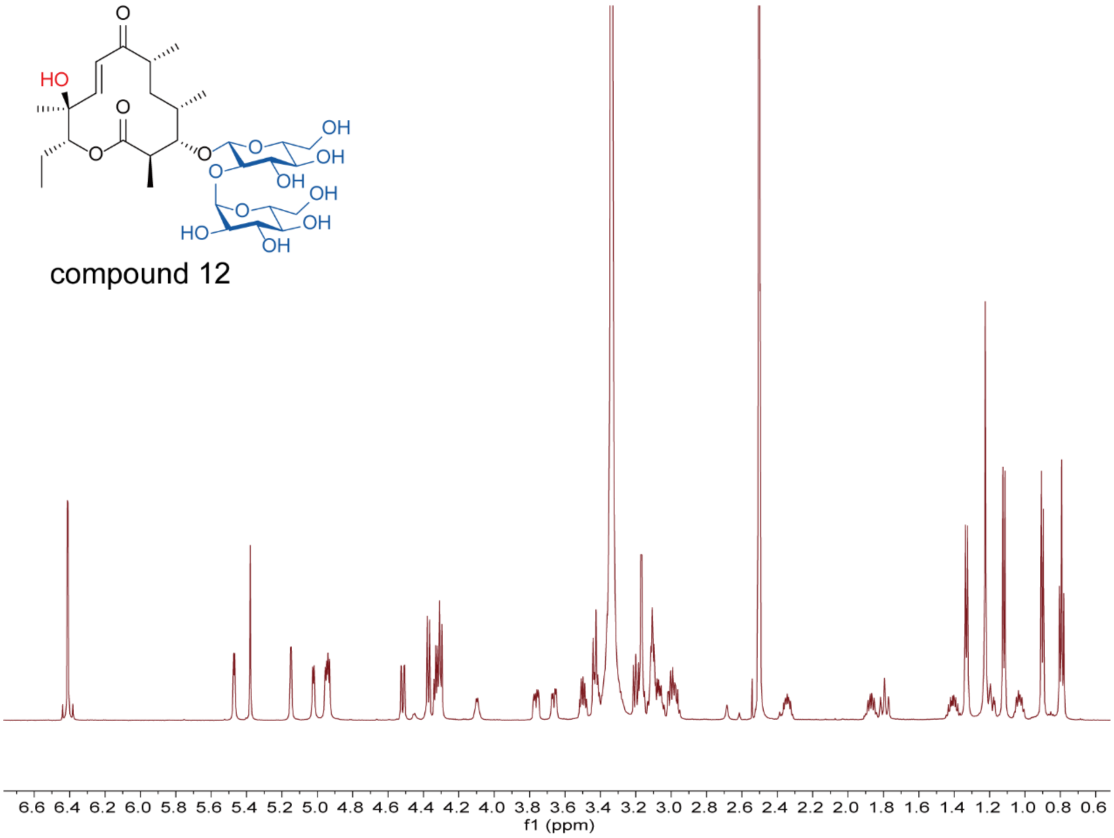

Supplementary Figure 29. ^{1}H NMR spectrum of compound **12** in DMSO-d6.

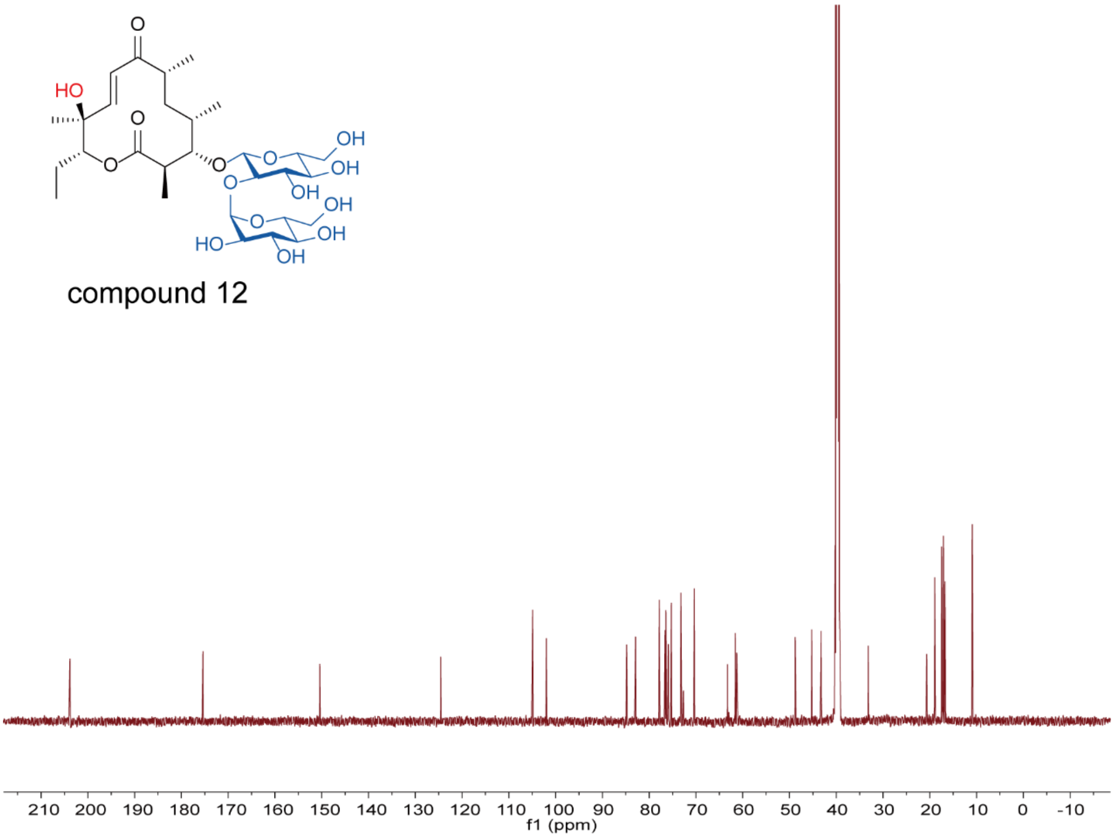

Supplementary Figure 30. ^{13}C NMR spectrum of compound **12** in DMSO-d6.

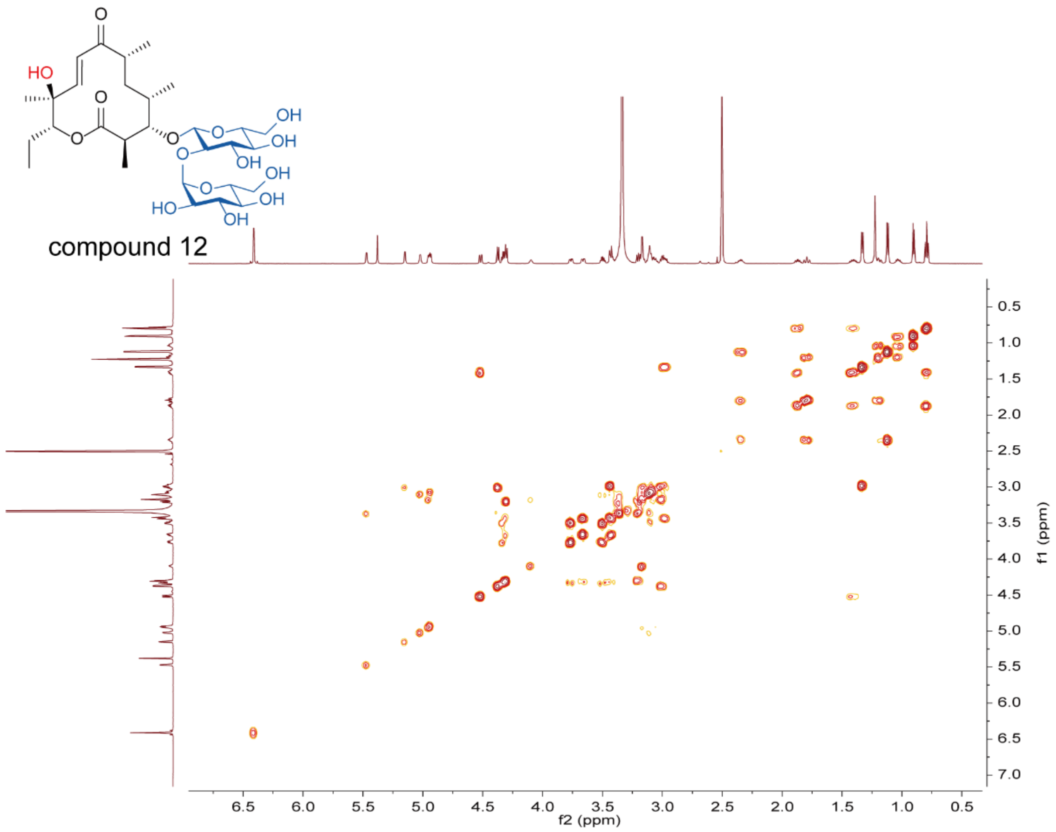

Supplementary Figure 31. ^{1}H-^{1}H COSY spectrum of compound **12** in DMSO-d6.

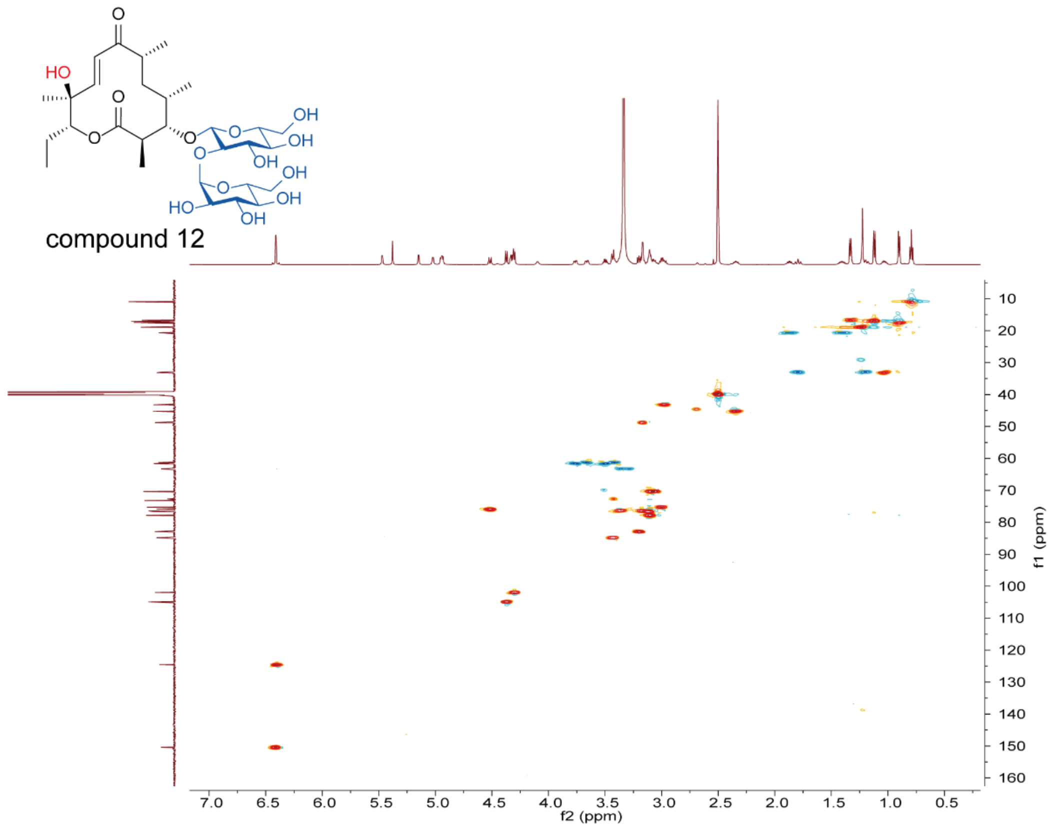

Supplementary Figure 32. HSQC spectrum of compound **12** in DMSO-d6.

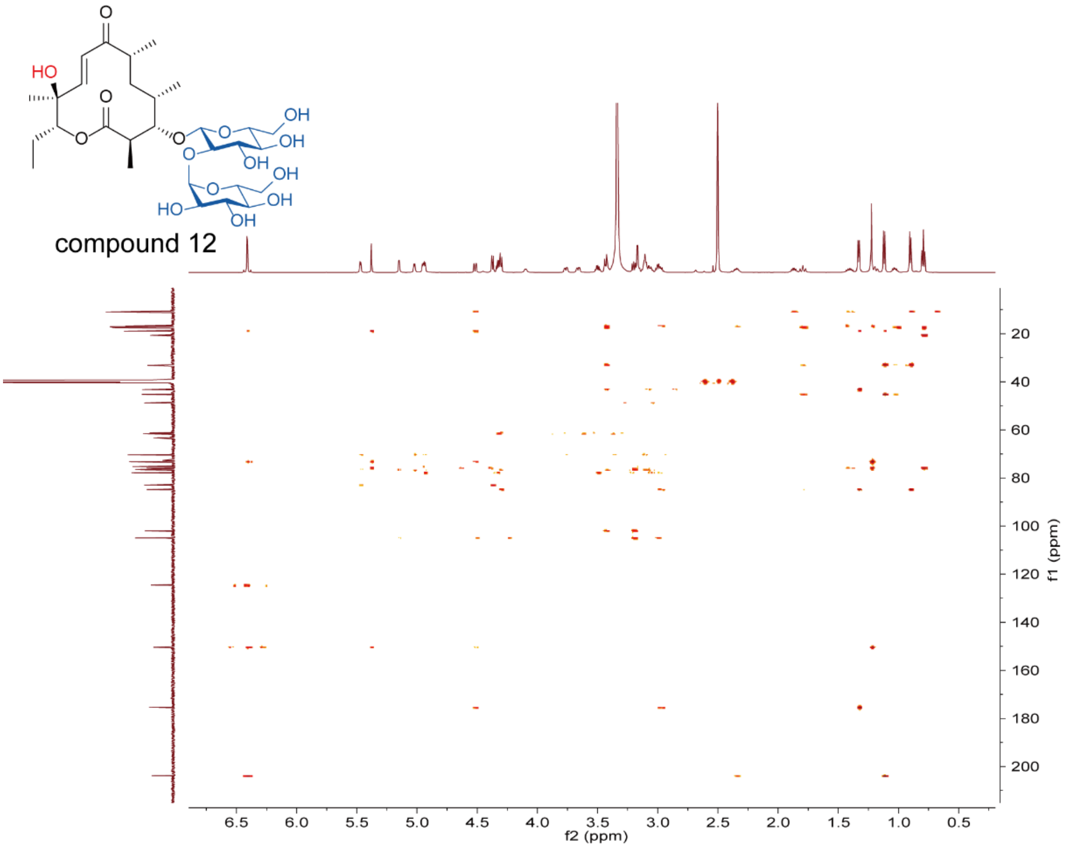

Supplementary Figure 33. HMBC spectrum of compound **12** in DMSO-d6.

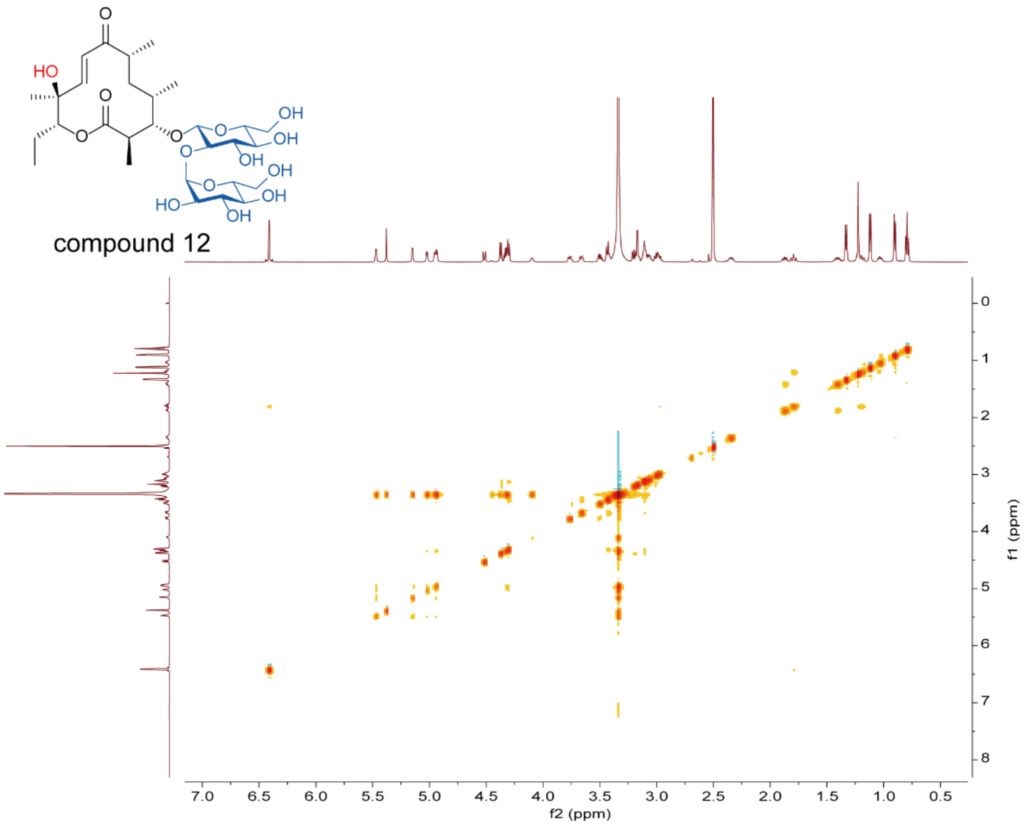

Supplementary Figure 34. NOESY spectrum of compound **12** in DMSO-d6.

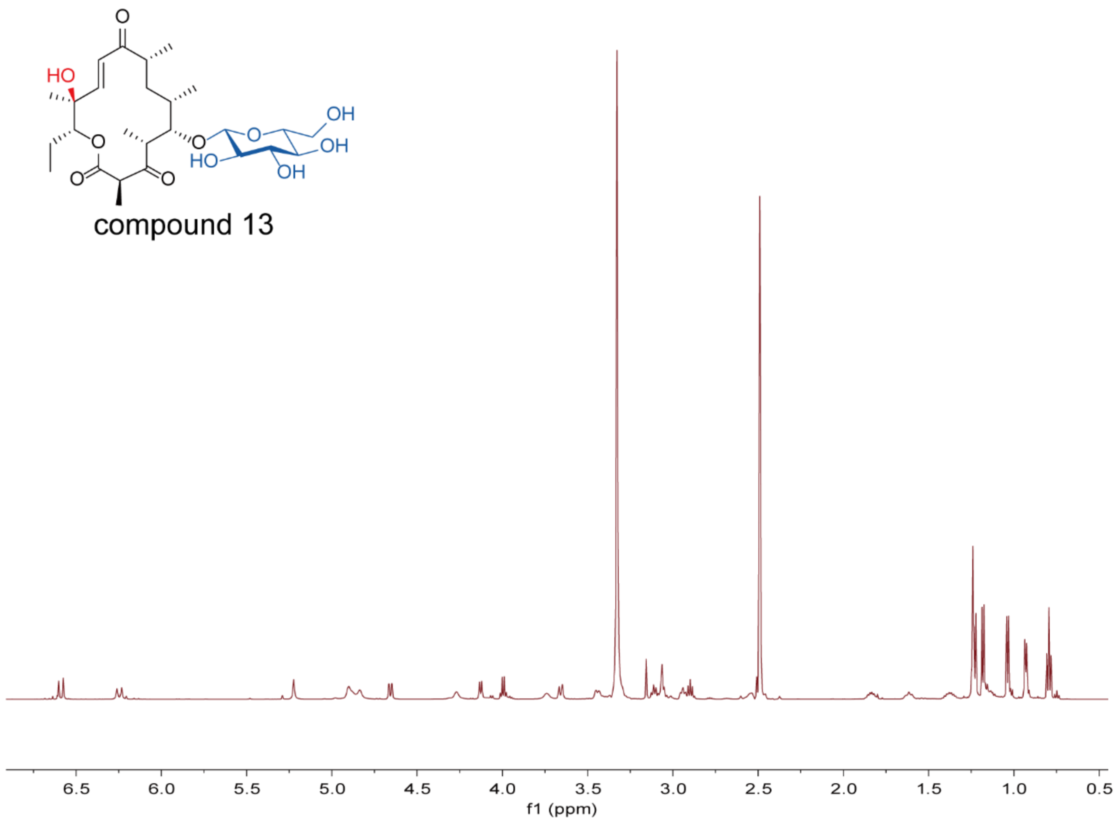

Supplementary Figure 35. ^{1}H NMR spectrum of compound **13** in DMSO-d6.

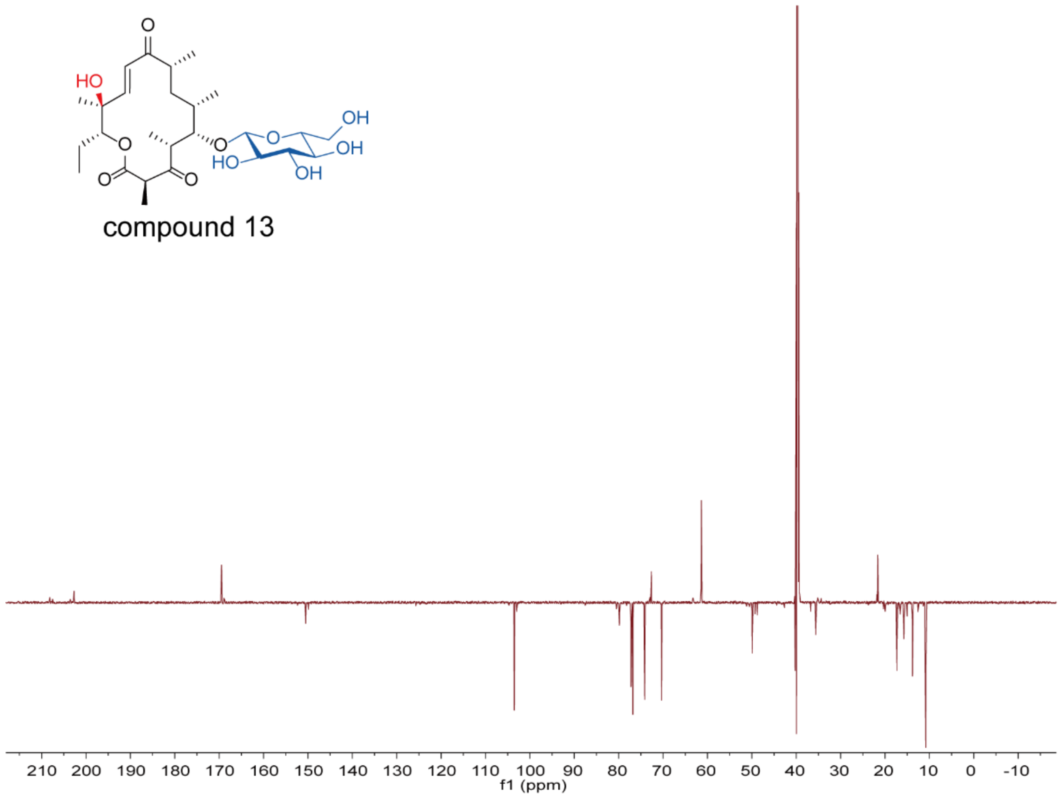

Supplementary Figure 36. DEPT ^{13}C NMR spectrum of compound **13** in DMSO-d6.

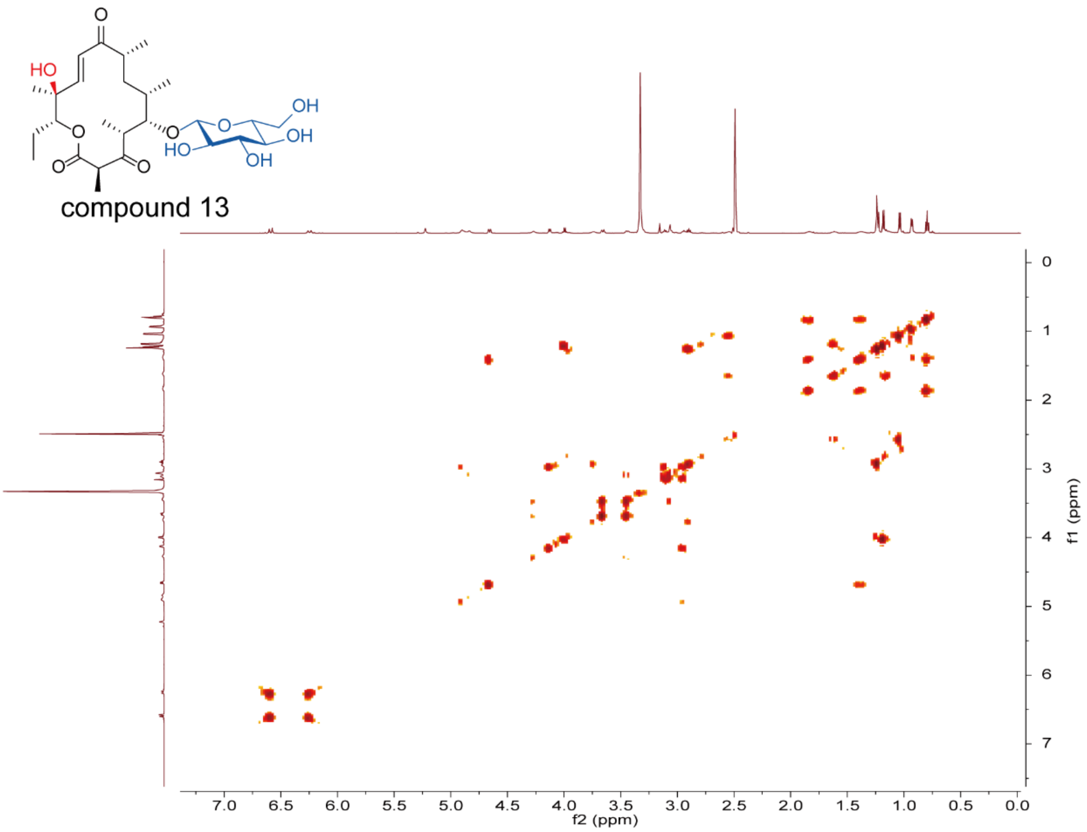

Supplementary Figure 37. ^{1}H-^{1}H COSY spectrum of compound **13** in DMSO-d6.

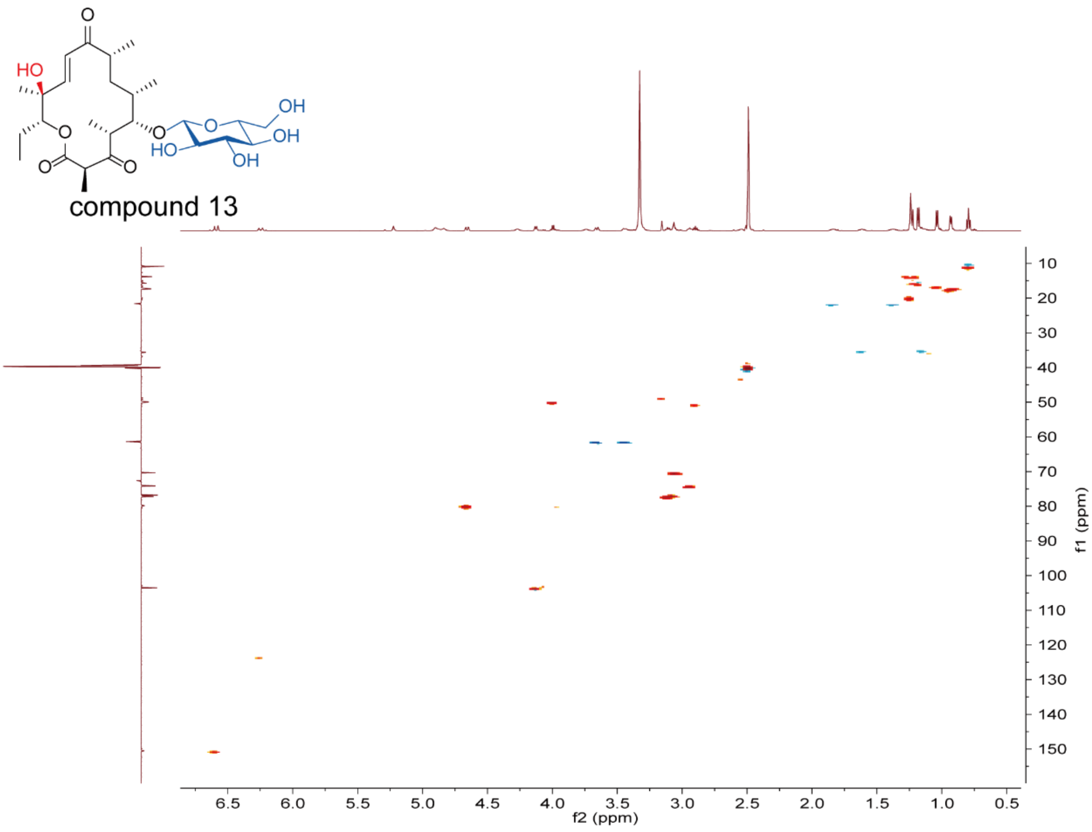

Supplementary Figure 38. HSQC spectrum of compound **13** in DMSO-d6.

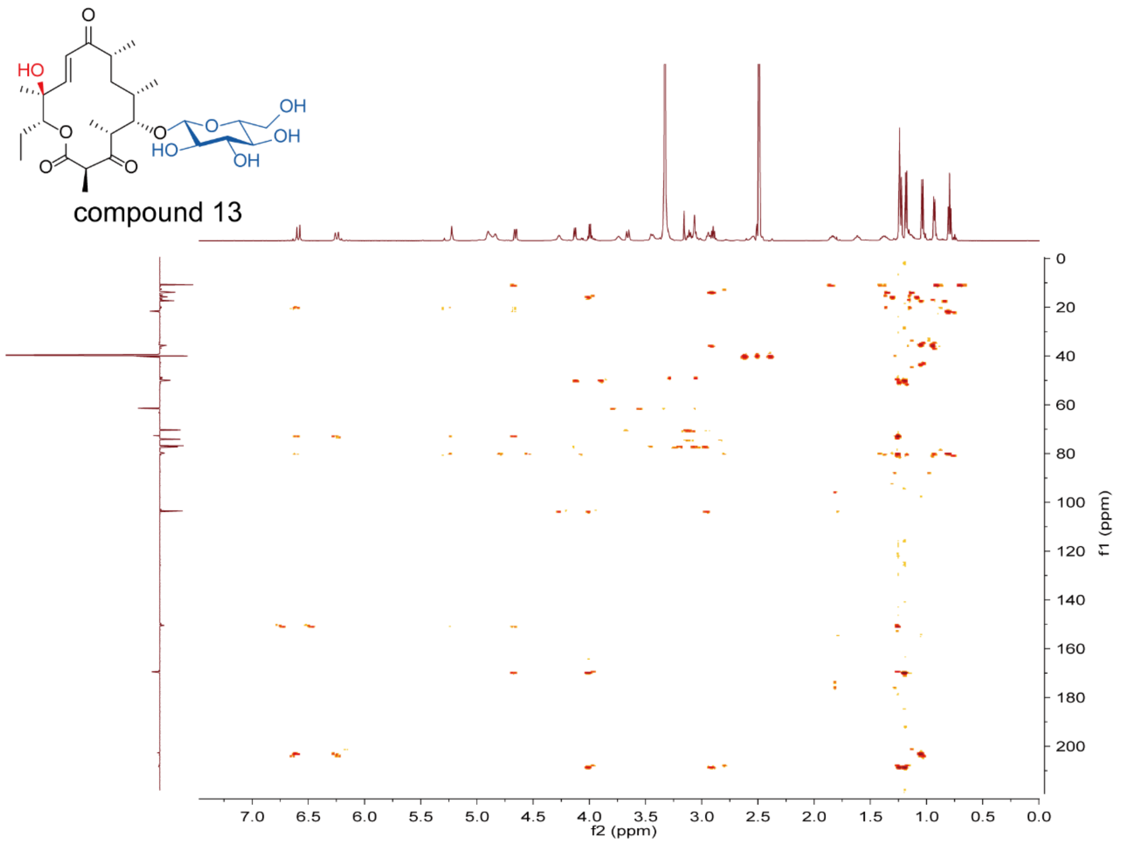

Supplementary Figure 39. HMBC spectrum of compound **13** in DMSO-d6.

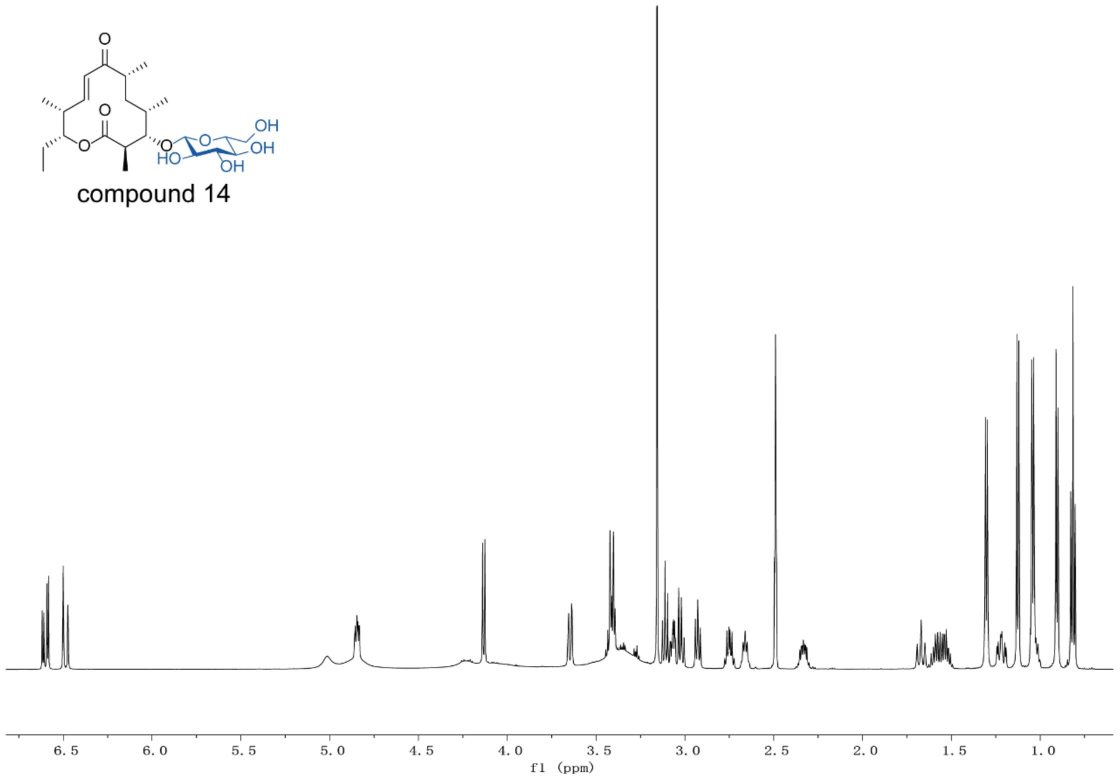

Supplementary Figure 40. ^{1}H NMR spectrum of compound **14** in DMSO-d6.

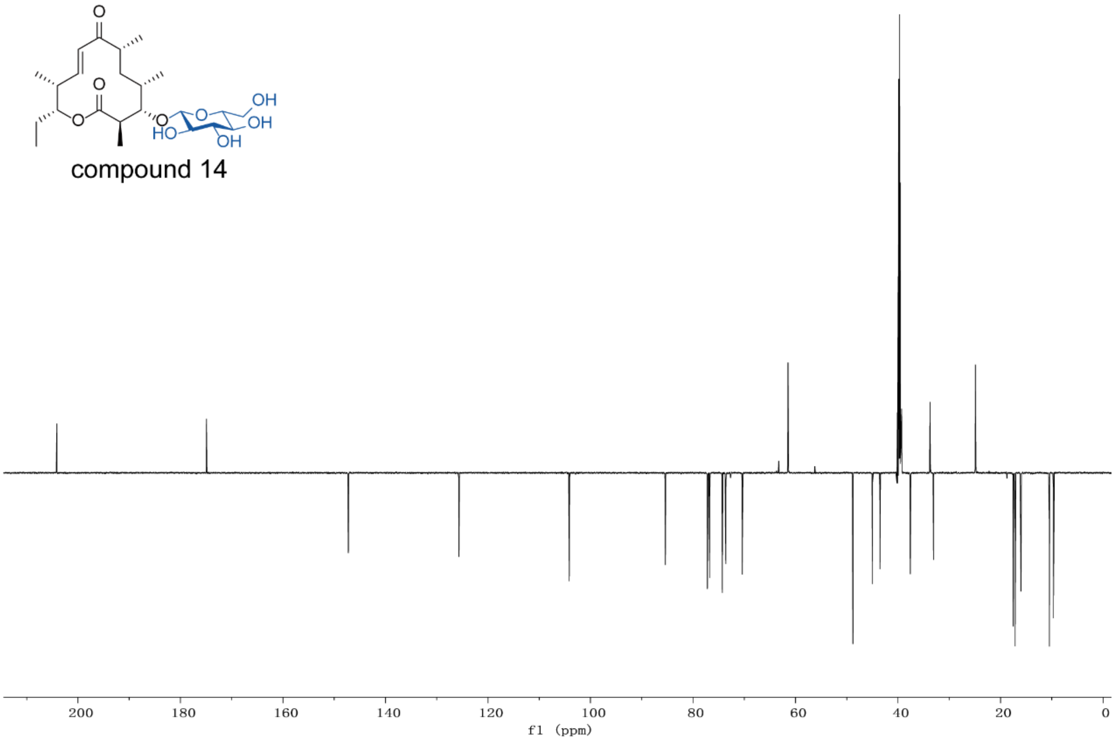

Supplementary Figure 41. DEPT ^{13}C NMR spectrum of compound **14** in DMSO-d6.

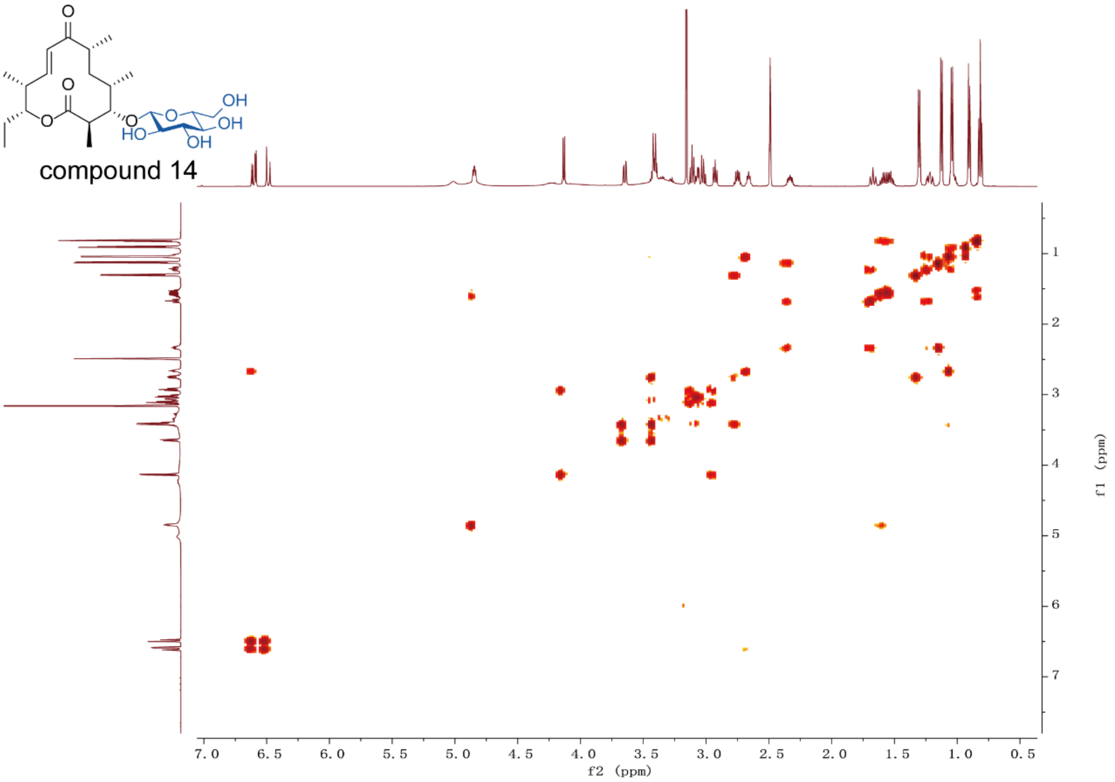

Supplementary Figure 42. ^{1}H-^{1}H COSY spectrum of compound **14** in DMSO-d6.

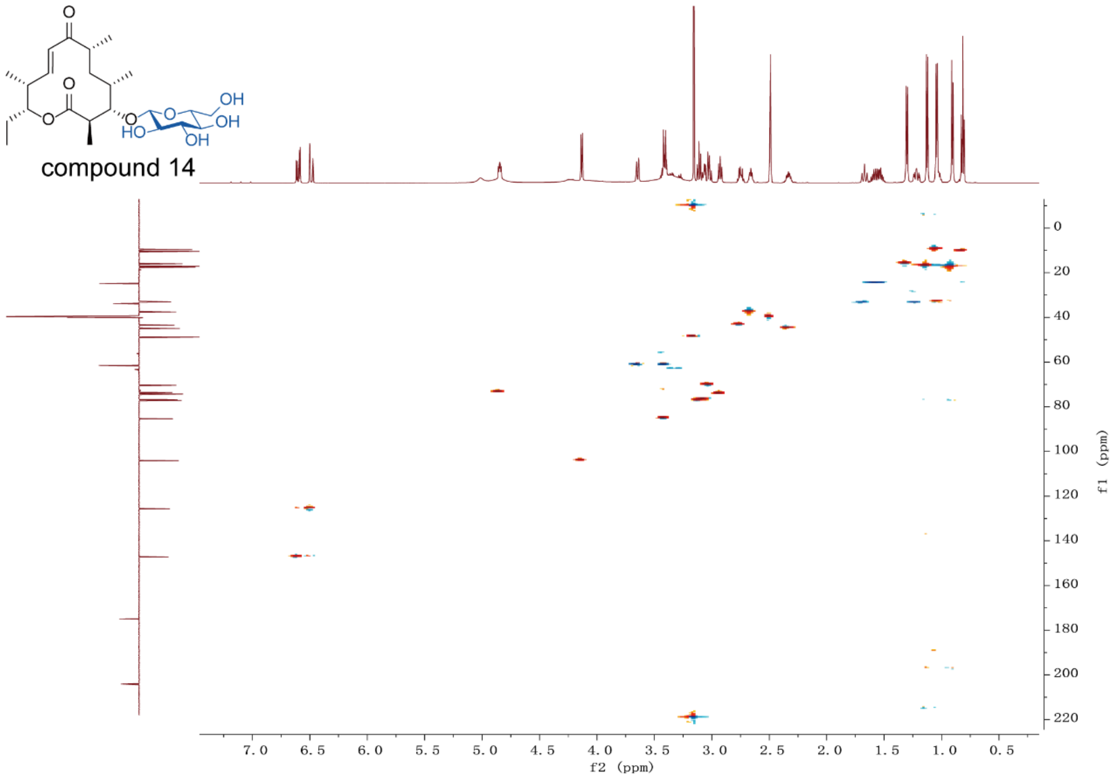

Supplementary Figure 43. HSQC spectrum of compound **14** in DMSO-d6.

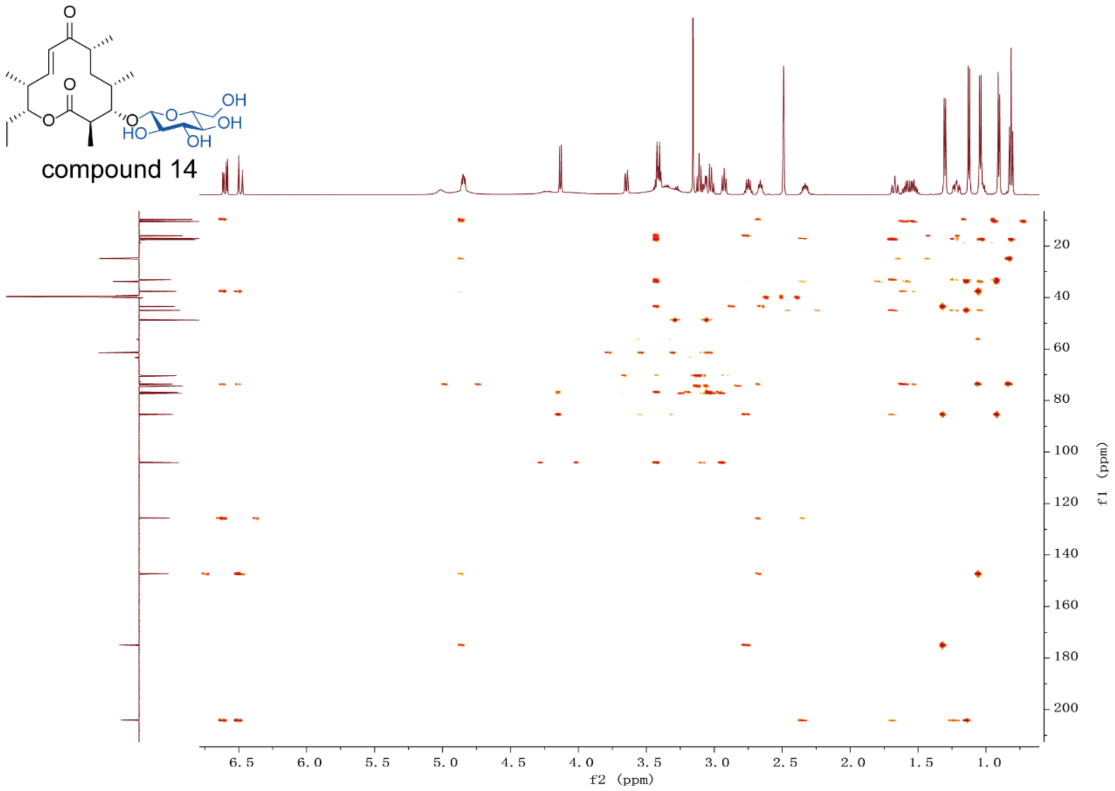

Supplementary Figure 44. HMBC spectrum of compound **14** in DMSO-d6.

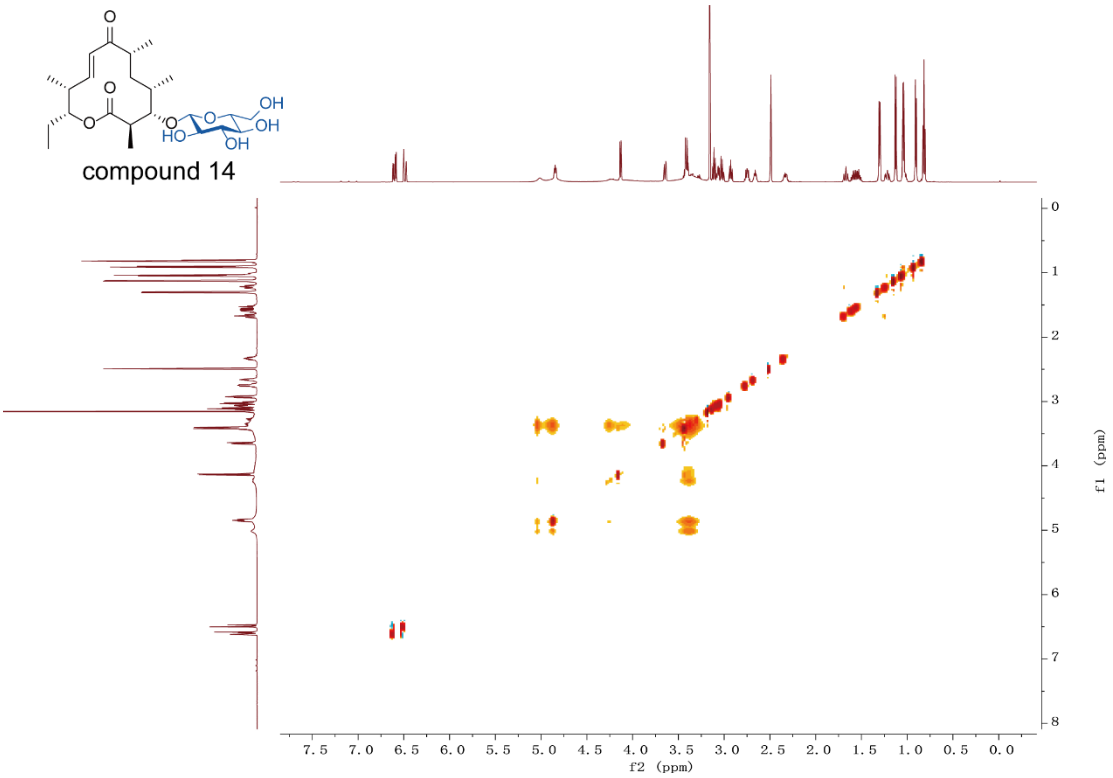

Supplementary Figure 45. NOESY spectrum of compound **14** in DMSO-d6.

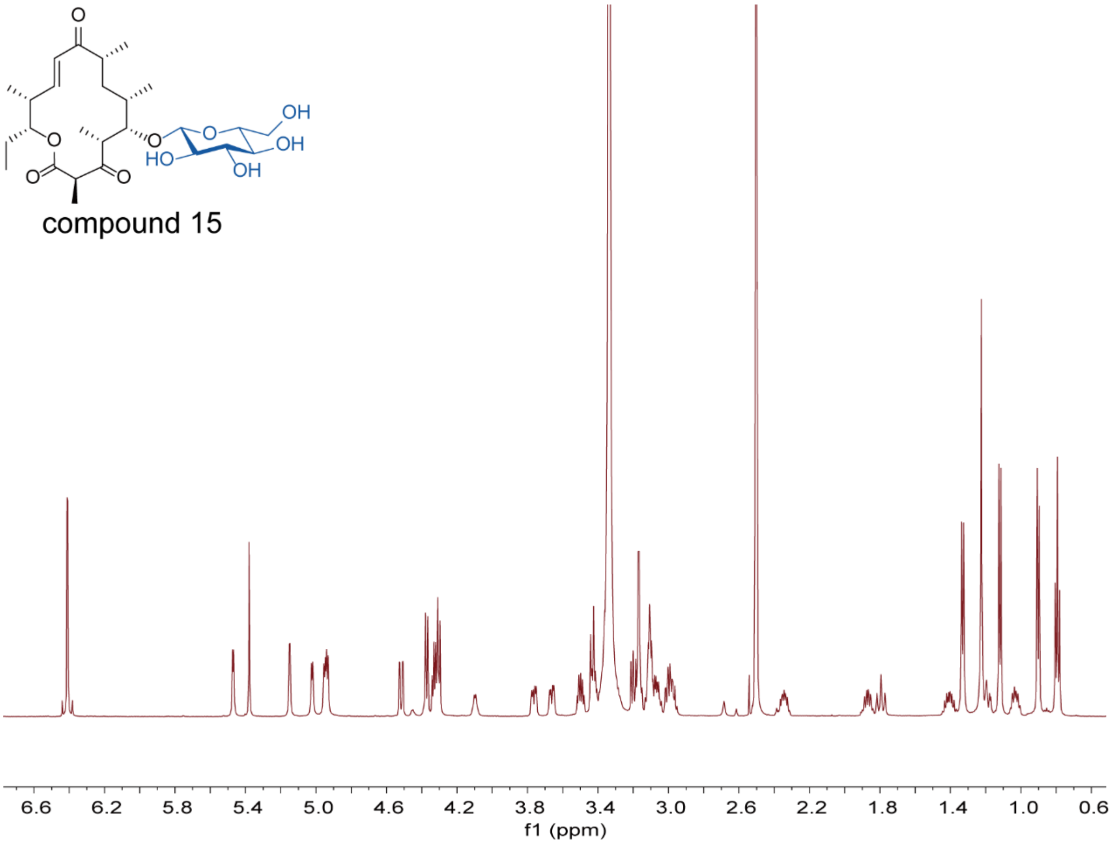

Supplementary Figure 46. ^{1}H NMR spectrum of compound **15** in DMSO-d6.

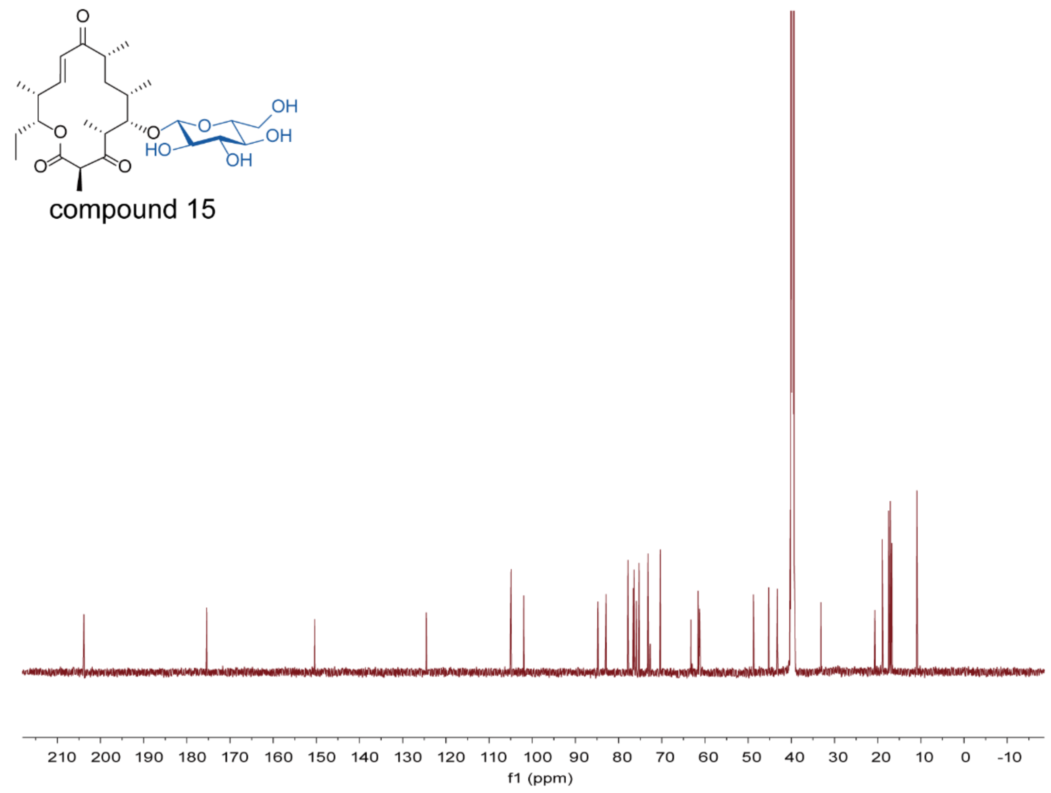

Supplementary Figure 47. ^{13}C NMR spectrum of compound **15** in DMSO-d6.

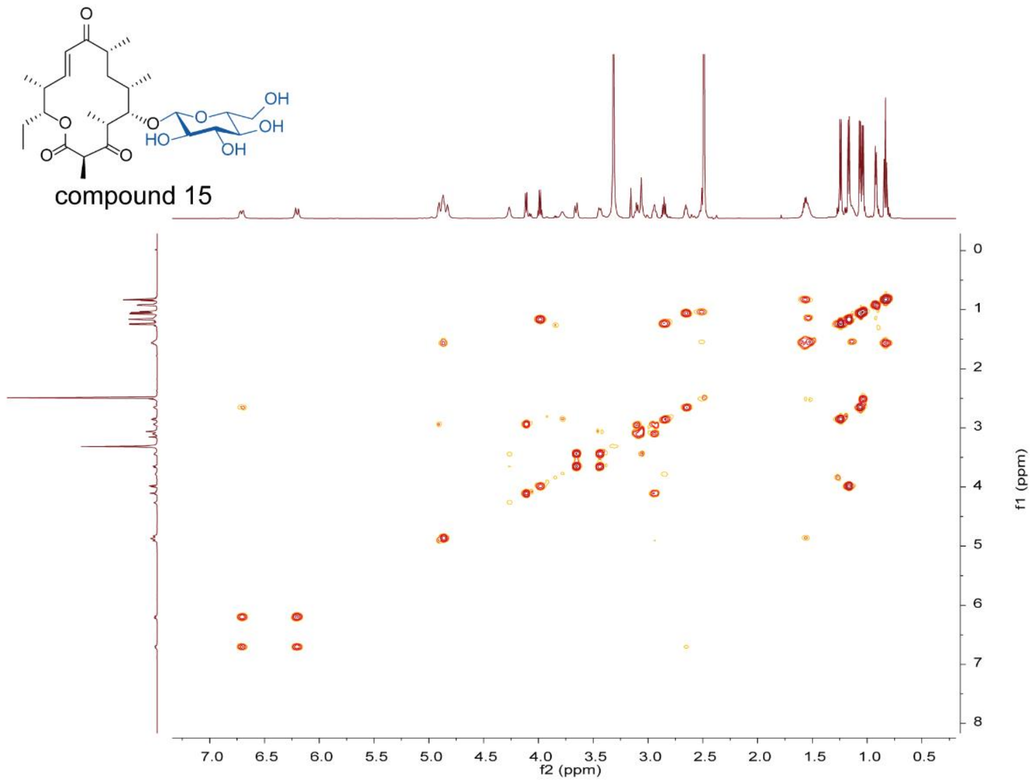

Supplementary Figure 48. ^{1}H-^{1}H COSY spectrum of compound **15** in DMSO-d6.

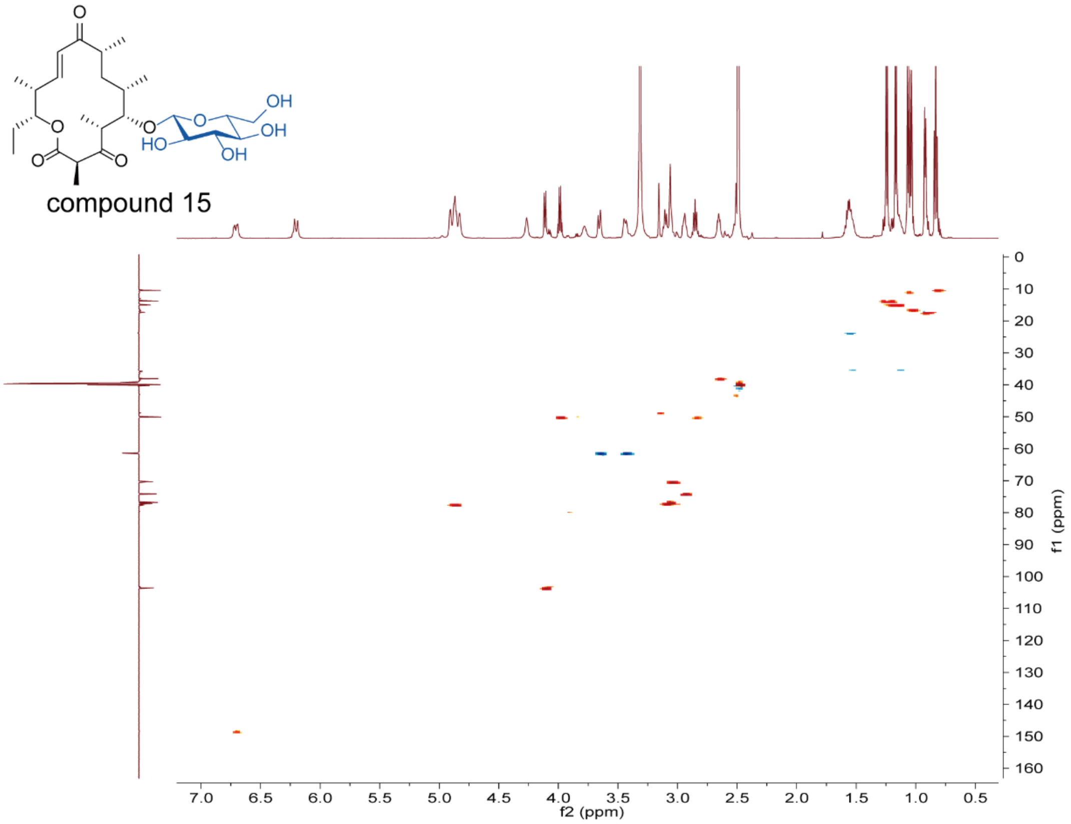

Supplementary Figure 49. HSQC spectrum of compound **15** in DMSO-d6.

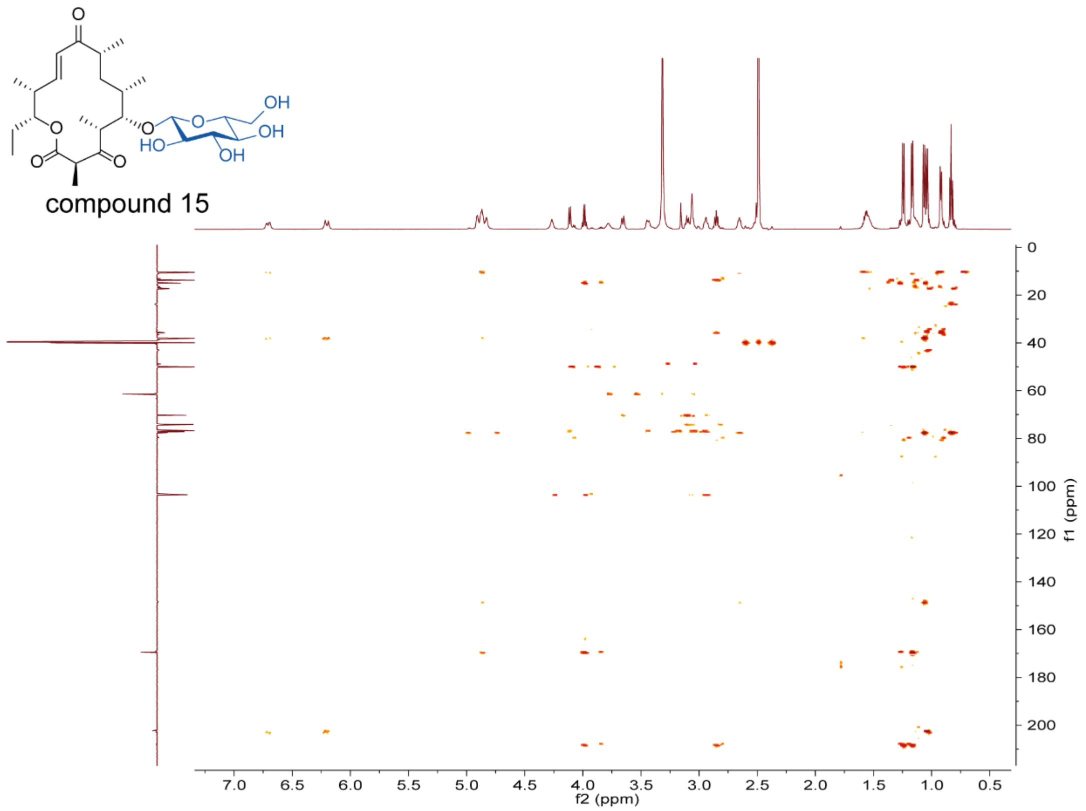

Supplementary Figure 50. HMBC spectrum of compound **15** in DMSO-d6.

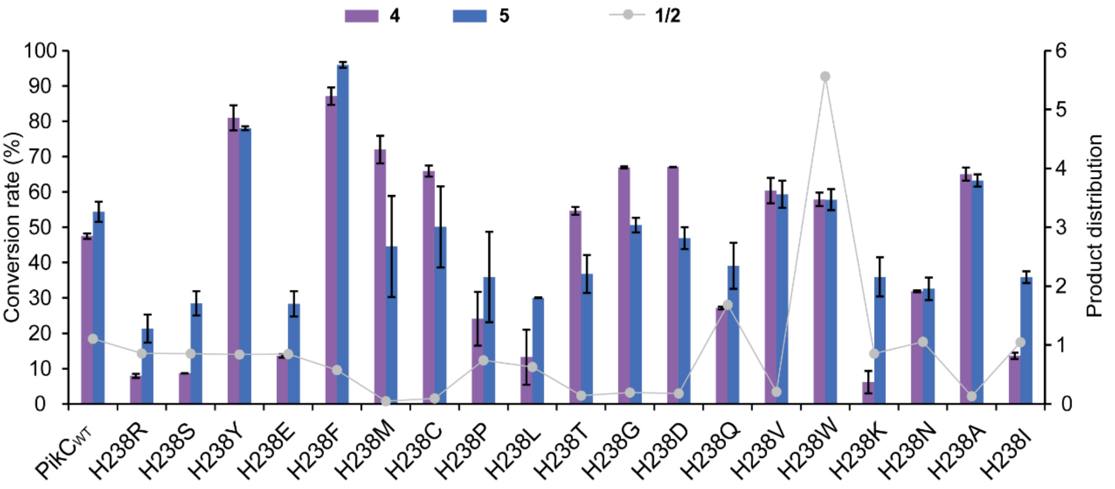

Supplementary Figure 51. The activities of twenty proteinogenic variants of His238 towards YC-17 (**4**) and narbomycin (**5**). Each individual *in vitro* enzymatic reaction containing 1 μM PikC (wild type or mutant), 1 mM NADPH, 10 μM Fdx1499, 5 μM FdR0978, and 0.5 mM substrate in 100 μL storage buffer was incubated at 30 °C for 40 min. Data with error bars represent the average values of duplicated experiments (mean ± SD, n = 2).

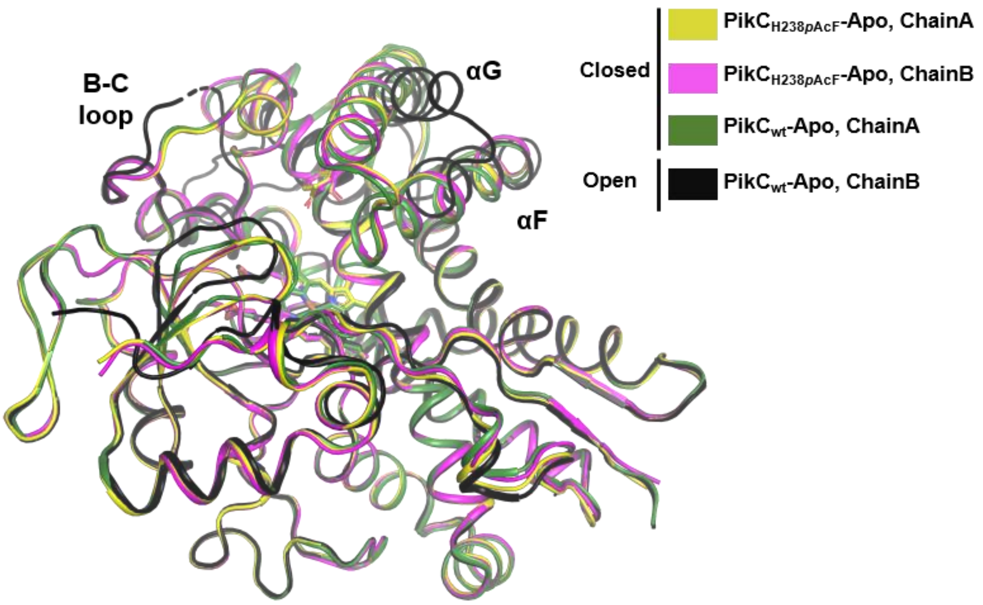

Supplementary Figure 52. Structural superposition of substrate free PikC$_{H238pAcF}$ with PikC$_{WT}$ (PDB ID: 2BVJ). There are two protein chains in the crystallographic asymmetric unit in both PikC$_{H238pAcF}$ and PikC$_{WT}$, and only the Chain B from PikC$_{WT}$ is in open conformation which is defined by the B-C loop and FG helices bent away the substrate binding site. The color codes are shown in the top-right corner.

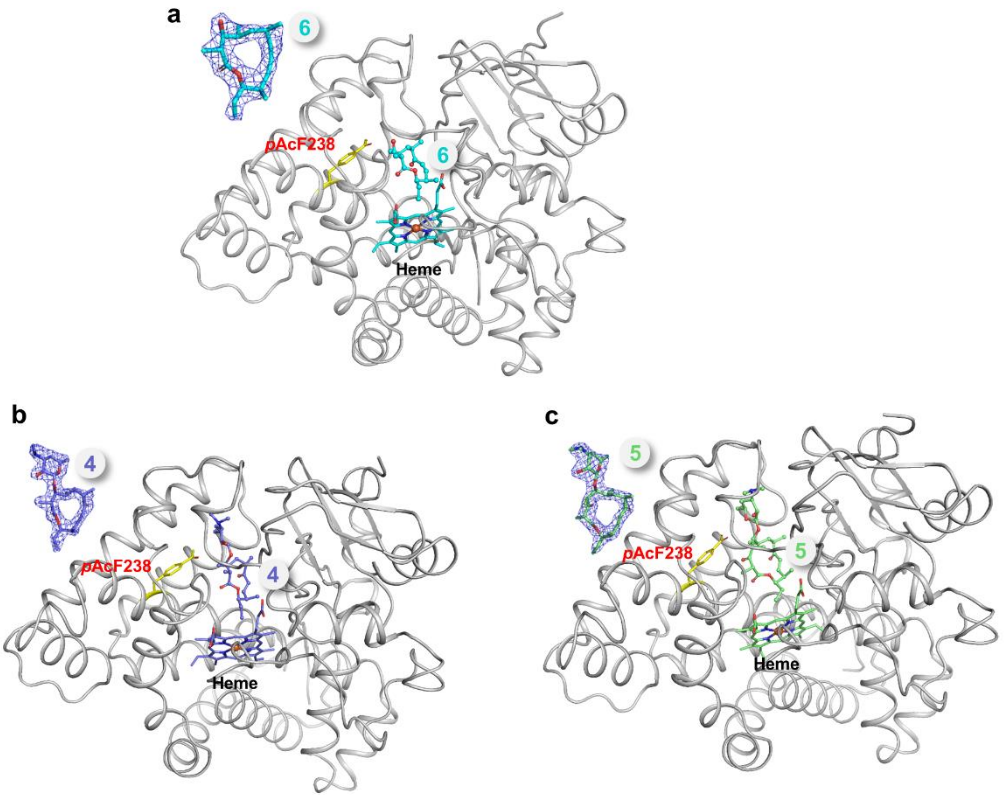

Supplementary Figure 53. **Overall structures of 6-bound, 4-bound, and 5-bound PikC$_{H238pAcF}$**. Ribbon diagrams of the overall crystallographic structures of 6-bound (**a**), 4-bound (**b**), and 5-bound (**c**) PikC$_{H238pAcF}$. The main chains are colored by grey, and the ncAA *p*AcF is shown as yellow stick. 6, 4 and 7molecules are shown as ball-stick and colored by cyan, slate and lime, respectively. The heme groups in these three structures are shown as stick and colored consistent with the corresponding substrates. The substrate molecules are enlarged in the top-left corner and the corresponding weighted 2*Fo*–*Fc* electron density map is contoured at the level of 1.5 σ (blue mesh).

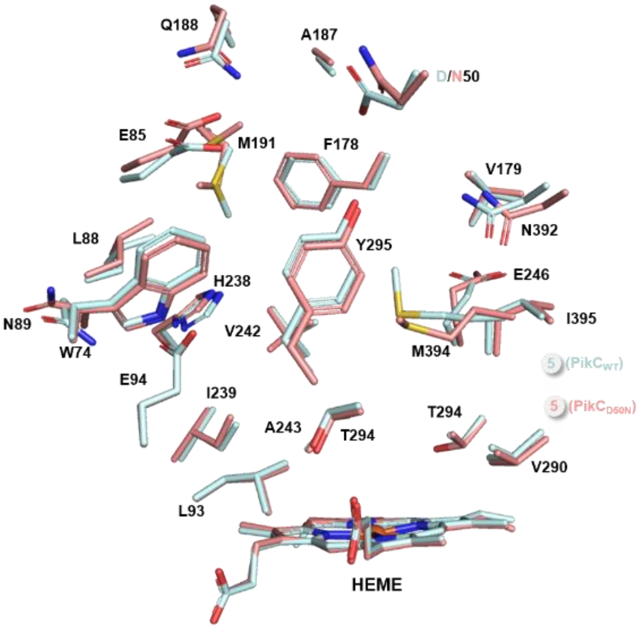

Supplementary Figure 54. Superimposition of the substrate binding sites of 5-bound PikC$_{WT}$ (PDB ID: 2C7X) and 5-bound PikC$_{D50N}$ (PDB ID: 2VZM). The side chains of interacting amino acids within 4.5 Å are shown as sticks and colored in palecyan (2C7X) and salmon (2VZM). For clarity, the bound substrate **5** is removed from the substrate binding sites.

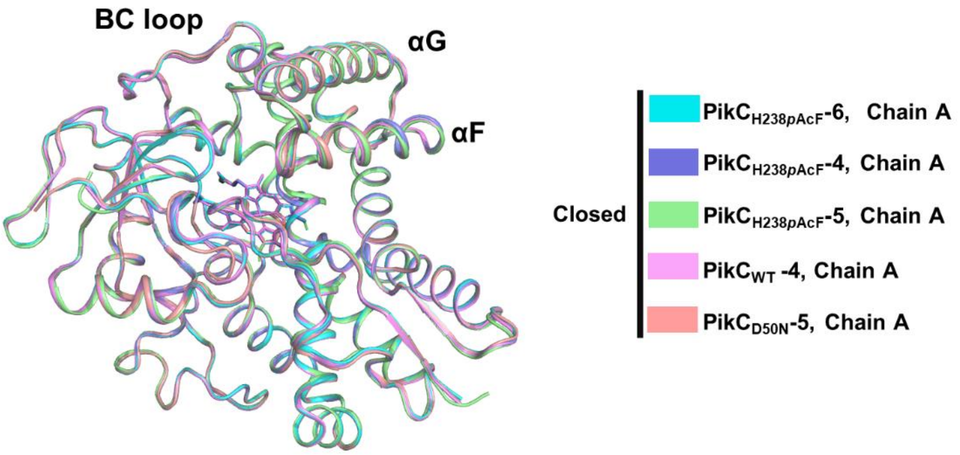

Supplementary Figure 55. Structural superposition of Chain A molecules from6-bound, 4-bound, 5-bound PikC$_{H238pAcF}$ and 4-bound PikC$_{WT}$, 5-bound PikC$_{D50N}$. All these protein chains are in closed conformation. The color codes are shown in the right panel.

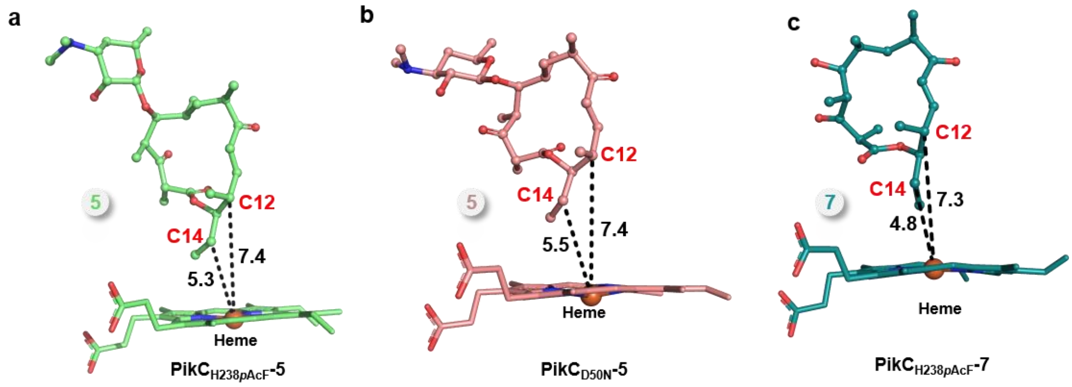

Supplementary Figure 56. The binding modes of the 14-membered ring macrolides. a–**b**, Orientations of narbomycin (NBM, 5) in $PikC_{H238\textit{p}AcF}$ (**a**) and $PikC_{D50N}$ (**b**). The distances from the C12 and C14 atoms to the heme-iron reactive center are labeled in angstrom. **c**, Molecular modeling of narbonolide (NBL, 7) within the substrate-binding pocket of $PikC_{H238\textit{p}AcF}$. The distances from the C12 and C14 atoms to the heme-iron reactive center are labeled in angstrom.

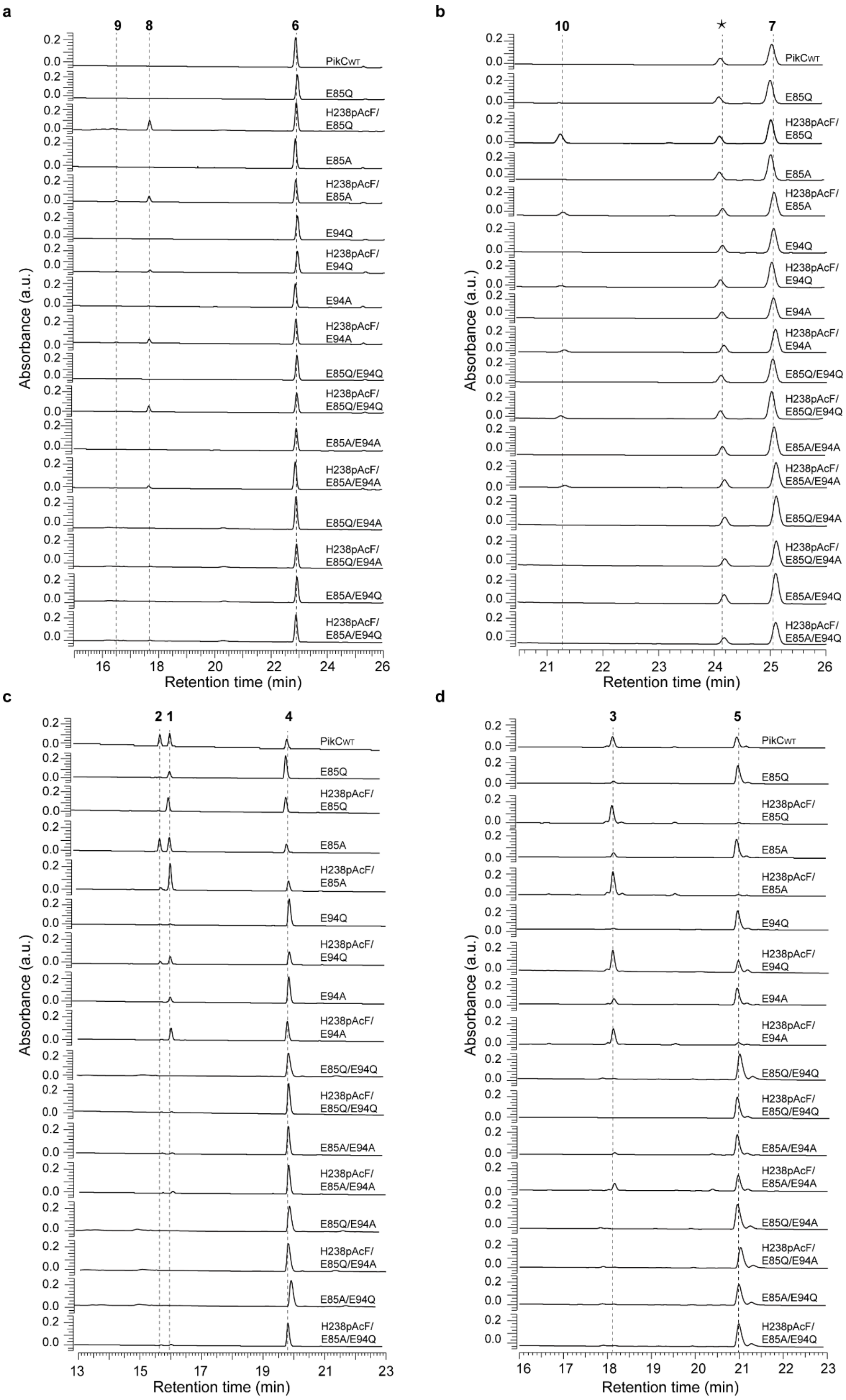

Supplementary Figure 57. HPLC analysis (230 nm) of the enzymatic reactions catalyzed by the wild type (WT) and mutant PikC enzymes using **6** (**a**), **7** (**b**), **4** (**c**) and **5** (**d**) as substrates. The analytical scale reactions containing 1 μM PikC (wild type or mutant), 1 mM NADPH, 10 μM Fdx1499, 5 μM FdR0978, and 0.5 mM substrate in 100 μL storage buffer were incubated at 30 °C for 40 min. The peaks marked by asterisk in **b** denote the spontaneous decomposition product of **7**.

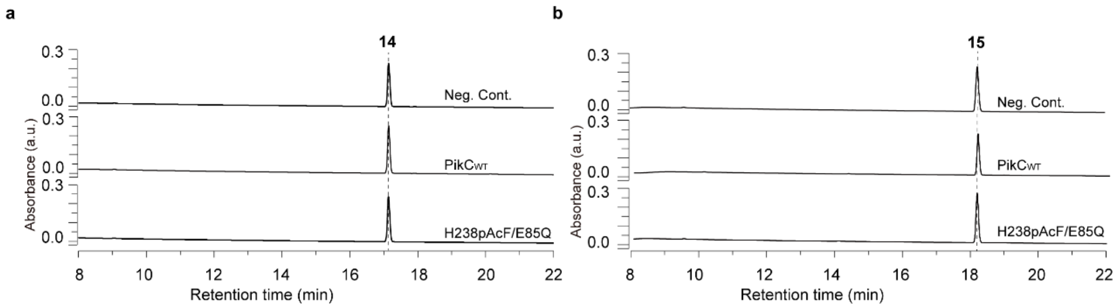

Supplementary Figure 58. HPLC analysis (230 nm) of the enzymatic reactions using **14** (**a**) and **15** (**b**) as substrates. The analytical scale reactions containing 1 μM PikC (wild type or mutant), 1 mM NADPH, 10 μM Fdx1499, 5 μM FdR0978, and 0.5 mM substrate in 100 μL storage buffer were incubated at 30 °C for 40 min.

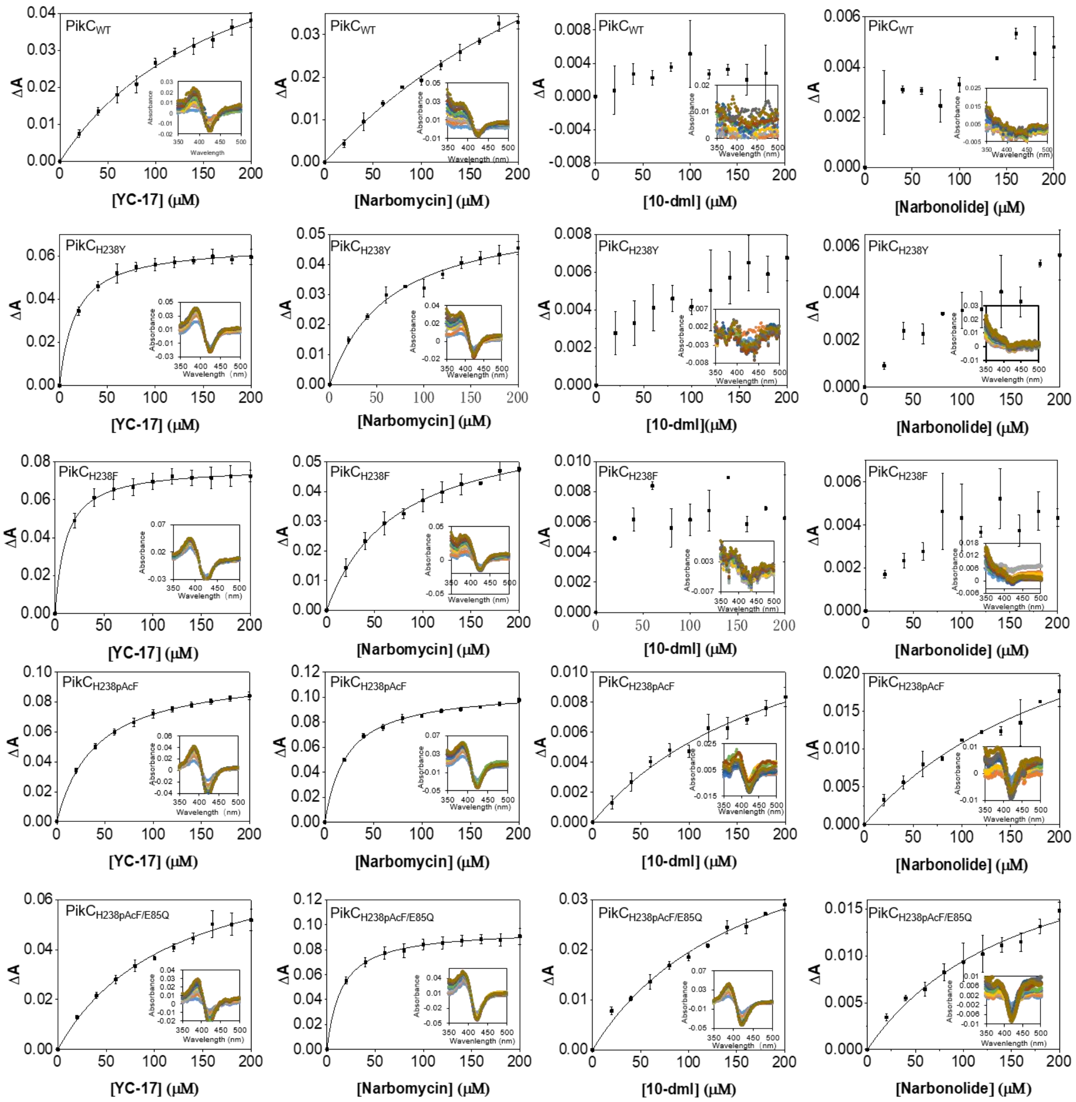

Supplementary Figure 59. Substrate binding curves of different PikC mutants for 10-deoxymethynolide, YC-17, narbomycin and narbonolide. The insets show the type I binding spectra. All experiments were performed in duplicate.

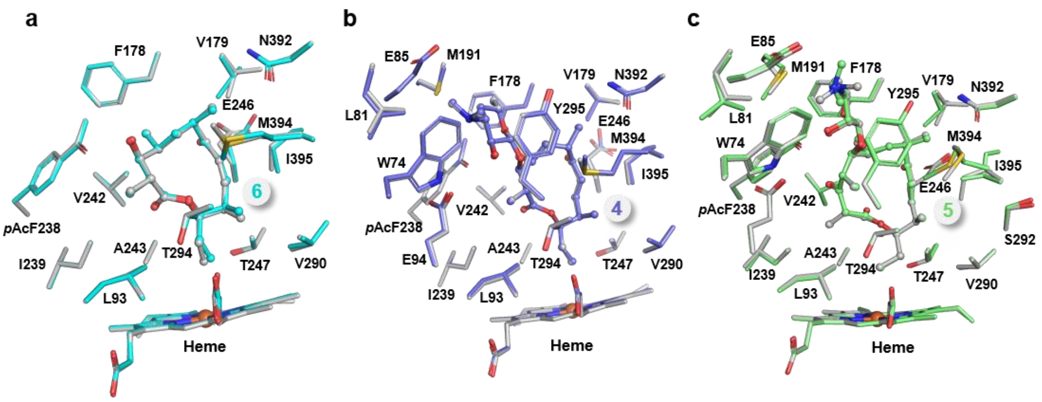

Supplementary Figure 60. Substrate binding sites of chain A and chain B in 6-bound PikC$_{H238pAcF}$, 4-bound PikC$_{H238pAcF}$, and 5-bound PikC$_{H238pAcF}$ structures. Superimposition of substrate binding sites of chain A and chain B in **6**-bound PikC$_{H238pAcF}$ (**a**), **4**-bound PikC$_{H238pAcF}$ (**b**), and **5**-bound PikC$_{H238pAcF}$ (**c**). The side chains of all amino acids within 4.5 Å to the ligands are shown as sticks and colored in grey (chain A) and cyan, slate, and lime (chain B) in these three structures, respectively. The substrates are shown in ball-stick and highlighted in grey (chain A) and cyan, slate, and lime, corresponding to chain B. The heme molecules are shown in sticks and colored consistently with chain A and chain B.

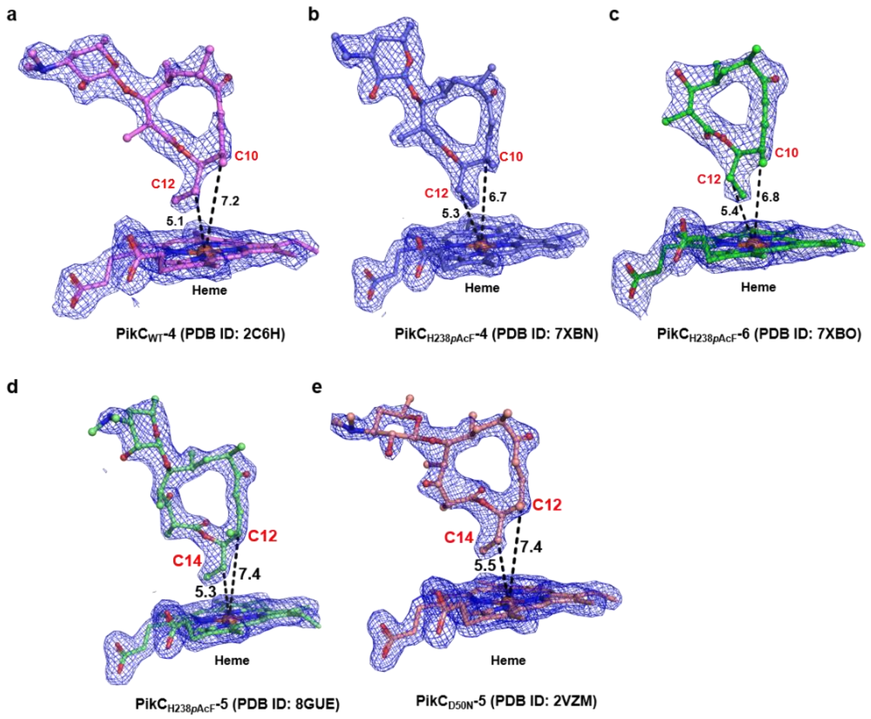

Supplementary Figure 61. 2*Fo–Fc* electron density maps (blue mesh) for the substrates and heme molecules (contoured at 1.5 σ) in PikC$_{WT}$-**4** (**a**), PikC$_{H238pAcF}$-**4** (**b**), PikC$_{H238pAcF}$-**6** (**c**), PikC$_{H238pAcF}$-**5** (**d**), and PikC$_{D50N}$-**5** (**e**). The distances from the C10 (in **4** or **6**, C12 in **5**) and C12 (in **4** or **6**, C14 in **5**) atoms to the heme-iron reactive center are labeled in angstrom.
