# Supplementary material for: Unnatural activities and mechanistic insights of cytochrome P450 PikC gained from site-specific mutagenesis by non-canonical amino acids
Source: Nat Commun. 2023 Mar 25;14:1669. doi: 10.1038/s41467-023-37288-0 (PMC10039885; doi:10.1038/s41467-023-37288-0)
Supplement: Supplementary file 2 — Reporting Summary [file 41467_2023_37288_MOESM2_ESM.pdf]

nature portfolio

Corresponding author(s): Shengying Li

Last updated by author(s): Mar 5, 2023

Reporting Summary

Nature Portfolio wishes to improve the reproducibility of the work that we publish. This form provides structure for consistency and transparency in reporting. For further information on Nature Portfolio policies, see our Editorial Policies and the Editorial Policy Checklist.

Statistics

For all statistical analyses, confirm that the following items are present in the figure legend, table legend, main text, or Methods section.

n/a	Confirmed	
☐	☒	The exact sample size (*n*) for each experimental group/condition, given as a discrete number and unit of measurement
☐	☒	A statement on whether measurements were taken from distinct samples or whether the same sample was measured repeatedly
☐	☒	The statistical test(s) used AND whether they are one- or two-sided<br>*Only common tests should be described solely by name; describe more complex techniques in the Methods section.*
☒	☐	A description of all covariates tested
☒	☐	A description of any assumptions or corrections, such as tests of normality and adjustment for multiple comparisons
☐	☒	A full description of the statistical parameters including central tendency (e.g. means) or other basic estimates (e.g. regression coefficient) AND variation (e.g. standard deviation) or associated estimates of uncertainty (e.g. confidence intervals)
☐	☒	For null hypothesis testing, the test statistic (e.g. *F*, *t*, *r*) with confidence intervals, effect sizes, degrees of freedom and *P* value noted<br>*Give P values as exact values whenever suitable.*
☒	☐	For Bayesian analysis, information on the choice of priors and Markov chain Monte Carlo settings
☒	☐	For hierarchical and complex designs, identification of the appropriate level for tests and full reporting of outcomes
☒	☐	Estimates of effect sizes (e.g. Cohen's *d*, Pearson's *r*), indicating how they were calculated

Our web collection on statistics for biologists contains articles on many of the points above.

Software and code

Policy information about availability of computer code

Data collection	No custom code used<br>Blu-Ice (version 5.0) for X-ray data collection<br>High Resolution Q-TOF mass spectrometry (impact HD) for LCMS data collection<br>Nuclear Magnetic Resonance Spectrometer (AVANCE NEO) for NMR data collection<br>The difference spectra were recorded on a Molecular Devices Spectra Max M2 spectrometer.<br>Peptide samples were eluted on an Ultimate 3000 HPLC (Dionex).<br>Q Exactive mass (Thermo scientific) for data collection of peptide samples<br>The samples were analyzed on an Agilent 1220 HPLC system.

Data analysis	No custom code used<br>Basic statistical analysis was performed using Microsoft Excel 2016 (version 16.0.4266.1001).<br>Figures and plots were made using Adobe Illustrator CC 2021 (version 25.2.1) and PowerPoint 2016 (version 16.0.4266.1001).<br>Geometric structures of compounds were generated by ChemDraw (version 17.0).<br>HKL3000 (version 721.3) was used for X-ray data processing, PHENIX (version 1.19.2-4158) and COOT (version 0.9.6 EL) for structure determination, refinement and model building, and PyMol (version 2.3.2) for preparation of structural figures.<br>DFT optimization was performed for structures of substrates and amino acids with Gaussian16 (Revision A.03).<br>Proteome Discoverer (Thermo, version 1.4) was used for database search.<br>Automated molecular docking was performed by running AutoDock vina (version 1.5.7).

For manuscripts utilizing custom algorithms or software that are central to the research but not yet described in published literature, software must be made available to editors and reviewers. We strongly encourage code deposition in a community repository (e.g. GitHub). See the Nature Portfolio guidelines for submitting code & software for further information.

Data

Policy information about availability of data

All manuscripts must include a data availability statement. This statement should provide the following information, where applicable:

- Accession codes, unique identifiers, or web links for publicly available datasets
- A description of any restrictions on data availability
- For clinical datasets or third party data, please ensure that the statement adheres to our policy

Atomic coordinates and structure factors for substrate-free PikCH238pAcF, 4-bound PikCH238pAcF, 5-bound PikCH238pAcF and 6-bound PikCH238pAcF have been deposited in the Protein Data Bank under the accession numbers 7XBM, 7XBN, 8GUE and 7XBO, respectively. Previously published structures used in this study include PDB IDs: 2BVJ (the crystal structure of ligand-free PikCWT), 2C6H (the crystal structure of 4-bound PikCWT), 2C7X (the crystal structure of 5-bound PikCWT), 2VZ7 (the crystal structure of 4-bound PikCD50N), and 2VZM (the crystal structure of 5-bound PikCD50N). All other relevant data supporting the findings of this study are available in Supplementary Information. Source data are provided as a Source Data file.

Human research participants

Policy information about studies involving human research participants and Sex and Gender in Research.

Reporting on sex and gender	Not applicable
Population characteristics	Not applicable
Recruitment	Not applicable
Ethics oversight	Not applicable

Note that full information on the approval of the study protocol must also be provided in the manuscript.

Field-specific reporting

Please select the one below that is the best fit for your research. If you are not sure, read the appropriate sections before making your selection.

☒ Life sciences ☐ Behavioural & social sciences ☐ Ecological, evolutionary & environmental sciences

For a reference copy of the document with all sections, see nature.com/documents/nr-reporting-summary-flat.pdf

Life sciences study design

All studies must disclose on these points even when the disclosure is negative.

Sample size	Following common practice, the enzyme activities of wild type and each mutants were independently determined in triplicate.
Data exclusions	No data exclusions
Replication	Three independent experiments were conducted, and all attempts at replication were successful. The representative results are displayed in Figure 3b.
Randomization	According to the purpose of the experiments, the samples were allocated to three conditions for testing. Condition 1: the reaction conditions for screening mutants; Condition 2: the reaction conditions for improving substrate conversions; Condition 3: the reaction conditions for glycosylation reactions.
Blinding	All samples were labeled and assayed, and no blinding was applied to the present study.

Reporting for specific materials, systems and methods

We require information from authors about some types of materials, experimental systems and methods used in many studies. Here, indicate whether each material, system or method listed is relevant to your study. If you are not sure if a list item applies to your research, read the appropriate section before selecting a response.

Materials & experimental systems

n/a	Involved in the study	
☒	☐	Antibodies
☒	☐	Eukaryotic cell lines
☒	☐	Palaeontology and archaeology
☒	☐	Animals and other organisms
☒	☐	Clinical data
☒	☐	Dual use research of concern

Methods

n/a	Involved in the study	
☒	☐	ChIP-seq
☒	☐	Flow cytometry
☒	☐	MRI-based neuroimaging
